# Supplementary material for: Pharmacogenetics of Metformin Transporters Suggests No Association with Therapeutic Inefficacy among Diabetes Type 2 Mexican Patients
Source: Pharmaceuticals (Basel). 2022 Jun 22;15(7):774. doi: 10.3390/ph15070774 (PMC9318506; doi:10.3390/ph15070774)
Supplement: Supplementary file 1 [file pharmaceuticals-15-00774-s001.zip › pharmaceuticals-1736492-supplementary.pdf]

Supplementary material

Pharmacogenetics of Metformin Transporters Suggests No Association with Therapeutic Inefficacy Among Diabetes Type 2 Mexican Patients

Content.

S1. 1 Table 1 analysis.

S1.2 Hardy-Weinberg equilibrium.

S1.3 Frequencies in controlled and uncontrolled patients according to genotype.

S1.4 Metformin plasmatic concentrations among DMT2 patients according to SNP and genotype.

S1.5 Metformin plasmatic concentrations among DMT2 patients according to treatment and genotype.

S1.6 Glycated Hemoglobin (HbA1c) among DMT2 patients according to treatment and genotype.

S1.7 Dominant models.

S.8 Diplotype analysis.

S1.9 Metformin dose and genotype.

S1.10 Figure: Linear regression.

S1.11 Statistical results and procedures.

- S1.11.1 Table 1.
- S1.11.2 Hardy Weinberg Equilibrium.
- S1.11.3 Table 3.
- S1.11.4 Metformin plasmatic concentrations among DMT2 patients according to SNP and genotype.
- S1.11.5 Metformin concentration according to treatment and genotype.
- S1.11.6 Glycated Hemoglobin (HbA1c) according to treatment and genotype (n=103).
- S1.11.7 Dominant models: *OCT1* and *ABCB1*.
- S1.11.8 Daily metformin dose mg/kg/day among DMT2 according to treatment and genotype).

S1.1 Table 1 analysis.

Group by treatment (Metformin or Metformin + Glibenclamide).

Normality test.

- Cements the statistical inference test.
- Use of Kolmogorov Smirnov test (50 or more observations) or the Shapiro-Wilk test (Less than 50 observations).

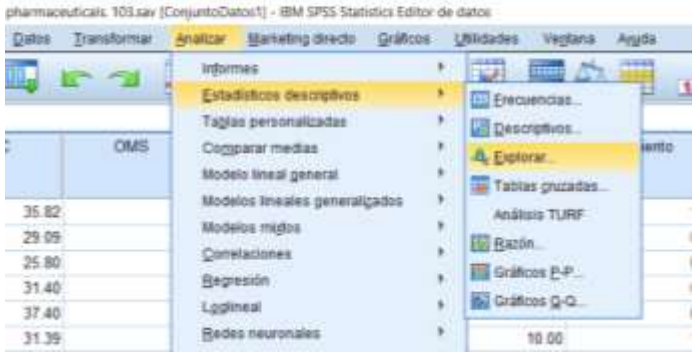

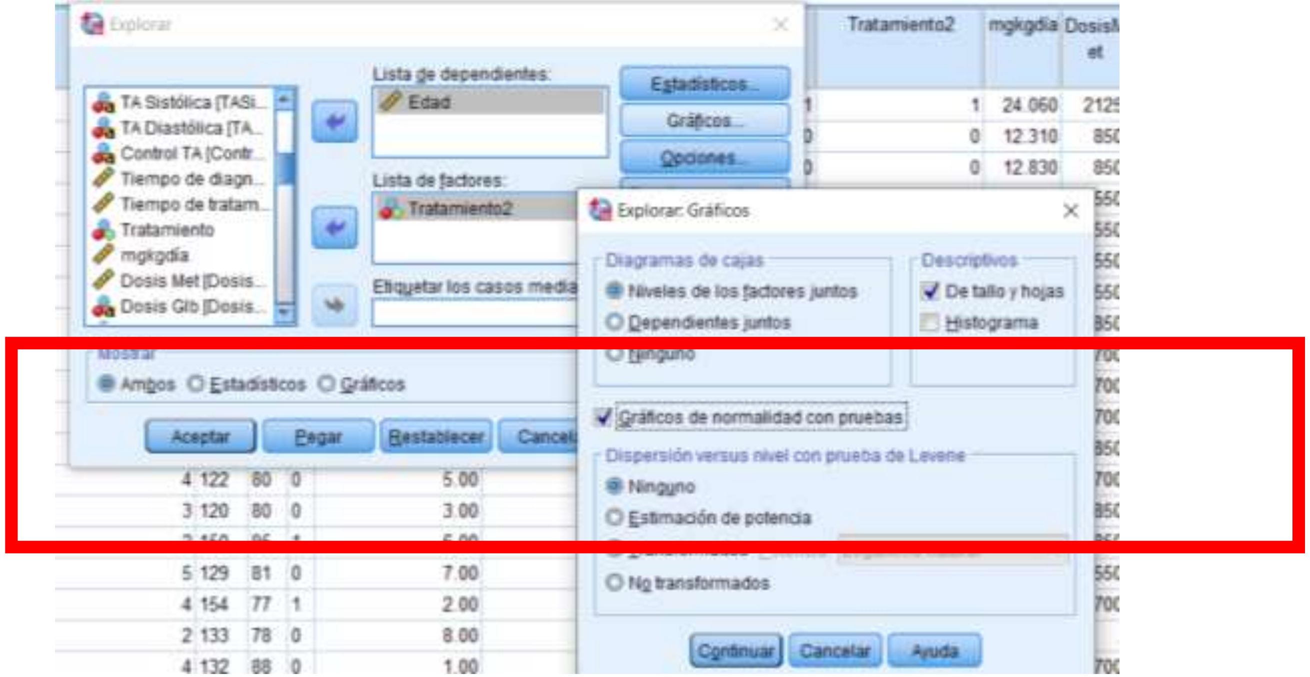

Hypothesis testing.

- H0: Data distribution = Normal distribution ($p > 0.05$).
- H1: Data distribution ≠ Normal distribution ($p < 0.05$).

- Descriptive analysis:

- Normal distribution: Mean and standard deviation.
- Non-normal distribution: Median and interquartile range.

Analyzed data (n= 103).

Resumen de procesamiento de casos

	Tratamiento2	Casos					
		Válido		Perdidos		Total	
		N	Porcentaje	N	Porcentaje	N	Porcentaje
Edad	Metformina	59	100.0%	0	0.0%	59	100.0%
	Met + Glb	44	100.0%	0	0.0%	44	100.0%

Normality test.

Pruebas de normalidad

	Tratamiento2	Kolmogorov-Smirnov[a]			Shapiro-Wilk		
		Estadístico	gl	Sig.	Estadístico	gl	Sig.
Edad	Metformina	.069	59	.200*	.990	59	.894
	Met + Glb	.097	44	.200*	.973	44	.390

*. Esto es un límite inferior de la significación verdadera.

a. Corrección de significación de Lilliefors

Descriptive data.

Comparison of metformin monotherapy and metformin + glibenclamide.

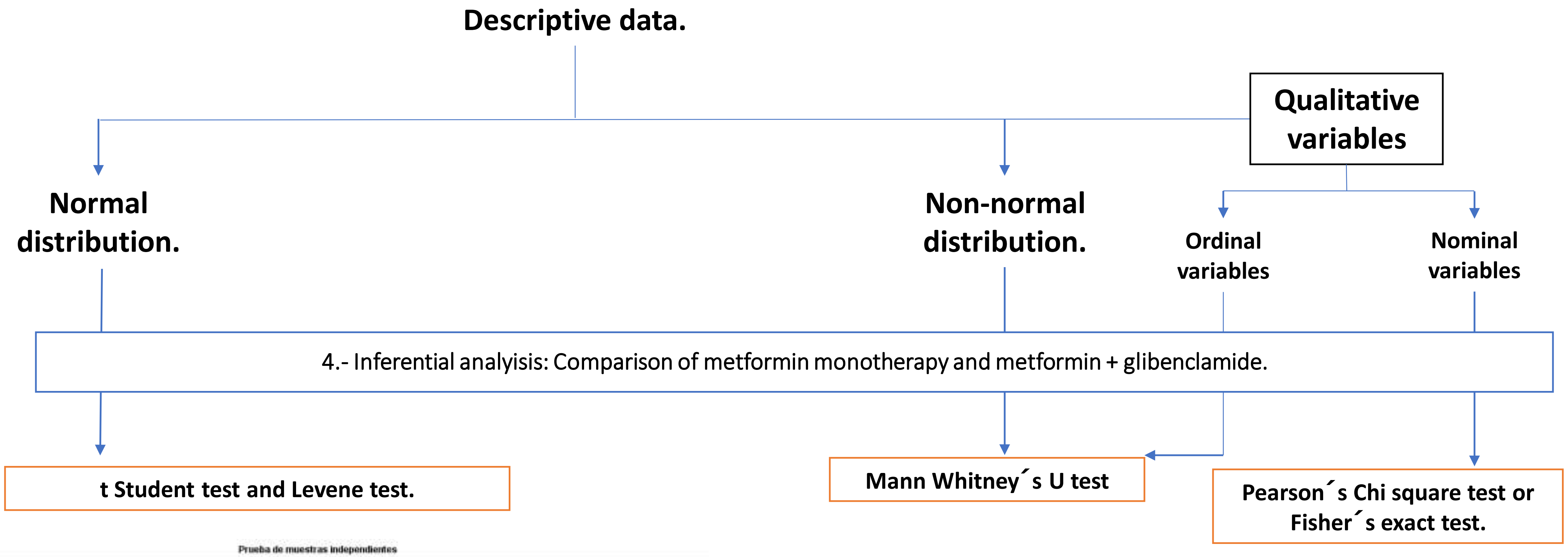

Prueba de muestras independientes

		Prueba de Levene de igualdad de varianzas		prueba t para la igualdad de medias						
									95% de intervalo de confianza de la diferencia	
		F	Sig.	t	gl	Sig. (bilateral)	Diferencia de medias	Diferencia de error estándar	Inferior	Superior
Edad	Se asumen varianzas iguales	.538	.465	1.221	101	.225	2.683	2.197	-1.675	7.041
	No se asumen varianzas iguales			1.214	90.674	.228	2.683	2.210	-1.707	7.073

Estadísticos de prueba[a]

	OMS
U de Mann-Whitney	1041.000
W de Wilcoxon	2031.000
Z	-1.785
Sig. asintótica (bilateral)	.074

a. Variable de agrupación: Tratamiento2

Pruebas de chi-cuadrado

	Valor	gl	Significación asintótica (bilateral)	Significación exacta (bilateral)	Significación exacta (unilateral)
Chi-cuadrado de Pearson	38.330[a]	1	.000		
Corrección de continuidad[b]	35.098	1	.000		
Razón de verosimilitud	42.192	1	.000		
Prueba exacta de Fisher				.000	.000
Asociación lineal por lineal	37.958	1	.000		
N de casos válidos	103				

a. 0 casillas (0.0%) han esperado un recuento menor que 5. El recuento mínimo esperado es 20.50.

b. Sólo se ha calculado para una tabla 2x2

Results

- The results obtained by the SPSS statistical program are found in **S1.11.1.**

S1.2 Hardy-Weinberg equilibrium.

Genotypic and allelic frequencies.

Allelic proportion (Biallelic cases).

- $AP\ (Allelic\ proportion) = \frac{Number\ of\ alleles}{Total\ alleles}$

- Example for rs72552763:
- GAT/GAT= 69, del/GAT= 27, del/del=7

GAT proportion	Del proportion
$AP = \frac{165}{206} = 0.80097087$	$AP = \frac{41}{206} = 0.19902913$

- **Sustitution in: $(p+q)^2 = p^2 + 2(p*q) + q^2$ (Biallelic cases).**
- **Where:**
- **GAT proportion= p**
- **Del proportion= q**

Allelic proportion (Triallelic cases).

- $AP\ (Allelic\ proportion) = \frac{Number\ of\ alleles}{Total\ alleles}$

- Example for rs2032582:
- G/G= 85, G/T= 10, T/T= 0, T/A=1, G/A= 7

G Proportion	T Proportion	A Proportion
$AP = \frac{187}{206} = 0.90776699$	$AP = \frac{11}{206} = 0.05339806$	$AP = \frac{8}{206} = 0.03883495$

- **Sustitution in: $(p+q+r)^2 = p^2 + 2(p*q) + q^2 + 2qr + r^2$ (Triallelic cases).**
  - **Where:**
  - **G proportion= p**
  - **T proportion= q**
  - **A Proportion= r**

Genotypic and allelic frequencies among DMT2 patients (n= 103).

1.- Patients with genotypic and allelic frequencies (n=103).

Biallelic polymorphisms.

Triallelic polymorphisms.

2.- Calculation of expected frequencies.

$$(p+q)^{2} = p^{2} + 2\,(p*q) + q^{2}$$

$$(p+q+r)^{2} = p^{2} + 2pq + q^{2} + 2\,pr + 2qr + r^{2}$$

3.- Pearson´s Xi square test for Hardy-Weinberg equilibrium.

GENOTIPO

	N observado	N esperada	Residuo
GAT7GAT	44	43.6	.4
del/GAT	46	46.8	-.8
del/del	13	12.6	.4
Total	103		

Estadísticos de prueba

	GENOTIPO
Chi-cuadrado	.033[a]
gl	2
Sig. asintótica	.984

a. 0 casillas (0.0%) han esperado frecuencias menores que 5. La frecuencia mínima de casilla esperada es 12.6.

GENOTIPO

	N observado	N esperada	Residuo
*1/*1	85	85.3	-.3
*1/*2	10	10.0	.0
*2/*3	1	.4	.6
*1/*3	7	7.3	-.3
Total	103		

Estadísticos de prueba

	GENOTIPO
Chi-cuadrado	.805[a]
gl	3
Sig. asintótica	.848

a. 1 casillas (25.0%) han esperado frecuencias menores que 5. La frecuencia mínima de casilla esperada es .4.

Pearson´s Chi square.

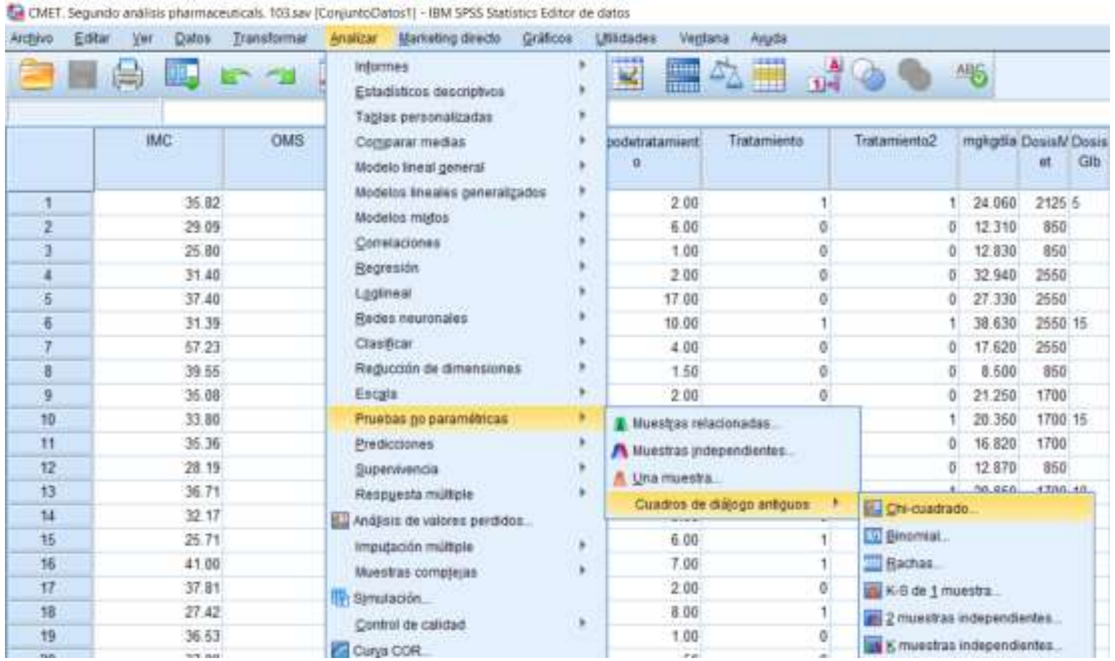

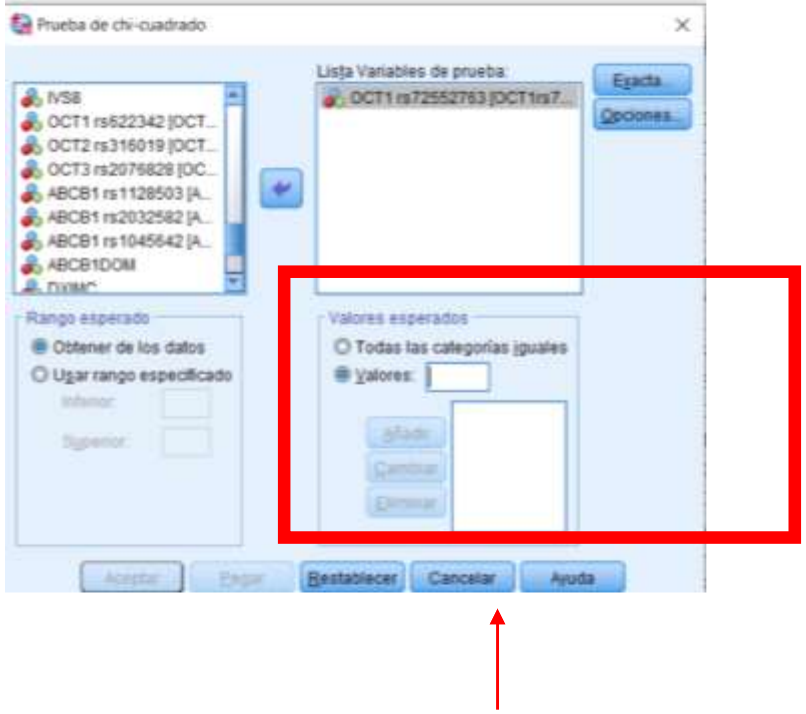

Expected frequencies.

Results.

- The Hardy Weinberg equilibrium results are found in **S1.11.2 Hardy Weinberg Equilibrium.**

S1.3 Frequencies in controlled and uncontrolled patients according to genotype.

Pearson´s Chi square.

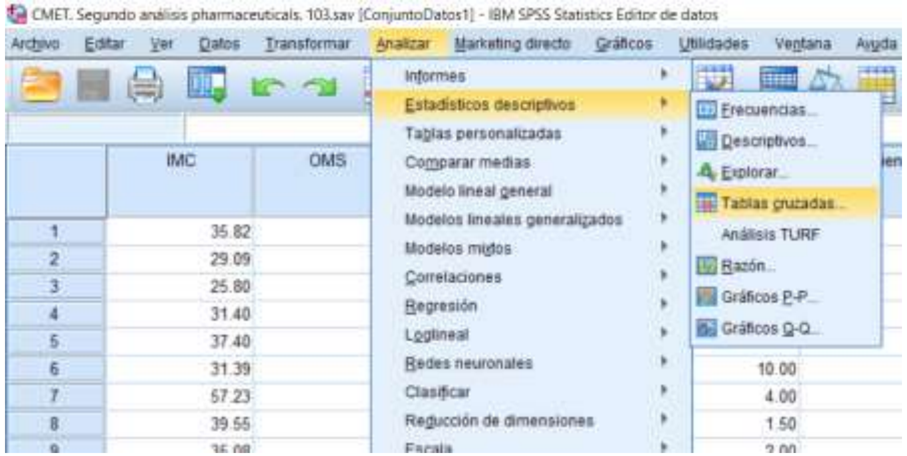

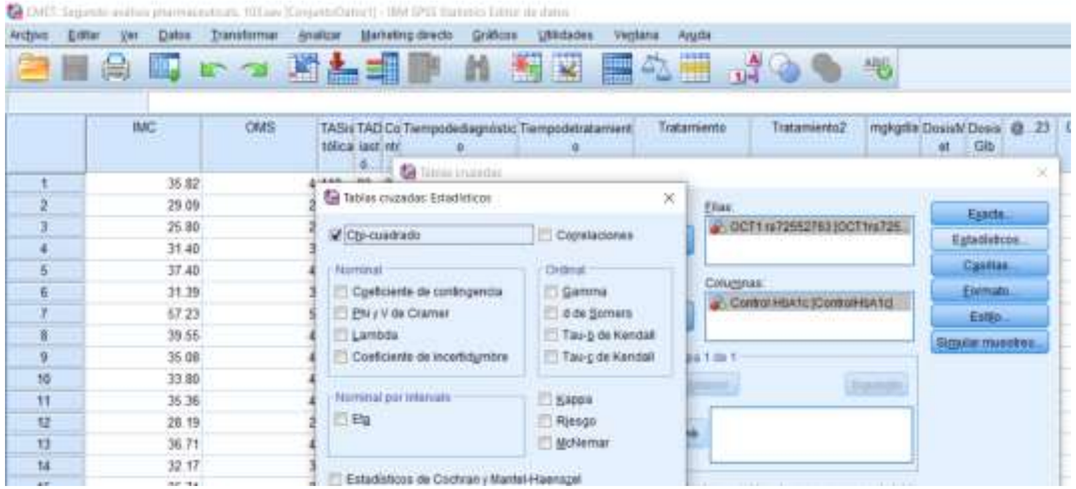

Therapeutic efficacy according to SNP and DMT2 patient´s genotype.

Polymorphism carriers sirted according to control or non control defined by HbA1c (n= 103).

SLCA22A1: rs72552763 and rs622342.

SLCA22A2: rs316019.

SLC22A3: rs2076828.

ABCB1: rs1128503, rs2032582 and rs1045642.

CYP2C9: rs1799853, rs1057910 and rs1934969.

Control (HbA1c <7%).

Non- control (HbA1c ≥7%).

Pearson´s Chi square.

Tabla cruzada OCT1 rs72552763*Control HbA1c

			Control HbA1c		Total
			Si control	No control	
OCT1 rs72552763	GAT/GAT	Recuento	23	21	44
		% dentro de Control HbA1c	47.9%	38.2%	42.7%
	del/GAT	Recuento	18	28	46
		% dentro de Control HbA1c	37.5%	50.9%	44.7%
	del/del	Recuento	7	6	13
		% dentro de Control HbA1c	14.6%	10.9%	12.6%
Total		Recuento	48	55	103
		% dentro de Control HbA1c	100.0%	100.0%	100.0%

Pruebas de chi-cuadrado

	Valor	gl	Significación asintótica (bilateral)
Chi-cuadrado de Pearson	1.875[a]	2	.392
Razón de verosimilitud	1.883	2	.390
Asociación lineal por lineal	.201	1	.654
N de casos válidos	103		

a. 0 casillas (0.0%) han esperado un recuento menor que 5. El recuento mínimo esperado es 6.06.

Results.

- The results produced by the SPSS program are found in **S1.11.3 Table 3.**

S1.4 Metformin plasmatic concentrations according to SNP and genotype.

Group by: Control or non-control.

Metformin plasmatic concentrations among DMT2 patients according to SNP and genotype.

SLCA22A1: rs72552763 and rs622342.

SLCA22A2: rs316019.

SLC22A3: rs2076828.

ABCB1: rs1128503, rs2032582 and rs1045642.

CYP2C9: rs1799853, rs1057910 and rs1934969.

Control (HbA1c <7%)

Non- control (HbA1c ≥7%).

Descriptive analysis.

Metformin plasmatic concentration.

Resumen de procesamiento de casos

OCT1 rs72552763	Control HbA1c		Casos: Válido N	Válido Porcentaje	Perdidos N	Perdidos Porcentaje	Total N	Total Porcentaje
GAT/GAT	Si control	CONCMET	17	73.9%	6	26.1%	23	100.0%
	No control	CONCMET	17	81.0%	4	19.0%	21	100.0%
del/GAT	Si control	CONCMET	14	77.8%	4	22.2%	18	100.0%
	No control	CONCMET	27	96.4%	1	3.6%	28	100.0%
del/del	Si control	CONCMET	6	85.7%	1	14.3%	7	100.0%
	No control	CONCMET	5	83.3%	1	16.7%	6	100.0%

Normality test.

Pruebas de normalidad

OCT1 rs72552763	Control HbA1c		Kolmogorov-Smirnov[a] Estadístico	gl	Sig.	Shapiro-Wilk Estadístico	gl	Sig.
GAT/GAT	Si control	CONCMET	.206	17	.003	.775	17	.001
	No control	CONCMET	.148	17	.200*	.915	17	.121
del/GAT	Si control	CONCMET	.313	14	.001	.598	14	.000
	No control	CONCMET	.137	27	.200*	.844	27	.001
del/del	Si control	CONCMET	.387	6	.004	.687	6	.001
	No control	CONCMET	.226	5	.200*	.875	5	.288

*. Este es un límite inferior de la significación verdadera.

a. Corrección de significación de Lilliefors

Descriptive data.

Percentiles

OCT1 rs72552763	Control HbA1c			Percentiles 5	10	25	50	75	90	95
GAT/GAT	Si control	Promedio ponderado (Definición 1)	CONCMET	62.868000	92.609000	129.834950	376.983900	819.813000	2172.083800	.
		Bisagras de Tukey	CONCMET			133.472000	376.983900	763.070000		
	No control	Promedio ponderado (Definición 1)	CONCMET	45.940000	87.517600	169.083000	481.759000	1000.998000	1300.518400	.
		Bisagras de Tukey	CONCMET			185.934000	481.759000	971.008000		
del/GAT	Si control	Promedio ponderado (Definición 1)	CONCMET	34.154000	34.752485	61.853000	137.112000	719.343250	3236.361000	.
		Bisagras de Tukey	CONCMET			66.470000	137.112000	667.450000		
	No control	Promedio ponderado (Definición 1)	CONCMET	14.537600	25.692200	210.800900	790.618000	1055.678000	1450.158000	2676.207000
		Bisagras de Tukey	CONCMET			228.851400	790.618000	1030.222500		
del/del	Si control	Promedio ponderado (Definición 1)	CONCMET	80.594990	80.594990	100.922173	139.152950	528.639925	.	.
		Bisagras de Tukey	CONCMET			107.697900	139.152950	264.307900		
	No control	Promedio ponderado (Definición 1)	CONCMET	22.222000	22.222000	46.232000	785.648000	1784.910000	.	.
		Bisagras de Tukey	CONCMET			70.242000	785.648000	1642.771000		

Inferential analysis.

Inferential analysis.

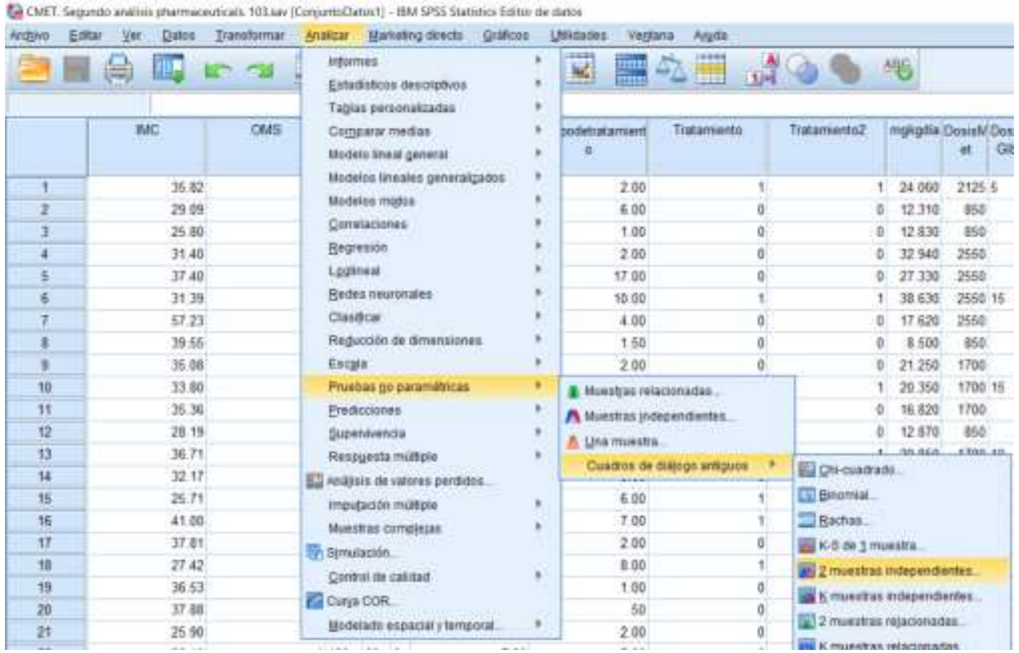

MANN WHITNEY´S U TEST.

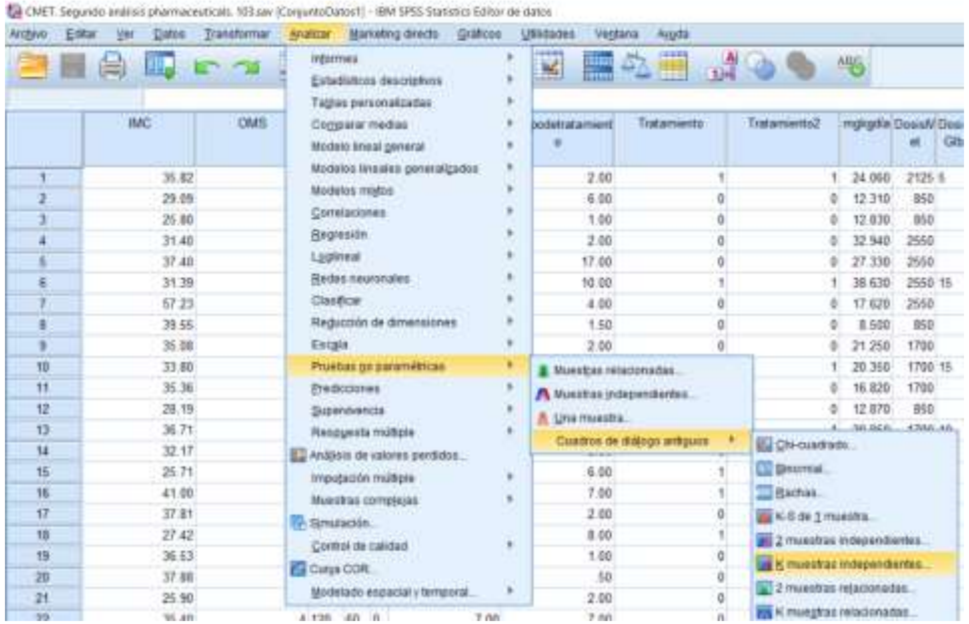

KRUSKAL WALLIS TEST.

Table 4. Metformin plasmatic concentrations among DMT2 patients grouped by SNP and genotype.

Gene	SNP	Genotype	Control (ng/mL) median (IQR 25-75%) [n]	Non-control (ng/mL) median (IQR 25-75%) [n]	*P=
		GAT/GAT	376.9 (133.4 – 763) [17]	481.7 (185.9 – 971) [17]	0.812
	rs72552763	del/GAT	137.1 (66.47 687.4) [14]	790.6 (228.8 – 1030.2) [27]	0.115
		del/del	139.1 (107.6 – 264.3) [6]	785.6 (70.2 – 1642.7) [5]	0.792
SLC22A1 (OCT1)		*P=	0.358	0.820	
		A/A	339.7 (129.8 – 700.9) [16]	526.3 (169 – 1000.9) [16]	0.616

Results.

- The results produced by the SPSS program are found in **S1.11.4 Metformin plasmatic concentrations among DMT2 patients according to SNP and genotype.**

S1.5 Metformin plasmatic concentrations according to treatment and genotype.

Group by Treatment: Metformin or Metformin + Glibenclamide.

Metformin plasmatic concentrations among DMT2 patients grouped by treatment and genotype.

SLCA22A1: rs72552763 and rs622342.

SLCA22A2: rs316019.

SLC22A3: rs2076828.

ABCB1: rs1128503, rs2032582 and rs1045642.

CYP2C9: rs1799853, rs1057910 and rs1934969.

Metformin

Metformin + Glibenclamide

Descriptive analysis.

Metformin plasmatic concentration.

Resumen de procesamiento de casos

OCT1 rs72552763	Control HbA1c		Casos: Válido N	Válido Porcentaje	Perdidos N	Perdidos Porcentaje	Total N	Total Porcentaje
GAT/GAT	Si control	CONCMET	17	73.9%	6	26.1%	23	100.0%
	No control	CONCMET	17	81.0%	4	19.0%	21	100.0%
del/GAT	Si control	CONCMET	14	77.8%	4	22.2%	18	100.0%
	No control	CONCMET	27	96.4%	1	3.6%	28	100.0%
del/del	Si control	CONCMET	6	85.7%	1	14.3%	7	100.0%
	No control	CONCMET	5	83.3%	1	16.7%	6	100.0%

Normality test.

Pruebas de normalidad

OCT1 rs72552763	Control HbA1c		Kolmogorov-Smirnov[a] Estadístico	gl	Sig.	Shapiro-Wilk Estadístico	gl	Sig.
GAT/GAT	Si control	CONCMET	.206	17	.003	.775	17	.001
	No control	CONCMET	.148	17	.200*	.915	17	.121
del/GAT	Si control	CONCMET	.313	14	.001	.886	14	.060
	No control	CONCMET	.137	27	.200*	.844	27	.001
del/del	Si control	CONCMET	.387	6	.004	.687	6	.001
	No control	CONCMET	.226	5	.200*	.875	5	.286

* Este es un límite inferior de la significación verdadera.

a. Corrección de significación de Lilliefors

Descriptive data.

Percentiles

OCT1 rs72552763	Control HbA1c			Percentiles 5	10	25	50	75	90	95
GAT/GAT	Si control	Promedio ponderado (Definición 1)	CONCMET	62.868000	92.609000	129.834950	376.983900	819.813000	2172.083800	.
		Bisagras de Tukey	CONCMET			133.472000	376.983900	763.070000		
	No control	Promedio ponderado (Definición 1)	CONCMET	45.940000	87.517600	169.003000	481.759000	1000.998000	1300.518400	.
		Bisagras de Tukey	CONCMET			185.934000	481.759000	971.008000		
del/GAT	Si control	Promedio ponderado (Definición 1)	CONCMET	34.154000	34.752495	61.853000	137.112000	719.343250	3235.361000	.
		Bisagras de Tukey	CONCMET			68.470000	137.112000	667.450000		
	No control	Promedio ponderado (Definición 1)	CONCMET	14.537600	25.692200	210.800900	780.618000	1055.678000	1450.158000	2676.207000
		Bisagras de Tukey	CONCMET			228.851400	780.618000	1030.222500		
del/del	Si control	Promedio ponderado (Definición 1)	CONCMET	80.594990	80.594990	100.922173	139.152950	528.638925	.	.
		Bisagras de Tukey	CONCMET			107.697900	139.152950	264.307900		
	No control	Promedio ponderado (Definición 1)	CONCMET	22.222000	22.222000	46.232000	785.648000	1764.918000	.	.
		Bisagras de Tukey	CONCMET			70.242000	785.648000	1642.771000		

Inferential analysis.

Inferential analysis.

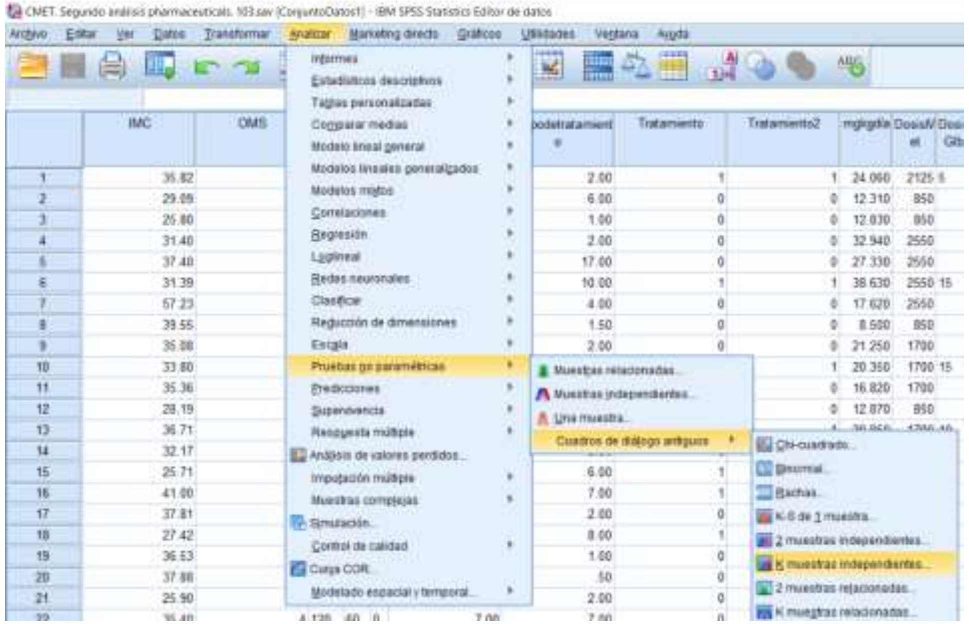

KRUSKAL WALLIS TEST.

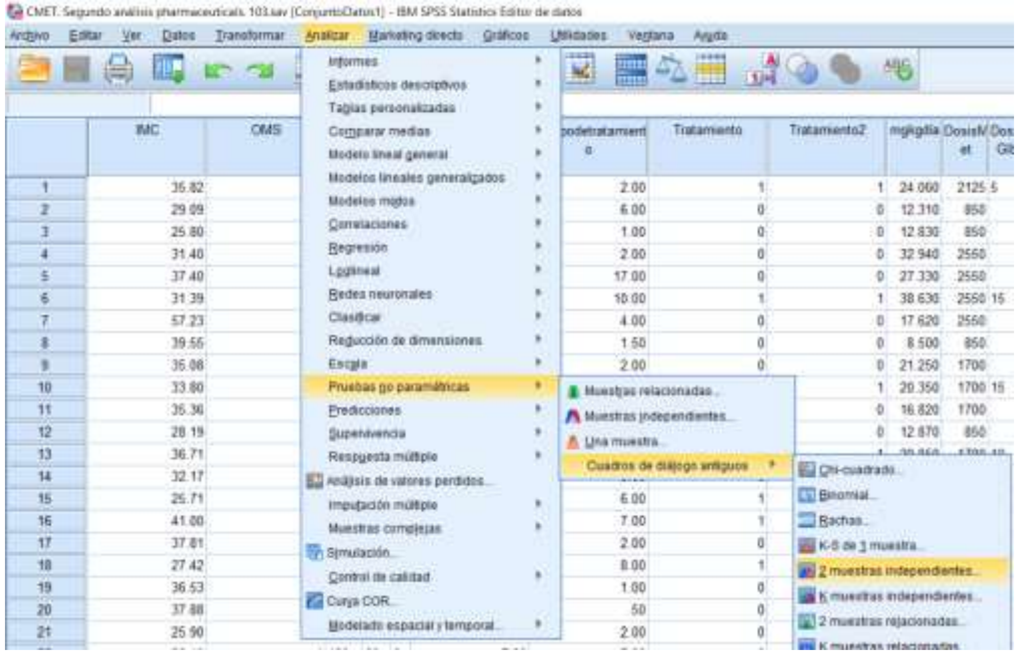

MANN WHITNEY´S U TEST.

Table 5. Metformin plasmatic concentrations among DMT2 patients grouped by treatment and genotype.

Gene	SNP	Genotype	Metformin (ng/mL) median (IQR 25-75%) [n]	Metformin+Glibenclamide (ng/mL) median (IQR 25-75%) [n]	P=
		GAT/GAT	504 (129.8 – 923.7) [20]	366 (185.9 – 731) [14]	0.861
	rs72552763	del/GAT	262.1 (57.2 – 765.1) [19]	797.3 (183.3 – 1088.9) [22]	0.067
		del/del	146.1 (119.9 – 792.9) [7]	427.9 (46.2 – 1336.3) [4]	0.705
SLC22A1		P=	0.463	0.459	
(OCT1)		A/A	451.6 (126.1 – 876) [18]	366 (185.9 – 731) [14]	0.939

Table S1.5.1 Metformin plasmatic concentrations among DMT2 patients grouped by treatment and genotype.

Table S1.5.1 Metformin plasmatic concentrations among DMT2 patients grouped by treatment and genotype.

Gene	SNP	Genotype	Metformin (ng/mL) median (IQR 25-75%) [n]	Metformin+Glibenclamide (ng/mL) median (IQR 25-75%) [n]	P=
SLC22A1 (OCT1)	rs72552763	GAT/GAT	504 (129.8 – 923.7) [20]	366 (185.9 – 731) [14]	0.861
		del/GAT	262.1 (57.2 – 765.1) [19]	797.3 (183.3 – 1088.9) [22]	0.067
		del/del	146.1 (119.9 – 792.9) [7]	427.9 (46.2 – 1336.3) [4]	0.705
		P=	0.463	0.459	
	rs622342	A/A	451.6 (126.1 – 876) [18]	366 (185.9 – 731) [14]	0.939
		A/C	270.5 (57.2 – 770) [16]	790.6 (215.1 – 1030.2) [19]	0.154
		C/C	237.5 (119.9 – 1063.4) [12]	785.6 (114 – 1994.8) [7]	0.447
		P=	0.639	0.682	
SLCA22A2 (OCT2)	rs316019	C/C	327.9 (105.2 – 815) [42]	511 (157.8 – 1030.9) [38]	0.272
		A/C	283.4 (237 – 589.5) [4]	1025 (804 – 1247.5) [2]	0.165
		A/A	[0]	- [0]	-
		P=	0.755	0.238	
SLC22A3 (OCT3)	rs2076828	C/C	146.1 (102.6 – 810.1) [35]	608.2 (146 – 1055.6) [30]	0.206
		C/G	481.7 (264.3 – 687.4) [9]	551.2 (268.7 – 939.7) [8]	0.564
		G/G	1482.2 (1321.6 – 1642.7) [2]	723.9 (416.8 – 1030.9) [2]	0.121
		P=	0.120	0.878	
ABCB1	rs1128503	C/C	815 (441.5 – 1482.2) [11]	344.2 (126.9 – 897.6) [12]	0.124
		C/T	282.3 (98.9 – 765.1) [24]	994.5 (731 – 1344.1) [14] ¥	**0.002***
		T/T	142.5 (113.9 – 402.6) [11]	210.2 (85.7 – 530.7) [14]	0.511
		P=	0.075	**0.007** ×	
	rs2032582	G/G	618.8 (87 – 1068.3) [15]	788.1 (219.8 – 1030.9) [14]	0.513
		G/T	282.3 (100 – 805.3) [22]	416.8 (185.9 – 685.7) [13]	0.339
		T/T	278.9 (176.6 – 606.8) [7]	478 (58 – 1472.3) [8]	1.000
		T/A	1348.7 [1]	3223.2 [1]	-
		G/A	105.28 [1]	611 (428.9 – 1158.2) [3]	-
		P=	0.884	0.442	
	rs1045642	C/C	618.8 (99.9 – 789) [11]	837.4 (441.6 – 1055.6) [18]	0.164
		C/T	139.1 (98.9 – 765.1) [24]	308.7 (157.8 – 685.7) [14]	0.204
		T/T	526.3 (271.6 – 888.8) [11]	221.3 (58 – 946.5) [8]	0.322
		P=	0.258	0.175	
CYP2C9	rs1799853 (*2) rs1057910 (*3)	*1/*1	376.9 (109.6 – 888.8) [39]	685.7 (210.2 – 1043.3) [31]	0.244
		*1/*2	290.7 (100 – 660.5) [6]	96.8 (78 – 126.9) [4] ¥	0.201
		*2/*2	- [0]	- [0]	-
		*1/*3	262.76 [1]	712.3 (324.6 – 2078.7) [4]	-
		*2/*3	- [0]	1705.3 [1]	-
		P=	0.483	**0.035** ×	
	rs1934969	A/A	262.1 (112 – 673.9) [27]	511 (157.8 – 1088.9) [30]	0.190
		A/T	725 (191.5 – 1146.3) [15]	758.3 (238.2 – 910.8) [8]	0.897
		T/T	122.5 (60.2 – 474.2) [4]	539.2 (22.8 – 1055.6) [2]	0.643
		P=	0.344	0.870	

*P<0.05 for Mann Whitney´s U test . × P<0.05 for Kruskal Wallis test. ¥ P<0.05 for post-hoc test between genotypes.

Results.

- The results produced by the SPSS program are found in **S1.11.5 Metformin concentration according to treatment and genotype.**

S1.6 Glycated Hemoglobin (HbA1c) according to treatment and genotype.

Glycated Hemoglobin (HbA1c) according to treatment and genotype (n=103).

SLCA22A1: rs72552763 and rs622342.

SLCA22A2: rs316019.

SLC22A3: rs2076828.

ABCB1: rs1128503, rs2032582 and rs1045642.

CYP2C9: rs1799853, rs1057910 and rs1934969.

Metformin

Metformin + Glibenclamide

Descriptive analysis.

%HBA1c

Resumen de procesamiento de casos

OCT1 rs72552763	Control HbA1c		Casos: Válido N	Válido Porcentaje	Perdidos N	Perdidos Porcentaje	Total N	Total Porcentaje
GAT/GAT	Si control	CONCMET	17	73.9%	6	26.1%	23	100.0%
	No control	CONCMET	17	81.0%	4	19.0%	21	100.0%
del/GAT	Si control	CONCMET	14	77.8%	4	22.2%	18	100.0%
	No control	CONCMET	27	96.4%	1	3.6%	28	100.0%
del/del	Si control	CONCMET	6	85.7%	1	14.3%	7	100.0%
	No control	CONCMET	5	83.3%	1	16.7%	6	100.0%

Normality test.

Pruebas de normalidad

OCT1 rs72552763	Control HbA1c		Kolmogorov-Smirnov[a] Estadístico	gl	Sig.	Shapiro-Wilk Estadístico	gl	Sig.
GAT/GAT	Si control	CONCMET	.206	17	.003	.775	17	.001
	No control	CONCMET	.148	17	.200*	.915	17	.121
del/GAT	Si control	CONCMET	.313	14	.001	.698	14	.000
	No control	CONCMET	.137	27	.200*	.844	27	.001
del/del	Si control	CONCMET	.387	6	.004	.687	6	.001
	No control	CONCMET	.226	5	.200*	.875	5	.286

*. Este es un límite inferior de la significación verdadera.

a. Corrección de significación de Lilliefors

Descriptive data.

Percentiles

OCT1 rs72552763	Control HbA1c			Percentiles 5	10	25	50	75	90	95
GAT/GAT	Si control	Promedio ponderado (Definición 1)	CONCMET	62.868000	92.609000	129.834950	376.983900	819.813000	2172.083800	
		Bisagras de Tukey	CONCMET			133.472000	376.983900	763.070000		
	No control	Promedio ponderado (Definición 1)	CONCMET	45.940000	87.517600	169.083000	481.759000	1000.998000	1300.518400	
		Bisagras de Tukey	CONCMET			185.934000	481.759000	971.008000		
del/GAT	Si control	Promedio ponderado (Definición 1)	CONCMET	34.154000	34.752495	61.853000	137.112000	719.343250	3235.381000	
		Bisagras de Tukey	CONCMET			66.470000	137.112000	667.450000		
	No control	Promedio ponderado (Definición 1)	CONCMET	14.537600	25.692200	210.800900	790.618000	1055.678000	1450.158000	2676.207000
		Bisagras de Tukey	CONCMET			228.851400	790.618000	1030.222500		
del/del	Si control	Promedio ponderado (Definición 1)	CONCMET	80.594990	80.594990	100.922173	139.152950	528.638925		
		Bisagras de Tukey	CONCMET			107.697900	139.152950	264.307900		
	No control	Promedio ponderado (Definición 1)	CONCMET	22.222000	22.222000	46.232000	785.648000	1784.910000		
		Bisagras de Tukey	CONCMET			70.242000	785.648000	1642.771000		

Inferential analysis.

Inferential analysis.

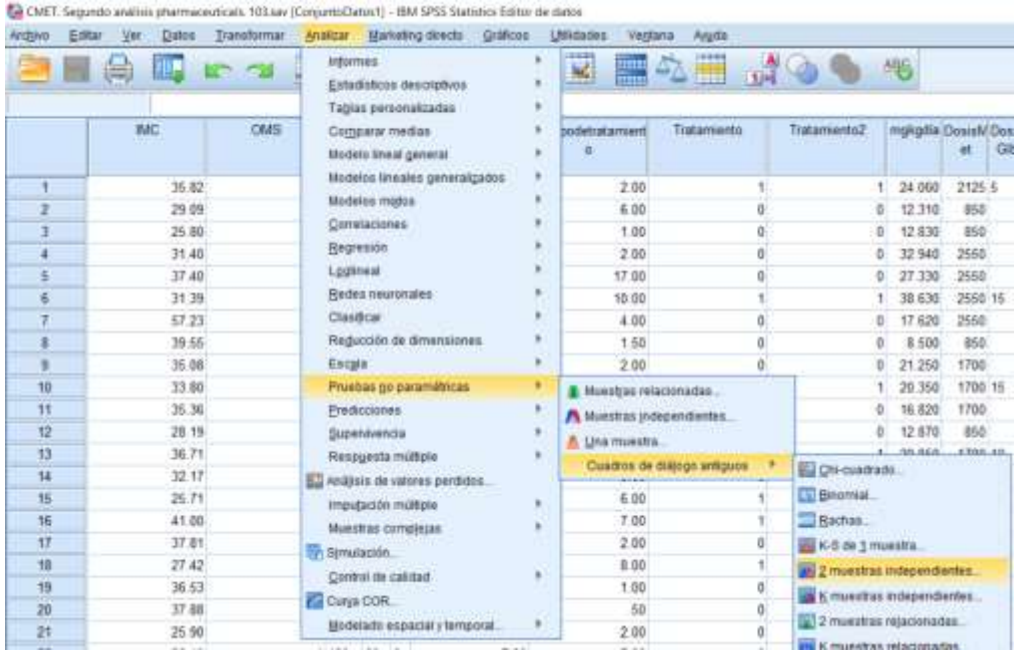

MANN WHITNEY´S U TEST.

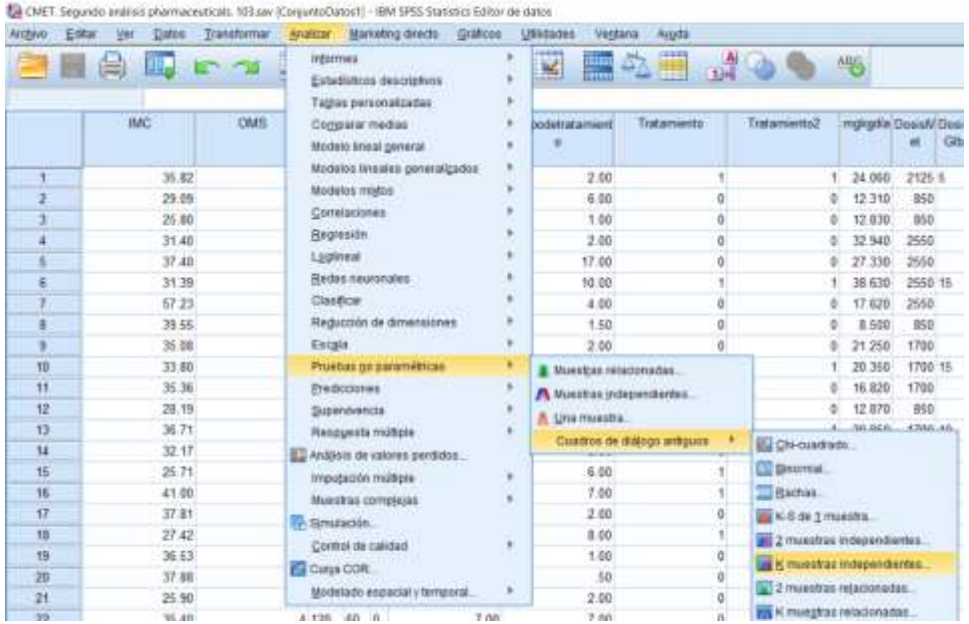

KRUSKAL WALLIS TEST.

Table 6. Glycated Hemoglobin (HbA1c) among DMT2 patients grouped by treatment and genotype.

Gene	SNP	Genotype	Metformin (%HbA1c) median (IQR 25-75%) [n]	Metformin+Glibenclamide (%HbA1c) median (IQR 25-75%) [n]	*P=
SLC22A1 (OCT1)	rs72552763	GAT/GAT	6 (5.7- 6.8) [28] ¥	9.4 (8.5 – 11.2) [16]	**<0.001***
		del/GAT	6.5 (6.2 – 9.2) [22]	8.3 (7.3 – 10.4) [24]	**0.023***
		del/del	6.5 (6.4 – 6.8) [9]	10.1 (8.4- 11.3) [4]	**0.045***
		P=	**0.022** ¤	0.221	
		A/A	6 (5.8 – 6.5) [25]	9.4 (8.5 – 11.2) [16]	**<0.001***

Results.

- The results produced by the SPSS program are found in **S1.11.6 Glycated Hemoglobin (HbA1c) according to treatment and genotype (n=103).**

S1.7 Dominant models.

1. OCT1 (rs72552763) and (rs622342)
2. ABCB1 (rs1128503) and (rs2032582)

Logistic regression on genotypic models in *ABCB1* and *OCT1* for HbA1c non-control across the whole population (n=103).

Patients with Genotype and HbA1c (n=103).

Example for **rs72552763**

Dependent variable:
Non control by HbA1c (HbA1c ≥7%)

MODEL 1

Simple dominant model.

A copy of *del* allele is sufficient for modify risk. GAT/del and del/del carriers have the same risk.

GAT/GAT vs del/GAT + del/del

MODEL 2

Multiple dominant model.

Adjusted model by covariates: Age, BMI, treatment, and treatment period.

GAT/GAT vs del/GAT + del/del

MODEL 3

Simple codominant model.

Each genotype has the same risk and not additional risk.

1. **GAT/GAT**
2. **del/GAT**
3. **del/del**

Logistic regression on genotypic models in *ABCB1* and *OCT1* for HbA1c non-control across the whole population (n=103).

Example for **rs72552763**

MODEL 1

Simple dominant model.

A copy of *del* allele is sufficient for modify risk. GAT/del and del/del carriers have the same risk.

GAT/GAT vs del/GAT + del/del

Variables en la ecuación

		B	Error estándar	Wald	gl	Sig.	Exp(B)	95% C.I. para EXP(B)	
								Inferior	Superior
Paso 1[a]	DOMOCT1rs72552763(1)	.398	.401	.989	1	.320	1.490	.679	3.266
	Constante	-.091	.302	.091	1	.763	.913		

a. Variables especificadas en el paso 1: DOMOCT1rs72552763.

Logistic regression on genotypic models in *ABCB1* and *OCT1* for HbA1c non-control across the whole population (n=103).

Example for **rs72552763**

MODEL 2

Multiple dominant model.

Adjusted model by covariables: Age, BMI, treatment, and treatment period.

GAT/GAT vs del/GAT + del/del

Variables en la ecuación

		B	Error estándar	Wald	gl	Sig.	Exp(B)	95% C.I. para EXP(B)	
								Inferior	Superior
Paso 1[a]	DOMOCT1rs72552763(1)	.188	.570	.109	1	.741	1.207	.395	3.691
	Edad	-.066	.030	5.037	1	.025	.936	.883	.992
	Tiempodetratamiento	.230	.071	10.476	1	.001	1.259	1.095	1.448
	Tratamiento2(1)	2.879	.655	19.312	1	.000	17.793	4.928	64.246
	DXIMC(1)	2.116	1.033	4.194	1	.041	8.298	1.095	62.873
	Constante	-.706	1.735	.166	1	.684	.493		

a. Variables especificadas en el paso 1: DOMOCT1rs72552763, Edad, Tiempodetratamiento, Tratamiento2, DXIMC.

Logistic regression on genotypic models in *ABCB1* and *OCT1* for HbA1c non-control across the whole population (n=103).

Example for **rs72552763**

MODEL 3

Simple codominant model.

Each genotype has the same risk and not additional risk

1. **GAT/GAT**
2. **del/GAT**
3. **del/del**

Variables en la ecuación

		B	Error estándar	Wald	gl	Sig.	Exp(B)	95% C.I. para EXP(B)	
								Inferior	Superior
Paso 1[a]	OCT1rs72552763			1.862	2	.394			
	OCT1rs72552763(1)	.533	.427	1.557	1	.212	1.704	.738	3.935
	OCT1rs72552763(2)	-.063	.633	.010	1	.920	.939	.272	3.246
	Constante	-.091	.302	.091	1	.763	.913		

a. Variables especificadas en el paso 1: OCT1rs72552763.

Results.

- The results produced by the SPSS program are found in **S1.11.7 Dominant models: *OCT1* and *ABCB1*.**

S1.8 Diplotype analysis.

1.- **OCT1 (rs72552763) and (rs622342). GAT/GAT + A/A.**

2.- ABCB1 (rs1128503) and (rs2032582). C/C + G/G.

Multiple logistic regression on HbA1c non-control among DMT2 patients.

Patients with: Genotype and HbA1c (n=103).

Adjusted model.

Independent variables:

1.- GAT.AA:
- **Reference**: GAT/GAT rs72552763 and A/A rs622342 non-carriers.
- **Odds ratio** GAT/GAT rs72552763 and A/A rs622342 carriers.

2.- AGE: Quantitative variable.

3.- Period treatment. Quantitative variable.

4.- BMI.
- **Reference**: BMI < 25 kg/m².
- **Odds ratio**: BMI ≥ 25 kg/m².

5.- Treatment.
- **Reference**: Metformin monotherapy.
- **Odds ratio**: Combined metformin and glibenclamide therapy.

Multiple logistic regression.

Dependent variable.

Non-control (HbA1c ≥7%).

Variables en la ecuación

		B	Error estándar	Wald	gl	Sig.	Exp(B)	95% C.I. para EXP(B) Inferior	Superior
Paso 1[a]	Edad	-.066	.030	4.959	1	.026	.936	.883	.992
	Tiempodetratamiento	.232	.072	10.286	1	.001	1.261	1.094	1.452
	DXIMC(1)	1.953	.967	4.079	1	.043	7.049	1.059	46.895
	GAT.AA(1)	-.642	.593	1.172	1	.279	.526	.165	1.683
	Tratamiento2(1)	2.893	.653	19.602	1	.000	18.050	5.015	64.969
	Constante	-.197	1.610	.015	1	.903	.822		

a. Variables especificadas en el paso 1: Edad, Tiempodetratamiento, DXIMC, GAT.AA, Tratamiento2.

Multiple logistic regression on HbA1c non-control among DMT2 patients.

Patients with: Genotype and HbA1c (n=103).

Adjusted model.

Independent variables:

1.- ABCB1DOM:
- **Reference**: C/C rs1128503 and G/G rs2032582 non-carriers.
- **Odds ratio:** C/C rs1128503 and G/G rs2032582 carriers.

2.- AGE: Quantitative variable.

3.- Period treatment. Quantitative variable.

4.- BMI.
- **Reference**: BMI < 25 kg/m².
- **Odds ratio**: BMI ≥ 25 kg/m².

5.- Treatment.
- **Reference**: Metformin monotherapy.
- **Odds ratio**: Combined metformin and glibenclamide therapy.

Multiple logistic regression.

Dependent variable.

Non-control (HbA1c ≥7%).

Variables en la ecuación

		B	Error estándar	Wald	gl	Sig.	Exp(B)	95% C.I. para EXP(B) Inferior	95% C.I. para EXP(B) Superior
Paso 1[a]	Edad	-.072	.030	5.709	1	.017	.930	.877	.987
	Tiempodetratamiento	.240	.072	11.228	1	.001	1.271	1.105	1.462
	Tratamiento2(1)	2.887	.666	18.764	1	.000	17.933	4.858	66.205
	ABCB1DOM(1)	-1.026	.738	1.934	1	.164	.358	.084	1.522
	DXIMC(1)	2.211	1.088	4.132	1	.042	9.128	1.083	76.966
	Constante	-.239	1.679	.020	1	.887	.788		

a. Variables especificadas en el paso 1: Edad, Tiempodetratamiento, Tratamiento2, ABCB1DOM, DXIMC.

Figure S1.8.1 Forest Plot graphics showing OR by covariates influencing patient's control/no-control. Upper panel: rs72552763 (*GAT/GAT*) and rs622342 (*A/A*) in *OCT1*. Lower panel: rs1128503 (*C/C*) and rs2032582 (*G/G*) in *ABCB1*.

Study or Subgroup	log[Odds Ratio]	SE	Odds Ratio IV, Fixed, 95% CI
Age	-0.066	0.03	0.94 [0.88, 0.99]
BMI ≥25 Kg/m²	1.953	0.967	7.05 [1.06, 46.91]
rs72552763 (GAT/GAT) and rs622342 (A/A)	-0.642	0.593	0.53 [0.16, 1.68]
Treatment (Metformin + Glibenclamide)	2.893	0.653	18.05 [5.02, 64.90]
Treatment period	0.232	0.072	1.26 [1.10, 1.45]

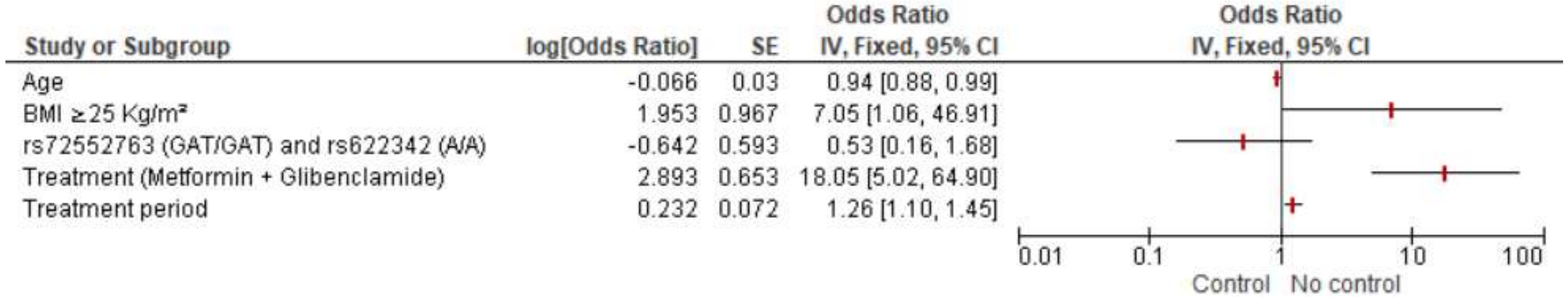

Study or Subgroup	log[Odds Ratio]	SE	Odds Ratio IV, Fixed, 95% CI
Age	-0.072	0.03	0.93 [0.88, 0.99]
BMI ≥25 kg/m²	2.211	1.088	9.12 [1.08, 76.97]
rs1128503 (C/C) and rs2032582 (G/G)	-1.026	0.738	0.36 [0.08, 1.52]
Treatment (Metformin + Glibenclamide)	2.887	0.666	17.94 [4.86, 66.18]
Treatment period	0.24	0.072	1.27 [1.10, 1.46]

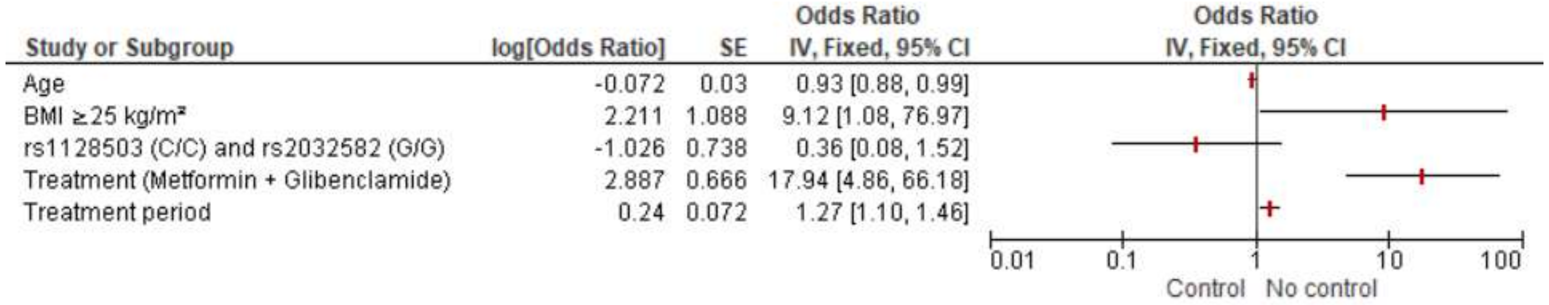

S1.9 Metformin dose and genotype.

Group by genotype in ABCB1.

Daily metformin dose mg/kg/day among DMT2 patients according to treatment and genotype).

ABCB1: rs1128503, rs2032582 and rs1045642.

Metformin

Metformin + Glibenclamide

Metformin dose (mg/kg/day).

Descriptive analysis.

Resumen de procesamiento de casos

	ABCB1 rs1128503	Casos					
		Válido		Perdidos		Total	
		N	Porcentaje	N	Porcentaje	N	Porcentaje
mgkgdía	CC	14	93.3%	1	6.7%	15	100.0%
	CT	27	96.4%	1	3.6%	28	100.0%
	TT	15	93.8%	1	6.3%	16	100.0%

Normality test.

Pruebas de normalidad

	ABCB1 rs1128503	Kolmogorov-Smirnov[a]			Shapiro-Wilk		
		Estadístico	gl	Sig.	Estadístico	gl	Sig.
mgkgdía	CC	.139	14	.200*	.944	14	.476
	CT	.151	27	.119	.915	27	.030
	TT	.197	15	.120	.915	15	.164

*. Esto es un límite inferior de la significación verdadera.

a. Corrección de significación de Lilliefors

Descriptive data.

Percentiles

		ABCB1 rs1128503	Percentiles						
			5	10	25	50	75	90	95
Promedio ponderado (Definición 1)	mgkgdía	CC	8.5000	9.3450	12.0775	18.7850	25.6075	35.4400	.
		CT	9.8320	9.8800	11.9200	19.1200	26.6800	30.3200	37.1760
		TT	7.7200	8.5600	10.5800	20.0000	27.4600	38.9900	.
Bisagras de Tukey	mgkgdía	CC			12.2800	18.7850	25.4100		
		CT			11.9250	19.1200	26.0800		
		TT			11.7850	20.0000	24.4200		

Inferential analysis.

Inferential analysis.

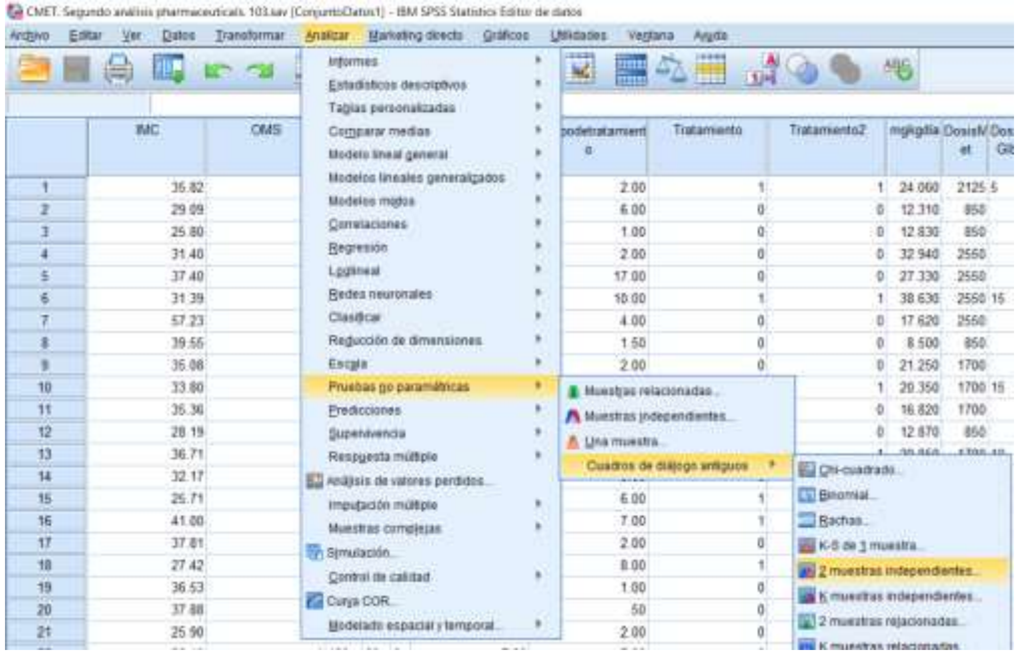

MANN WHITNEY´S U TEST.

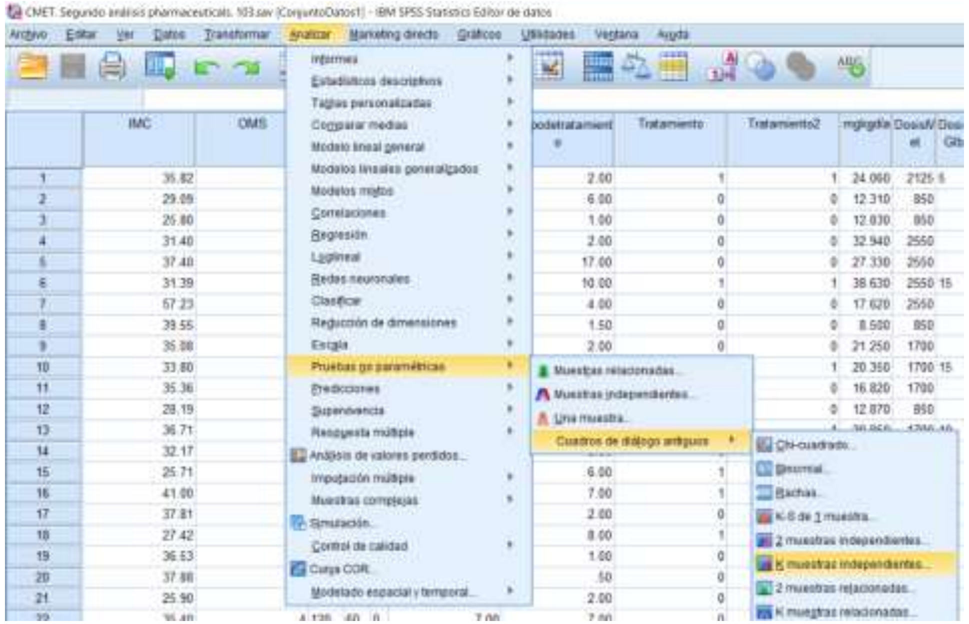

KRUSKAL WALLIS TEST.

Table 9. Daily metformin dose mg/kg/day among DMT2 patients grouped by treatment and genotype.

Gene	SNP	Genotype	Metformin (mg/kg/day) median (IQR 25-75%) [n]	Metformin+Glibenclamide (mg/kg/day) median (IQR 25-75%) [n]	P=
		C/C	18.78 (12.28 – 25.41) [14]	26.98 (18.19 – 34.53) [12]	0.057
		C/T	19.12 (11.92 – 26.08) [27]	29.27 (23.16 – 35.31) [17]	**0.001***
	rs1128503	T/T	20 (11.78 – 24.42) [15]	29.7 (16.66 – 31.95) [14]	0.127
		P=	0.969	0.971	
		G/G	16.48 (11.93 – 23.28) [17]	26.98 (21.35 – 32.04) [15]	**0.011***

Results.

- The results produced by the SPSS program are found in **S1.11.8 Daily metformin dose mg/kg/day among DMT2 according to treatment and genotype).**

S1.10 Figure: Linear regression.

Metformin concentration (mg/dl) dose and metformin dose (mg/kg/day).

Linear regression (metformin group).

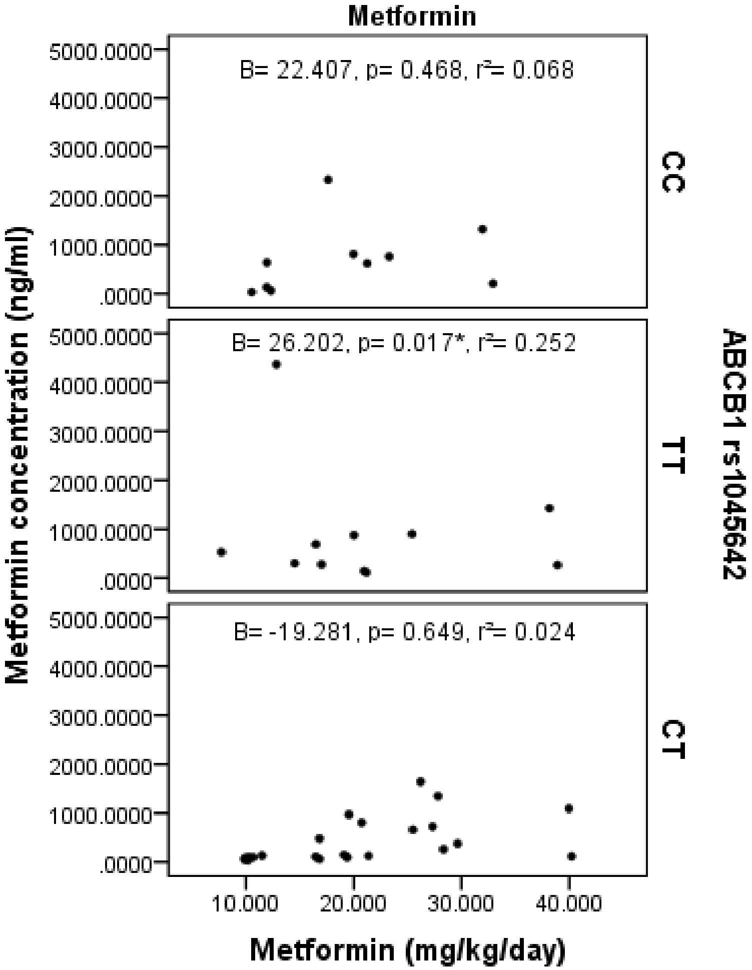

ABCB1 rs1045642 CC.

Resumen del modelo[a]

Modelo	R	R cuadrado	R cuadrado ajustado	Error estándar de la estimación	Estadísticos de cambio				
					Cambio en R cuadrado	Cambio en F	gl1	gl2	Sig. Cambio en F
1	.260[b]	.068	-.049	722.061560	.068	.581	1	8	.468

a. ABCB1 rs1045642 = CC

b. Predictores: (Constante), mgkgdía

Coeficientes[a,b]

Modelo		Coeficientes no estandarizados		Coeficientes estandarizados	t	Sig.	95.0% intervalo de confianza para B	
		B	Error estándar	Beta			Límite inferior	Límite superior
1	(Constante)	259.189	613.477		.422	.684	-1155.492	1673.870
	mgkgdía	22.407	29.398	.260	.762	.468	-45.384	90.198

a. ABCB1 rs1045642 = CC

b. Variable dependiente: CONCMET

Linear regression (metformin group).

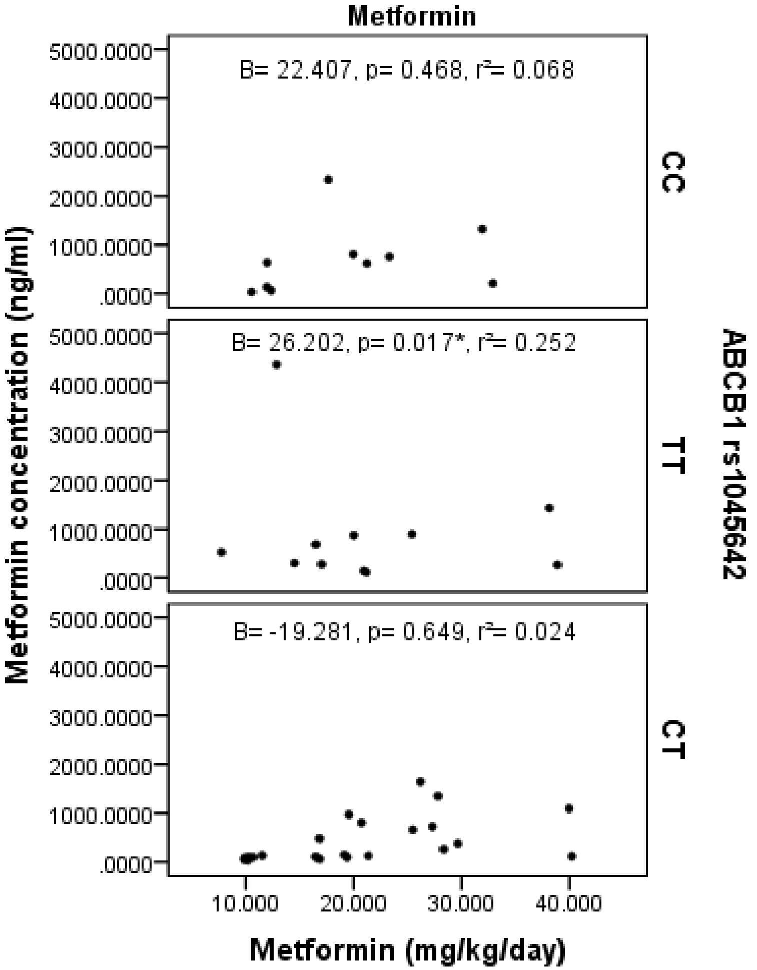

ABCB1 rs1045642 CT.

Resumen del modelo[a]

Modelo	R	R cuadrado	R cuadrado ajustado	Error estándar de la estimación	Estadísticos de cambio				
					Cambio en R cuadrado	Cambio en F	gl1	gl2	Sig. Cambio en F
1	.502[b]	.252	.215	419.705037	.252	6.747	1	20	.017

a. ABCB1 rs1045642 = CT

b. Predictores: (Constante), mgkgdía

Coeficientes[a,b]

Modelo		Coeficientes no estandarizados		Coeficientes estandarizados	t	Sig.	95.0% intervalo de confianza para B	
		B	Error estándar	Beta			Límite inferior	Límite superior
1	(Constante)	-111.972	228.126		-.491	.629	-587.835	363.890
	mgkgdía	26.202	10.087	.502	2.597	.017	5.160	47.244

a. ABCB1 rs1045642 = CT

b. Variable dependiente: CONCMET

Linear regression (metformin group).

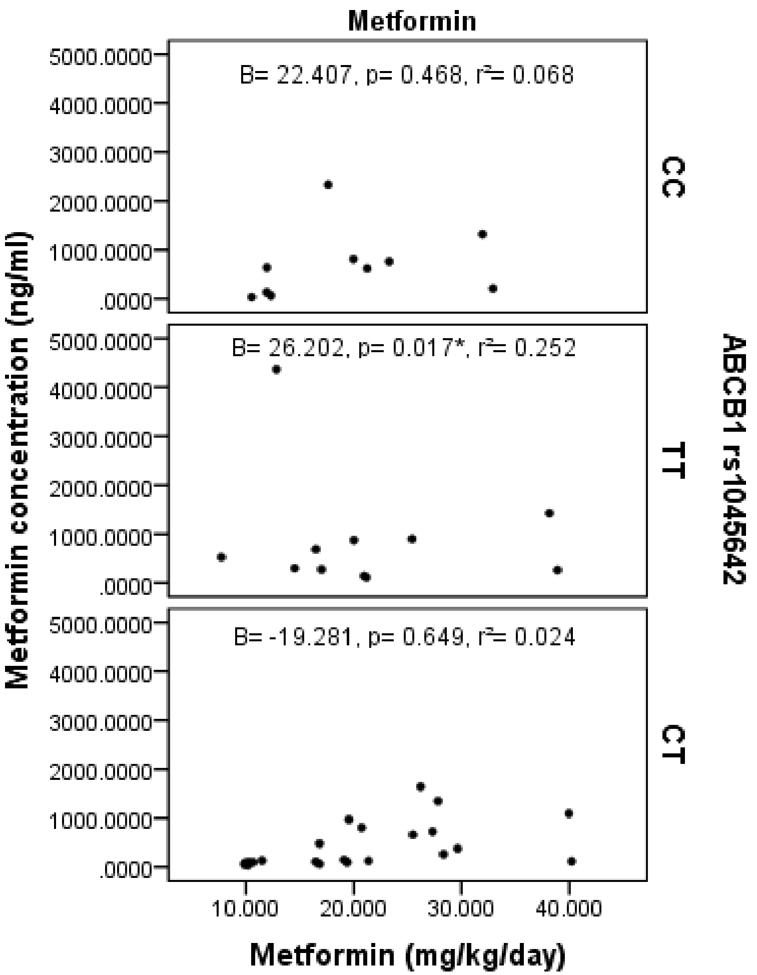

ABCB1 rs1045642 TT.

Resumen del modelo[a]

Modelo	R	R cuadrado	R cuadrado ajustado	Error estándar de la estimación	Estadísticos de cambio				
					Cambio en R cuadrado	Cambio en F	gl1	gl2	Sig. Cambio en F
1	.155[b]	.024	-.084	1268.315849	.024	.221	1	9	.649

a. ABCB1 rs1045642 = TT

b. Predictores: (Constante), mgkgdía

Coeficientes[a,b]

Modelo		Coeficientes no estandarizados		Coeficientes estandarizados	t	Sig.	95.0% intervalo de confianza para B	
		B	Error estándar	Beta			Límite inferior	Límite superior
1	(Constante)	1307.564	949.077		1.378	.202	-839.398	3454.526
	mgkgdía	-19.281	40.975	-.155	-.471	.649	-111.972	73.410

a. ABCB1 rs1045642 = TT

b. Variable dependiente: CONCMET

Linear regression (combined therapy).

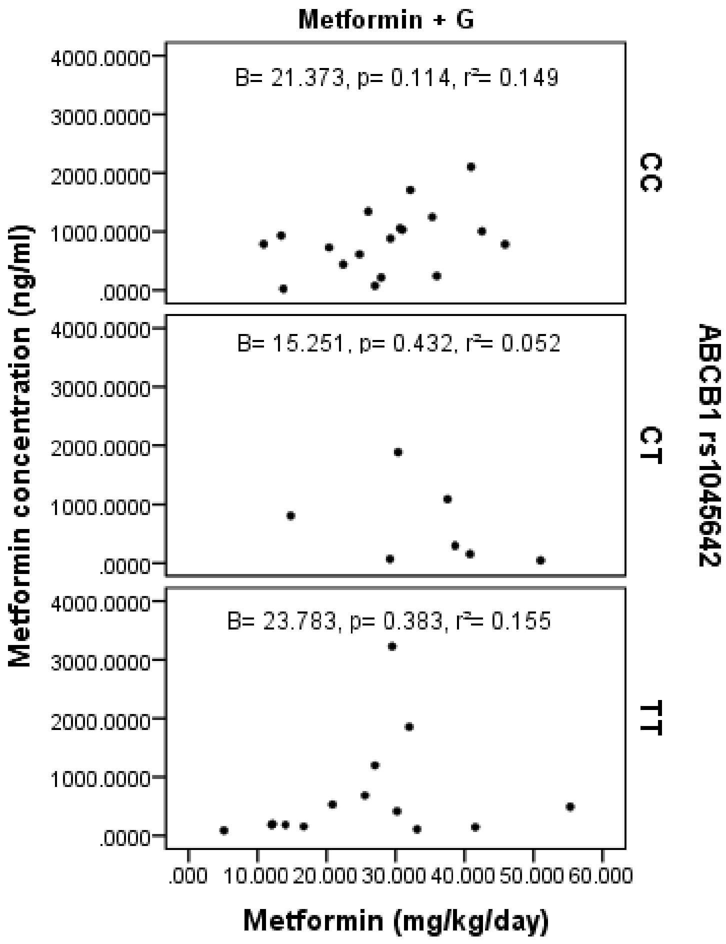

ABCB1 rs1045642 CC.

Resumen del modelo[a]

Modelo	R	R cuadrado	R cuadrado ajustado	Error estándar de la estimación	Estadísticos de cambio				
					Cambio en R cuadrado	Cambio en F	gl1	gl2	Sig. Cambio en F
1	.386[b]	.149	.096	520.830157	.149	2.802	1	16	.114

a. ABCB1 rs1045642 = CC

b. Predictores: (Constante), mgkgdía

Coeficientes[a,b]

Modelo		Coeficientes no estandarizados		Coeficientes estandarizados	t	Sig.	95.0% intervalo de confianza para B	
		B	Error estándar	Beta			Límite inferior	Límite superior
1	(Constante)	240.948	381.944		.631	.537	-568.737	1050.632
	mgkgdía	21.373	12.769	.386	1.674	.114	-5.696	48.443

a. ABCB1 rs1045642 = CC

b. Variable dependiente: Metformin concentration (ng/ml)

Linear regression (combined therapy).

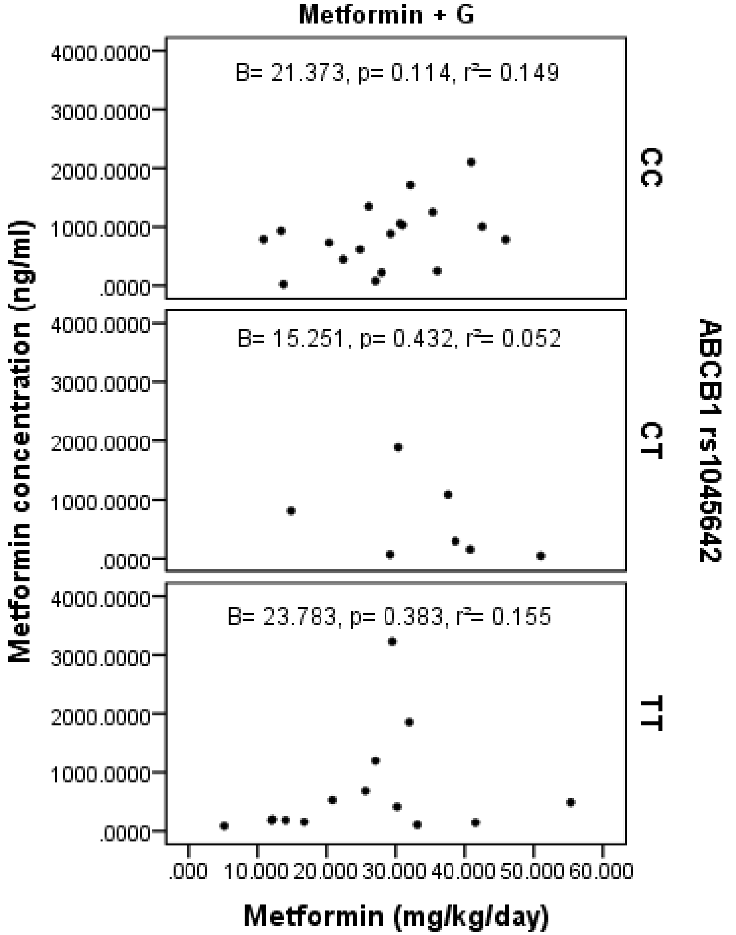

ABCB1 rs1045642 CT.

Resumen del modelo[a]

Modelo	R	R cuadrado	R cuadrado ajustado	Error estándar de la estimación	Estadísticos de cambio				
					Cambio en R cuadrado	Cambio en F	gl1	gl2	Sig. Cambio en F
1	.229[b]	.052	-.027	897.778080	.052	.662	1	12	.432

a. ABCB1 rs1045642 = CT

b. Predictores: (Constante), mgkgdía

Coeficientes[a,b]

Modelo		Coeficientes no estandarizados		Coeficientes estandarizados	t	Sig.	95.0% intervalo de confianza para B	
		B	Error estándar	Beta			Límite inferior	Límite superior
1	(Constante)	289.817	532.415		.544	.596	-870.215	1449.849
	mgkgdía	15.251	18.747	.229	.814	.432	-25.595	56.098

a. ABCB1 rs1045642 = CT

b. Variable dependiente: Metformin concentration (ng/ml)

Linear regression (combined therapy).

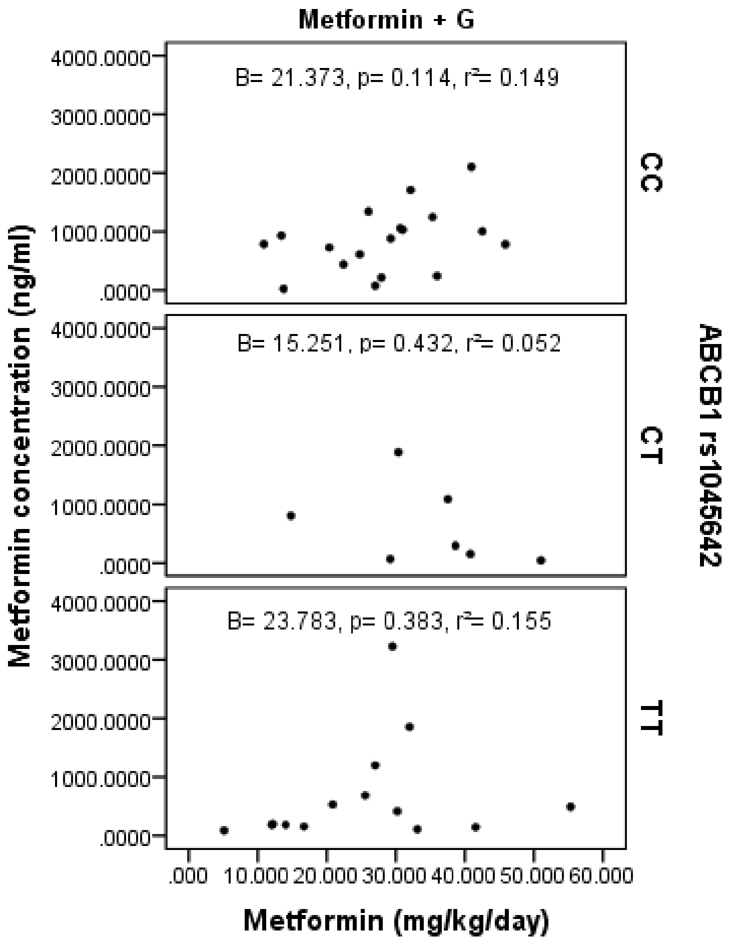

ABCB1 rs1045642 TT.

Resumen del modelo[a]

Modelo	R	R cuadrado	R cuadrado ajustado	Error estándar de la estimación	Estadísticos de cambio				
					Cambio en R cuadrado	Cambio en F	gl1	gl2	Sig. Cambio en F
1	.393[b]	.155	-.014	690.249795	.155	.916	1	5	.383

a. ABCB1 rs1045642 = TT

b. Predictores: (Constante), mgkgdía

Coeficientes[a,b]

Modelo		Coeficientes no estandarizados		Coeficientes estandarizados	t	Sig.	95.0% intervalo de confianza para B	
		B	Error estándar	Beta			Límite inferior	Límite superior
1	(Constante)	1443.022	898.914		1.605	.169	-867.709	3753.753
	mgkgdía	-23.783	24.854	-.393	-.957	.383	-87.672	40.105

a. ABCB1 rs1045642 = TT

b. Variable dependiente: Metformin concentration (ng/ml)

S1.11 Statistical results and procedures.

S1.11.1 Table 1.

S1.11.2 Hardy Weinberg Equilibrium.

S1.11.3 Table 3.

S1.11.4 Metformin plasmatic concentrations among DMT2 patients according to SNP and genotype.

S1.11.5 Metformin concentration according to treatment and genotype.

S1.11.6 Glycated Hemoglobin (HbA1c) according to treatment and genotype (n=103).

S1.11.7 Dominant models: *OCT1* and *ABCB1*.

S1.11.8 Daily metformin dose mg/kg/day among DMT2 according to treatment and genotype).

S1.11.1 Table 1

AGE

DESCRIPTIVE DATA.

AGE BY TREATMENT

Resumen de procesamiento de casos

	Tratamiento2	Casos					
		Válido		Perdidos		Total	
		N	Porcentaje	N	Porcentaje	N	Porcentaje
Edad	Metformina	59	100.0%	0	0.0%	59	100.0%
	Met + Glb	44	100.0%	0	0.0%	44	100.0%

Descriptivos

	Tratamiento2			Estadístico	Error estándar
Edad	Metformina	Media		55.80	1.411
		95% de intervalo de confianza para la media	Límite inferior	52.97	
			Límite superior	58.62	
		Media recortada al 5%		55.68	
		Mediana		56.00	
		Varianza		117.406	
		Desviación estándar		10.835	
		Mínimo		29	
		Máximo		86	
		Rango		57	
		Rango intercuartil		14	
		Asimetría		.199	.311
		Curtosis		.561	.613
	Met + Glb	Media		53.11	1.701
		95% de intervalo de confianza para la media	Límite inferior	49.68	
			Límite superior	56.54	
		Media recortada al 5%		52.94	
		Mediana		51.00	
		Varianza		127.359	
		Desviación estándar		11.285	
		Mínimo		30	
		Máximo		76	
		Rango		46	
		Rango intercuartil		17	
		Asimetría		.332	.357
		Curtosis		-.557	.702

NORMALITY TEST.

Pruebas de normalidad

	Tratamiento2	Kolmogorov-Smirnov[a]			Shapiro-Wilk		
		Estadístico	gl	Sig.	Estadístico	gl	Sig.
Edad	Metformina	.069	59	.200*	.990	59	.894
	Met + Glb	.097	44	.200*	.973	44	.390

*. Esto es un límite inferior de la significación verdadera.

a. Corrección de significación de Lilliefors

T STUDENT AND LEVENE TEST.

Prueba de muestras independientes

		Prueba de Levene de igualdad de varianzas		prueba t para la igualdad de medias						
									95% de intervalo de confianza de la diferencia	
		F	Sig.	t	gl	Sig. (bilateral)	Diferencia de medias	Diferencia de error estándar	Inferior	Superior
Edad	Se asumen varianzas iguales	.538	.465	1.221	101	.225	2.683	2.197	-1.675	7.041
	No se asumen varianzas iguales			1.214	90.674	.228	2.683	2.210	-1.707	7.073

WEIGHT.

DESCRIPTIVE DATA.

Descriptivos

	Tratamiento2			Estadístico	Error estándar
Peso	Metformina	Media		78.834	2.1782
		95% de intervalo de confianza para la media	Límite inferior	74.474	
			Límite superior	83.194	
		Media recortada al 5%		78.098	
		Mediana		80.000	
		Varianza		279.916	
		Desviación estándar		16.7307	
		Mínimo		50.0	
		Máximo		144.7	
		Rango		94.7	
		Rango intercuartil		20.5	
		Asimetría		1.004	.311
		Curtosis		2.825	.613
	Met + Glb	Media		71.720	2.0197
		95% de intervalo de confianza para la media	Límite inferior	67.647	
			Límite superior	75.794	
		Media recortada al 5%		71.517	
		Mediana		71.000	
		Varianza		179.491	
		Desviación estándar		13.3974	
		Mínimo		46.1	
		Máximo		99.8	
		Rango		53.7	
		Rango intercuartil		20.5	
		Asimetría		.257	.357
		Curtosis		-.560	.702

NORMALITY TEST.

Pruebas de normalidad

		Kolmogorov-Smirnov[a]			Shapiro-Wilk		
	Tratamiento2	Estadístico	gl	Sig.	Estadístico	gl	Sig.
Peso	Metformina	.097	59	.200*	.940	59	.006
	Met + Glb	.091	44	.200*	.979	44	.611

*. Esto es un límite inferior de la significación verdadera.

a. Corrección de significación de Lilliefors

T STUDENT AND LEVENE TEST.

Prueba de muestras independientes

		Prueba de Levene de igualdad de varianzas		prueba t para la igualdad de medias						
									95% de intervalo de confianza de la diferencia	
		F	Sig.	t	gl	Sig. (bilateral)	Diferencia de medias	Diferencia de error estándar	Inferior	Superior
Peso	Se asumen varianzas iguales	.882	.350	2.319	101	.022	7.1134	3.0675	1.0283	13.1986
	No se asumen varianzas iguales			2.395	100.450	.018	7.1134	2.9705	1.2204	13.0064

SEX

FREQUENCY TABLE WITH OBSERVED AND EXPECTED COUNTS AND PERCENTAGE.

Tabla cruzada Sexo*Tratamiento2

			Tratamiento2		Total
			Metformina	Met + Glb	
Sexo	Mujer	Recuento	42	31	73
		Recuento esperado	41.8	31.2	73.0
		% dentro de Tratamiento2	71.2%	70.5%	70.9%
	Hombre	Recuento	17	13	30
		Recuento esperado	17.2	12.8	30.0
		% dentro de Tratamiento2	28.8%	29.5%	29.1%
Total		Recuento	59	44	103
		Recuento esperado	59.0	44.0	103.0
		% dentro de Tratamiento2	100.0%	100.0%	100.0%

STATISTICAL INFERENCE TEST: PEARSON´S CHI SQUARE TEST.

Pruebas de chi-cuadrado

	Valor	gl	Significación asintótica (bilateral)	Significación exacta (bilateral)	Significación exacta (unilateral)
Chi-cuadrado de Pearson	.007[a]	1	.936		
Corrección de continuidad[b]	.000	1	1.000		
Razón de verosimilitud	.007	1	.936		
Prueba exacta de Fisher				1.000	.553
Asociación lineal por lineal	.006	1	.936		
N de casos válidos	103				

a. 0 casillas (0.0%) han esperado un recuento menor que 5. El recuento mínimo esperado es 12.82.

b. Sólo se ha calculado para una tabla 2x2

TREATMENT PERIOD

DESCRIPTIVE DATA.

Descriptivos

	Tratamiento2			Estadístico	Error estándar
Tiempo de tratamiento	Metformina	Media		5.0705	.61993
		95% de intervalo de confianza para la media	Límite inferior	3.8296	
			Límite superior	6.3114	
		Media recortada al 5%		4.5927	
		Mediana		3.0000	
		Varianza		22.675	
		Desviación estándar		4.76180	
		Mínimo		.16	
		Máximo		21.00	
		Rango		20.84	
		Rango intercuartil		7.00	
		Asimetría		1.399	.311
		Curtosis		1.651	.613
	Met + Glb	Media		9.3409	1.10970
		95% de intervalo de confianza para la media	Límite inferior	7.1030	
			Límite superior	11.5788	
		Media recortada al 5%		8.7525	
		Mediana		7.5000	
		Varianza		54.183	
		Desviación estándar		7.36094	
		Mínimo		1.00	
		Máximo		36.00	
		Rango		35.00	
		Rango intercuartil		11.75	
		Asimetría		1.347	.357
		Curtosis		2.437	.702

NORMALITY TEST.

Pruebas de normalidad

		Kolmogorov-Smirnov[a]			Shapiro-Wilk		
	Tratamiento2	Estadístico	gl	Sig.	Estadístico	gl	Sig.
Tiempo de tratamiento	Metformina	.199	59	.000	.840	59	.000
	Met + Glb	.152	44	.012	.876	44	.000

a. Corrección de significación de Lilliefors

MEDIAN AND QUARTILE RANGE.

Percentiles

			Percentiles						
		Tratamiento2	5	10	25	50	75	90	95
Promedio ponderado (Definición 1)	Tiempo de tratamiento	Metformina	.5000	1.0000	1.0000	3.0000	8.0000	12.0000	17.0000
		Met + Glb	1.2500	2.0000	3.0000	7.5000	14.7500	20.0000	21.5000
Bisagras de Tukey	Tiempo de tratamiento	Metformina			1.2500	3.0000	7.5000		
		Met + Glb			3.0000	7.5000	14.5000		

MANN WHITNEY´S U TEST.

Estadísticos de prueba[a]

	Tiempo de tratamiento
U de Mann-Whitney	773.500
W de Wilcoxon	2543.500
Z	-3.509
Sig. asintótica (bilateral)	.000

a. Variable de agrupación: Tratamiento2

BMI Kg/m^2.

DESCRIPTIVE DATA.

Descriptivos

	Tratamiento2			Estadístico	Error estándar
IMC	Metformina	Media		31.9215	.82010
		95% de intervalo de confianza para la media	Límite inferior	30.2799	
			Límite superior	33.5631	
		Media recortada al 5%		31.4956	
		Mediana		31.0000	
		Varianza		39.681	
		Desviación estándar		6.29928	
		Mínimo		22.22	
		Máximo		57.23	
		Rango		35.01	
		Rango intercuartil		8.51	
		Asimetría		1.319	.311
		Curtosis		3.136	.613
	Met + Glb	Media		29.2364	.72657
		95% de intervalo de confianza para la media	Límite inferior	27.7711	
			Límite superior	30.7016	
		Media recortada al 5%		29.1855	
		Mediana		28.3850	
		Varianza		23.228	
		Desviación estándar		4.81952	
		Mínimo		19.95	
		Máximo		41.00	
		Rango		21.05	
		Rango intercuartil		5.97	
		Asimetría		.309	.357
		Curtosis		-.184	.702

NORMALITY TEST.

Pruebas de normalidad

		Kolmogorov-Smirnov[a]			Shapiro-Wilk		
	Tratamiento2	Estadístico	gl	Sig.	Estadístico	gl	Sig.
IMC	Metformina	.114	59	.056	.914	59	.000
	Met + Glb	.086	44	.200*	.980	44	.626

*. Esto es un límite inferior de la significación verdadera.

a. Corrección de significación de Lilliefors

T STUDENT AND LEVENE TEST.

Prueba de muestras independientes

		Prueba de Levene de igualdad de varianzas		prueba t para la igualdad de medias						
									95% de intervalo de confianza de la diferencia	
		F	Sig.	t	gl	Sig. (bilateral)	Diferencia de medias	Diferencia de error estándar	Inferior	Superior
IMC	Se asumen varianzas iguales	1.973	.163	2.358	101	.020	2.68516	1.13863	.42643	4.94389
	No se asumen varianzas iguales			2.451	100.919	.016	2.68516	1.09566	.51165	4.85867

BMI BY WHO´S CLASIFICATION.

DESCRIPTIVE DATA.

Tabla cruzada OMS*Tratamiento2

			Tratamiento2		Total
			Metformina	Met + Glb	
OMS	Normopeso	Recuento	4	7	11
		Recuento esperado	6.3	4.7	11.0
		% dentro de Tratamiento2	6.8%	15.9%	10.7%
	Sobrepeso	Recuento	20	19	39
		Recuento esperado	22.3	16.7	39.0
		% dentro de Tratamiento2	33.9%	43.2%	37.9%
	Obesidad	Recuento	18	8	26
		Recuento esperado	14.9	11.1	26.0
		% dentro de Tratamiento2	30.5%	18.2%	25.2%
	Obesidad II	Recuento	12	8	20
		Recuento esperado	11.5	8.5	20.0
		% dentro de Tratamiento2	20.3%	18.2%	19.4%
	Obesidad III	Recuento	5	2	7
		Recuento esperado	4.0	3.0	7.0
		% dentro de Tratamiento2	8.5%	4.5%	6.8%
Total		Recuento	59	44	103
		Recuento esperado	59.0	44.0	103.0
		% dentro de Tratamiento2	100.0%	100.0%	100.0%

ORDINAL VARIABLE: MANN WHITNEY´S U TEST.

Estadísticos de prueba[a]

	OMS
U de Mann-Whitney	1041.000
W de Wilcoxon	2031.000
Z	-1.785
Sig. asintótica (bilateral)	.074

a. Variable de agrupación: Tratamiento2

METFORMIN CONCENTRATION.

DESCRIPTIVE DATA.

Descriptivos

	Tratamiento2			Estadístico	Error estándar
CONCMET	Metformina	Media		619.895954	117.7700245
		95% de intervalo de confianza para la media	Límite inferior	382.694948	
			Límite superior	857.096959	
		Media recortada al 5%		510.435410	
		Mediana		290.755950	
		Varianza		638009.819	
		Desviación estándar		798.7551683	
		Mínimo		9.0180	
		Máximo		4368.1540	
		Rango		4359.1360	
		Rango intercuartil		723.3116	
		Asimetría		2.758	.350
		Curtosis		10.198	.688
	Met + Glb	Media		726.637630	110.3413426
		95% de intervalo de confianza para la media	Límite inferior	503.451198	
			Límite superior	949.824061	
		Media recortada al 5%		658.185033	
		Mediana		570.913450	
		Varianza		487008.475	
		Desviación estándar		697.8599251	
		Mínimo		22.2220	
		Máximo		3223.2370	
		Rango		3201.0150	
		Rango intercuartil		885.2825	
		Asimetría		1.527	.374
		Curtosis		2.964	.733

NORMALITY TEST.

Pruebas de normalidad

		Kolmogorov-Smirnov[a]			Shapiro-Wilk		
	Tratamiento2	Estadístico	gl	Sig.	Estadístico	gl	Sig.
CONCMET	Metformina	.222	46	.000	.705	46	.000
	Met + Glb	.156	40	.015	.854	40	.000

a. Corrección de significación de Lilliefors

MEDIAN AND QUARTILE RANGE.

Percentiles

			Percentiles						
		Tratamiento2	5	10	25	50	75	90	95
Promedio ponderado (Definición 1)	CONCMET	Metformina	29.121050	44.206697	107.094675	290.755950	830.406250	1492.056800	2260.654600
		Met + Glb	23.973150	70.779899	164.223000	570.913450	1049.505500	1840.634000	2091.792050
Bisagras de Tukey	CONCMET	Metformina			107.697900	290.755950	815.023000		
		Met + Glb			170.589000	570.913450	1043.333000		

MANN WHITNEY´S U TEST.

Estadísticos de prueba[a]

	CONCMET
U de Mann-Whitney	773.000
W de Wilcoxon	1854.000
Z	-1.273
Sig. asintótica (bilateral)	.203

a. Variable de agrupación: Tratamiento2

GLUCOSE

DESCRIPTIVE DATA.

Descriptivos

	Tratamiento2			Estadístico	Error estándar
Glucosa	Metformina	Media		140.50	7.731
		95% de intervalo de confianza para la media	Límite inferior	125.02	
			Límite superior	155.98	
		Media recortada al 5%		134.82	
		Mediana		115.00	
		Varianza		3466.991	
		Desviación estándar		58.881	
		Mínimo		80	
		Máximo		324	
		Rango		244	
		Rango intercuartil		57	
		Asimetría		1.488	.314
		Curtosis		1.346	.618
	Met + Glb	Media		199.70	10.734
		95% de intervalo de confianza para la media	Límite inferior	178.06	
			Límite superior	221.35	
		Media recortada al 5%		195.61	
		Mediana		192.50	
		Varianza		5069.236	
		Desviación estándar		71.199	
		Mínimo		89	
		Máximo		410	
		Rango		321	
		Rango intercuartil		101	
		Asimetría		.776	.357
		Curtosis		.532	.702

NORMALITY TEST.

Pruebas de normalidad

		Kolmogorov-Smirnov[a]			Shapiro-Wilk		
	Tratamiento2	Estadístico	gl	Sig.	Estadístico	gl	Sig.
Glucosa	Metformina	.244	58	.000	.787	58	.000
	Met + Glb	.098	44	.200*	.952	44	.066

*. Esto es un límite inferior de la significación verdadera.

a. Corrección de significación de Lilliefors

MEDIAN AND QUARTILE RANGE.

Percentiles

			Percentiles						
		Tratamiento2	5	10	25	50	75	90	95
Promedio ponderado (Definición 1)	Glucosa	Metformina	88.75	95.90	101.50	115.00	158.25	230.60	269.70
		Met + Glb	103.25	121.50	138.00	192.50	238.75	294.00	346.50
Bisagras de Tukey	Glucosa	Metformina			102.00	115.00	157.00		
		Met + Glb			139.00	192.50	238.50		

MANN WHITNEY´S U TEST.

Estadísticos de prueba[a]

	Glucosa
U de Mann-Whitney	576.500
W de Wilcoxon	2287.500
Z	-4.727
Sig. asintótica (bilateral)	.000

a. Variable de agrupación: Tratamiento2

HbA1C.

DESCRIPTIVE DATA.

Descriptivos

	Tratamiento2			Estadístico	Error estándar
HbA1c	Metformina	Media		7.102	.2501
		95% de intervalo de confianza para la media	Límite inferior	6.601	
			Límite superior	7.602	
		Media recortada al 5%		6.899	
		Mediana		6.400	
		Varianza		3.690	
		Desviación estándar		1.9209	
		Mínimo		5.3	
		Máximo		14.9	
		Rango		9.6	
		Rango intercuartil		1.4	
		Asimetría		1.944	.311
		Curtosis		4.027	.613
	Met + Glb	Media		9.284	.3236
		95% de intervalo de confianza para la media	Límite inferior	8.631	
			Límite superior	9.937	
		Media recortada al 5%		9.220	
		Mediana		9.050	
		Varianza		4.607	
		Desviación estándar		2.1465	
		Mínimo		5.3	
		Máximo		15.7	
		Rango		10.4	
		Rango intercuartil		3.4	
		Asimetría		.548	.357
		Curtosis		.440	.702

NORMALITY TEST.

Pruebas de normalidad

		Kolmogorov-Smirnov[a]			Shapiro-Wilk		
	Tratamiento2	Estadístico	gl	Sig.	Estadístico	gl	Sig.
HbA1c	Metformina	.264	59	.000	.760	59	.000
	Met + Glb	.096	44	.200*	.972	44	.345

*. Esto es un límite inferior de la significación verdadera.

a. Corrección de significación de Lilliefors

MEDIAN AND QUARTILE RANGE.

Percentiles

			Percentiles						
		Tratamiento2	5	10	25	50	75	90	95
Promedio ponderado (Definición 1)	HbA1c	Metformina	5.400	5.600	5.900	6.400	7.300	10.300	11.100
		Met + Glb	5.900	6.600	7.525	9.050	10.900	12.050	12.725
Bisagras de Tukey	HbA1c	Metformina			5.950	6.400	7.300		
		Met + Glb			7.550	9.050	10.900		

MANN WHITNEY´S U TEST.

Estadísticos de prueba[a]

	HbA1c
U de Mann-Whitney	495.500
W de Wilcoxon	2265.500
Z	-5.352
Sig. asintótica (bilateral)	.000

a. Variable de agrupación: Tratamiento2

CONTROL VS NO CONTROL BY HBA1C.

FREQUENCY TABLE WITH OBSERVED AND EXPECTED COUNTS AND PERCENTAGE.

Tabla cruzada Control HbA1c*Tratamiento2

			Tratamiento2		
			Metformina	Met + Glb	Total
Control HbA1c	Si control	Recuento	43	5	48
		Recuento esperado	27.5	20.5	48.0
		% dentro de Tratamiento2	72.9%	11.4%	46.6%
	No control	Recuento	16	39	55
		Recuento esperado	31.5	23.5	55.0
		% dentro de Tratamiento2	27.1%	88.6%	53.4%
Total		Recuento	59	44	103
		Recuento esperado	59.0	44.0	103.0
		% dentro de Tratamiento2	100.0%	100.0%	100.0%

STATISTICAL INFERENCE TEST: PEARSON´S CHI SQUARE TEST.

Pruebas de chi-cuadrado

	Valor	gl	Significación asintótica (bilateral)	Significación exacta (bilateral)	Significación exacta (unilateral)
Chi-cuadrado de Pearson	38.330[a]	1	.000		
Corrección de continuidad[b]	35.898	1	.000		
Razón de verosimilitud	42.192	1	.000		
Prueba exacta de Fisher				.000	.000
Asociación lineal por lineal	37.958	1	.000		
N de casos válidos	103				

a. 0 casillas (0.0%) han esperado un recuento menor que 5. El recuento mínimo esperado es 20.50.

b. Sólo se ha calculado para una tabla 2x2

COLESTEROL.

DESCRIPTIVE DATA.

Descriptivos

	Tratamiento2			Estadístico	Error estándar
Colesterol total	Metformina	Media		175.05	5.181
		95% de intervalo de confianza para la media	Límite inferior	164.68	
			Límite superior	185.43	
		Media recortada al 5%		173.62	
		Mediana		171.00	
		Varianza		1557.103	
		Desviación estándar		39.460	
		Mínimo		102	
		Máximo		291	
		Rango		189	
		Rango intercuartil		58	
		Asimetría		.500	.314
		Curtosis		.341	.618
	Met + Glb	Media		182.10	5.341
		95% de intervalo de confianza para la media	Límite inferior	171.30	
			Límite superior	192.90	
		Media recortada al 5%		182.25	
		Mediana		181.50	
		Varianza		1141.067	
		Desviación estándar		33.780	
		Mínimo		114	
		Máximo		250	
		Rango		136	
		Rango intercuartil		47	
		Asimetría		-.156	.374
		Curtosis		-.576	.733

NORMALITY TEST.

Pruebas de normalidad

		Kolmogorov-Smirnov[a]			Shapiro-Wilk		
	Tratamiento2	Estadístico	gl	Sig.	Estadístico	gl	Sig.
Colesterol total	Metformina	.054	58	.200*	.979	58	.391
	Met + Glb	.077	40	.200*	.981	40	.739

*. Esto es un límite inferior de la significación verdadera.

a. Corrección de significación de Lilliefors

T STUDENT AND LEVENE TEST.

Prueba de muestras independientes

		Prueba de Levene de igualdad de varianzas		prueba t para la igualdad de medias						
									95% de intervalo de confianza de la diferencia	
		F	Sig.	t	gl	Sig. (bilateral)	Diferencia de medias	Diferencia de error estándar	Inferior	Superior
Colesterol total	Se asumen varianzas iguales	1.007	.318	-.920	96	.360	-7.048	7.657	-22.248	8.151
	No se asumen varianzas iguales			-.947	91.500	.346	-7.048	7.441	-21.828	7.732

COLESTEROL: CONTROL VS NO CONTROL.

FREQUENCY TABLE WITH OBSERVED AND EXPECTED COUNTS AND PERCENTAGE.

Tabla cruzada Control ColTot*Tratamiento2

			Tratamiento2		Total
			Metformina	Met + Glb	
Control ColTot	Si control	Recuento	41	27	68
		Recuento esperado	40.2	27.8	68.0
		% dentro de Tratamiento2	70.7%	67.5%	69.4%
	No control	Recuento	17	13	30
		Recuento esperado	17.8	12.2	30.0
		% dentro de Tratamiento2	29.3%	32.5%	30.6%
Total		Recuento	58	40	98
		Recuento esperado	58.0	40.0	98.0
		% dentro de Tratamiento2	100.0%	100.0%	100.0%

STATISTICAL INFERENCE TEST: PEARSON´S CHI SQUARE TEST.

Pruebas de chi-cuadrado

	Valor	gl	Significación asintótica (bilateral)	Significación exacta (bilateral)	Significación exacta (unilateral)
Chi-cuadrado de Pearson	.113[a]	1	.736		
Corrección de continuidad[b]	.013	1	.909		
Razón de verosimilitud	.113	1	.737		
Prueba exacta de Fisher				.825	.453
Asociación lineal por lineal	.112	1	.738		
N de casos válidos	98				

a. 0 casillas (0.0%) han esperado un recuento menor que 5. El recuento mínimo esperado es 12.24.

b. Sólo se ha calculado para una tabla 2x2

HDL COLESTEROL.

DESCRIPTIVE DATA.

Descriptivos

Tratamiento2				Estadístico	Error estándar
CHDL	Metformina	Media		42.98	2.100
		95% de intervalo de confianza para la media	Límite inferior	38.75	
			Límite superior	47.20	
		Media recortada al 5%		42.00	
		Mediana		41.00	
		Varianza		211.736	
		Desviación estándar		14.551	
		Mínimo		13	
		Máximo		97	
		Rango		84	
		Rango intercuartil		12	
		Asimetría		1.464	.343
		Curtosis		3.395	.674
	Met + Glb	Media		40.97	1.967
		95% de intervalo de confianza para la media	Límite inferior	36.96	
			Límite superior	44.99	
		Media recortada al 5%		41.53	
		Mediana		41.50	
		Varianza		119.942	
		Desviación estándar		10.952	
		Mínimo		4	
		Máximo		63	
		Rango		59	
		Rango intercuartil		11	
		Asimetría		-.967	.421
		Curtosis		3.408	.821

NORMALITY TEST.

Pruebas de normalidad

		Kolmogorov-Smirnov[a]			Shapiro-Wilk		
	Tratamiento2	Estadístico	gl	Sig.	Estadístico	gl	Sig.
CHDL	Metformina	.200	48	.000	.876	48	.000
	Met + Glb	.138	31	.139	.923	31	.028

a. Corrección de significación de Lilliefors

MEDIAN AND QUARTILE RANGE.

Percentiles

			Percentiles						
		Tratamiento2	5	10	25	50	75	90	95
Promedio ponderado (Definición 1)	CHDL	Metformina	26.60	28.66	34.38	41.00	46.23	65.16	72.44
		Met + Glb	14.66	31.76	36.50	41.50	47.70	54.58	60.56
Bisagras de Tukey	CHDL	Metformina			34.55	41.00	45.75		
		Met + Glb			36.80	41.50	47.20		

MANN WHITNEY´S U TEST.

Estadísticos de prueba[a]

	CHDL
U de Mann-Whitney	719.500
W de Wilcoxon	1895.500
Z	-.246
Sig. asintótica (bilateral)	.806

a. Variable de agrupación: Tratamiento2

LDL COLESTEROL.

DESCRIPTIVE DATA.

Descriptivos

	Tratamiento2			Estadístico	Error estándar
CLDL	Metformina	Media		91.41	4.782
		95% de intervalo de confianza para la media	Límite inferior	81.78	
			Límite superior	101.05	
		Media recortada al 5%		90.57	
		Mediana		91.00	
		Varianza		1052.061	
		Desviación estándar		32.435	
		Mínimo		27	
		Máximo		188	
		Rango		161	
		Rango intercuartil		47	
		Asimetría		.413	.350
		Curtosis		.396	.688
	Met + Glb	Media		103.69	5.552
		95% de intervalo de confianza para la media	Límite inferior	92.31	
			Límite superior	115.06	
		Media recortada al 5%		103.98	
		Mediana		103.50	
		Varianza		893.756	
		Desviación estándar		29.896	
		Mínimo		43	
		Máximo		159	
		Rango		116	
		Rango intercuartil		38	
		Asimetría		-.097	.434
		Curtosis		-.186	.845

NORMALITY TEST.

Pruebas de normalidad

		Kolmogorov-Smirnov[a]			Shapiro-Wilk		
	Tratamiento2	Estadístico	gl	Sig.	Estadístico	gl	Sig.
CLDL	Metformina	.079	46	.200*	.980	46	.622
	Met + Glb	.099	29	.200*	.977	29	.761

*. Esto es un límite inferior de la significación verdadera.

a. Corrección de significación de Lilliefors

T STUDENT AND LEVENE TEST.

Prueba de muestras independientes

		Prueba de Levene de igualdad de varianzas		prueba t para la igualdad de medias						
									95% de intervalo de confianza de la diferencia	
		F	Sig.	t	gl	Sig. (bilateral)	Diferencia de medias	Diferencia de error estándar	Inferior	Superior
CLDL	Se asumen varianzas iguales	.634	.429	-1.644	73	.105	-12.272	7.466	-27.150	2.607
	No se asumen varianzas iguales			-1.675	63.290	.099	-12.272	7.327	-26.913	2.370

TRIGLYCERIDES.

DESCRIPTIVE DATA.

Descriptivos

	Tratamiento2			Estadístico	Error estándar
TAG	Metformina	Media		210.98	23.661
		95% de intervalo de confianza para la media	Límite inferior	163.54	
			Límite superior	258.42	
		Media recortada al 5%		187.55	
		Mediana		194.00	
		Varianza		30790.907	
		Desviación estándar		175.473	
		Mínimo		45	
		Máximo		1307	
		Rango		1262	
		Rango intercuartil		99	
		Asimetría		4.762	.322
		Curtosis		28.698	.634
	Met + Glb	Media		210.26	22.508
		95% de intervalo de confianza para la media	Límite inferior	164.66	
			Límite superior	255.87	
		Media recortada al 5%		189.68	
		Mediana		157.50	
		Varianza		19251.442	
		Desviación estándar		138.750	
		Mínimo		94	
		Máximo		788	
		Rango		694	
		Rango intercuartil		123	
		Asimetría		2.690	.383
		Curtosis		8.428	.750

NORMALITY TEST.

Pruebas de normalidad

		Kolmogorov-Smirnov[a]			Shapiro-Wilk		
	Tratamiento2	Estadístico	gl	Sig.	Estadístico	gl	Sig.
TAG	Metformina	.247	55	.000	.556	55	.000
	Met + Glb	.207	38	.000	.691	38	.000

a. Corrección de significación de Lilliefors

MEDIAN AND QUARTILE RANGE.

Percentiles

			Percentiles						
		Tratamiento2	5	10	25	50	75	90	95
Promedio ponderado (Definición 1)	TAG	Metformina	65.60	85.20	131.00	194.00	230.00	325.80	457.00
		Met + Glb	101.60	108.50	130.50	157.50	253.00	371.60	598.00
Bisagras de Tukey	TAG	Metformina			131.00	194.00	223.50		
		Met + Glb			131.00	157.50	252.00		

MANN WHITNEY´S U TEST.

Estadísticos de prueba[a]

	TAG
U de Mann-Whitney	1033.000
W de Wilcoxon	1774.000
Z	-.094
Sig. asintótica (bilateral)	.925

a. Variable de agrupación: Tratamiento2

TRIGLYCERIDES CONTROL.

FREQUENCY TABLE WITH OBSERVED AND EXPECTED COUNTS AND PERCENTAGE.

Tabla cruzada Control TAG*Tratamiento2

			Tratamiento2		Total
			Metformina	Met + Glb	
Control TAG	Si control	Recuento	16	17	33
		Recuento esperado	19.5	13.5	33.0
		% dentro de Tratamiento2	29.1%	44.7%	35.5%
	No control	Recuento	39	21	60
		Recuento esperado	35.5	24.5	60.0
		% dentro de Tratamiento2	70.9%	55.3%	64.5%
Total		Recuento	55	38	93
		Recuento esperado	55.0	38.0	93.0
		% dentro de Tratamiento2	100.0%	100.0%	100.0%

STATISTICAL INFERENCE TEST: PEARSON´S CHI SQUARE TEST.

Pruebas de chi-cuadrado

	Valor	gl	Significación asintótica (bilateral)	Significación exacta (bilateral)	Significación exacta (unilateral)
Chi-cuadrado de Pearson	2.403[a]	1	.121		
Corrección de continuidad[b]	1.768	1	.184		
Razón de verosimilitud	2.389	1	.122		
Prueba exacta de Fisher				.130	.092
Asociación lineal por lineal	2.377	1	.123		
N de casos válidos	93				

a. 0 casillas (0.0%) han esperado un recuento menor que 5. El recuento mínimo esperado es 13.48.

b. Sólo se ha calculado para una tabla 2x2

GLOMERULAR FILTRATION RATE (MDRD-4).

DESCRIPTIVE DATA.

Descriptivos

	Tratamiento2			Estadístico	Error estándar
TFG MDRD 4	Metformina	Media		100.46	3.405
		95% de intervalo de confianza para la media	Límite inferior	93.63	
			Límite superior	107.29	
		Media recortada al 5%		100.58	
		Mediana		93.40	
		Varianza		637.681	
		Desviación estándar		25.252	
		Mínimo		44	
		Máximo		156	
		Rango		112	
		Rango intercuartil		29	
		Asimetría		.144	.322
		Curtosis		-.336	.634
	Met + Glb	Media		105.45	4.376
		95% de intervalo de confianza para la media	Límite inferior	96.63	
			Límite superior	114.28	
		Media recortada al 5%		105.91	
		Mediana		103.44	
		Varianza		842.534	
		Desviación estándar		29.026	
		Mínimo		42	
		Máximo		174	
		Rango		132	
		Rango intercuartil		44	
		Asimetría		-.031	.357
		Curtosis		-.194	.702

NORMALITY TEST.

Pruebas de normalidad

		Kolmogorov-Smirnov[a]			Shapiro-Wilk		
	Tratamiento2	Estadístico	gl	Sig.	Estadístico	gl	Sig.
TFG MDRD 4	Metformina	.132	55	.018	.975	55	.303
	Met + Glb	.086	44	.200*	.978	44	.556

*. Esto es un límite inferior de la significación verdadera.

a. Corrección de significación de Lilliefors

MEDIAN AND QUARTILE RANGE.

Percentiles

			Percentiles						
		Tratamiento2	5	10	25	50	75	90	95
Promedio ponderado (Definición 1)	TFG MDRD 4	Metformina	55.02	68.29	86.70	93.40	115.43	137.52	143.16
		Met + Glb	48.82	65.46	89.08	103.44	132.73	143.82	144.51
Bisagras de Tukey	TFG MDRD 4	Metformina			87.81	93.40	115.16		
		Met + Glb			89.23	103.44	131.70		

MANN WHITNEY´S U TEST.

Estadísticos de prueba[a]

	TFG MDRD 4
U de Mann-Whitney	1071.500
W de Wilcoxon	2611.500
Z	-.975
Sig. asintótica (bilateral)	.329

a. Variable de agrupación: Tratamiento2

METFORMIN (mg/kg/day).

DESCRIPTIVE DATA.

Descriptivos

	Tratamiento2			Estadístico	Error estándar
mgkgdía	Metformina	Media		19.66748	1.177450
		95% de intervalo de confianza para la media	Límite inferior	17.30782	
			Límite superior	22.02714	
		Media recortada al 5%		19.17738	
		Mediana		19.25500	
		Varianza		77.638	
		Desviación estándar		8.811227	
		Mínimo		7.720	
		Máximo		40.220	
		Rango		32.500	
		Rango intercuartil		14.098	
		Asimetría		.722	.319
		Curtosis		-.264	.628
	Met + Glb	Media		28.06628	1.698699
		95% de intervalo de confianza para la media	Límite inferior	24.63817	
			Límite superior	31.49439	
		Media recortada al 5%		27.80080	
		Mediana		29.20000	
		Varianza		124.080	
		Desviación estándar		11.139116	
		Mínimo		5.110	
		Máximo		55.310	
		Rango		50.200	
		Rango intercuartil		14.460	
		Asimetría		.202	.361
		Curtosis		-.073	.709

NORMALITY TEST.

Pruebas de normalidad

		Kolmogorov-Smirnov[a]			Shapiro-Wilk		
	Tratamiento2	Estadístico	gl	Sig.	Estadístico	gl	Sig.
mgkgdía	Metformina	.133	56	.015	.924	56	.002
	Met + Glb	.079	43	.200*	.984	43	.811

*. Esto es un límite inferior de la significación verdadera.

a. Corrección de significación de Lilliefors

MEDIAN AND QUARTILE RANGE.

Percentiles

			Percentiles						
		Tratamiento2	5	10	25	50	75	90	95
Promedio ponderado (Definición 1)	mgkgdía	Metformina	9.02615	9.88000	11.92250	19.25500	26.02000	32.95800	39.08450
		Met + Glb	11.08800	12.62800	20.85000	29.20000	35.31000	42.11200	49.97200
Bisagras de Tukey	mgkgdía	Metformina			11.92500	19.25500	25.84000		
		Met + Glb			21.55000	29.20000	34.21000		

MANN WHITNEY´S U TEST.

Estadísticos de prueba[a]

	mgkgdía
U de Mann-Whitney	638.500
W de Wilcoxon	2234.500
Z	-3.992
Sig. asintótica (bilateral)	.000

a. Variable de agrupación: Tratamiento2

METFORMINA DOSE.

DESCRIPTIVE DATA.

Descriptivos

	Tratamiento2			Estadístico	Error estándar
Dosis Met	Metformina	Media		1519.20	87.595
		95% de intervalo de confianza para la media	Límite inferior	1343.65	
			Límite superior	1694.74	
		Media recortada al 5%		1514.48	
		Mediana		1700.00	
		Varianza		429681.615	
		Desviación estándar		655.501	
		Mínimo		425	
		Máximo		2550	
		Rango		2125	
		Rango intercuartil		850	
		Asimetría		.256	.319
		Curtosis		-1.088	.628
	Met + Glb	Media		1949.42	103.109
		95% de intervalo de confianza para la media	Límite inferior	1741.34	
			Límite superior	2157.50	
		Media recortada al 5%		1986.18	
		Mediana		2125.00	
		Varianza		457157.392	
		Desviación estándar		676.134	
		Mínimo		500	
		Máximo		2550	
		Rango		2050	
		Rango intercuartil		850	
		Asimetría		-.637	.361
		Curtosis		-1.001	.709

NORMALITY TEST.

Pruebas de normalidad

		Kolmogorov-Smirnov[a]			Shapiro-Wilk		
	Tratamiento2	Estadístico	gl	Sig.	Estadístico	gl	Sig.
Dosis Met	Metformina	.239	56	.000	.835	56	.000
	Met + Glb	.301	43	.000	.789	43	.000

a. Corrección de significación de Lilliefors

MEDIAN AND QUARTILE RANGE.

Percentiles

			Percentiles						
		Tratamiento2	5	10	25	50	75	90	95
Promedio ponderado (Definición 1)	Dosis Met	Metformina	797.50	850.00	850.00	1700.00	1700.00	2550.00	2550.00
		Met + Glb	850.00	850.00	1700.00	2125.00	2550.00	2550.00	2550.00
Bisagras de Tukey	Dosis Met	Metformina			850.00	1700.00	1700.00		
		Met + Glb			1700.00	2125.00	2550.00		

MANN WHITNEY´S U TEST.

Estadísticos de prueba[a]

	Dosis Met
U de Mann-Whitney	773.000
W de Wilcoxon	2369.000
Z	-3.197
Sig. asintótica (bilateral)	.001

a. Variable de agrupación: Tratamiento2

S1.11.2 Hardy Weinberg Equilibrium.

SLC22A1 (rs72552763)

GENOTIPO

	N observado	N esperada	Residuo
GAT7GAT	44	43.6	.4
del/GAT	46	46.8	-.8
del/del	13	12.6	.4
Total	103		

Estadísticos de prueba

	GENOTIPO
Chi-cuadrado	.033[a]
gl	2
Sig. asintótica	.984

a. 0 casillas (0.0%) han esperado frecuencias menores que 5. La frecuencia mínima de casilla esperada es 12.6.

SLC22A1 (rs622342)

GENOTIPO

	N observado	N esperada	Residuo
CC	94	93.2	.8
CA	8	9.5	-1.5
AA	1	.2	.8
Total	103		

Estadísticos de prueba

	GENOTIPO
Chi-cuadrado	3.957[a]
gl	2
Sig. asintótica	.138

a. 0 casillas (0.0%) han esperado frecuencias menores que 5. La frecuencia mínima de casilla esperada es 17.1.

SLC22A2 (rs316019)

GENOTIPO

	N observado	N esperada	Residuo
CC	94	93.2	.8
CA	8	9.5	-1.5
AA	1	.2	.8
Total	103		

Estadísticos de prueba

	GENOTIPO
Chi-cuadrado	2.652[a]
gl	2
Sig. asintótica	.265

a. 1 casillas (33.3%) han esperado frecuencias menores que 5. La frecuencia mínima de casilla esperada es .2.

SLC22A3 (rs2076828)

GENOTIPO

	N observado	N esperada	Residuo
CC	78	76.0	2.0
CG	21	24.9	-3.9
GG	4	2.0	2.0
Total	103		

Estadísticos de prueba

	GENOTIPO
Chi-cuadrado	2.547[a]
gl	2
Sig. asintótica	.280

a. 1 casillas (33.3%) han esperado frecuencias menores que 5. La frecuencia mínima de casilla esperada es 2.0.

ABCB1 (rs1128503)

GENOTIPO

	N observado	N esperada	Residuo
CC	27	23.8	3.2
CT	45	51.4	-6.4
TT	31	27.8	3.2
Total	103		

Estadísticos de prueba

	GENOTIPO
Chi-cuadrado	1.610[a]
gl	2
Sig. asintótica	.447

a. 0 casillas (0.0%) han esperado frecuencias menores que 5. La frecuencia mínima de casilla esperada es 23.8.

ABCB1 (rs1045642)

GENOTIPO

	N observado	N esperada	Residuo
CC	36	33.8	2.2
CT	46	50.4	-4.4
TT	21	18.8	2.2
Total	103		

Estadísticos de prueba

	GENOTIPO
Chi-cuadrado	.788[a]
gl	2
Sig. asintótica	.674

a. 0 casillas (0.0%) han esperado frecuencias menores que 5. La frecuencia mínima de casilla esperada es 18.8.

IVS8

GENOTIPO

	N observado	N esperada	Residuo
AA	69	66.1	2.9
AT	27	32.8	-5.8
TT	7	4.1	2.9
Total	103		

Estadísticos de prueba

	GENOTIPO
Chi-cuadrado	3.254[a]
gl	2
Sig. asintótica	.197

a. 1 casillas (33.3%) han esperado frecuencias menores que 5. La frecuencia mínima de casilla esperada es 4.1.

ABCB1 (rs2032582)

GENOTIPO

	N observado	N esperada	Residuo
GG	34	33.6	.4
GT	43	45.4	-2.4
TT	17	15.3	1.7
TA	2	3.1	-1.1
GA	6	4.5	1.5
Total	102		

Estadísticos de prueba

	GENOTIPO
Chi-cuadrado	1.195[a]
gl	4
Sig. asintótica	.879

a. 2 casillas (40.0%) han esperado frecuencias menores que 5. La frecuencia mínima de casilla esperada es 3.1.

CYP2C9

GENOTIPO

	N observado	N esperada	Residuo
*1/*1	85	85.3	-.3
*1/*2	10	10.0	.0
*2/*3	1	.4	.6
*1/*3	7	7.3	-.3
Total	103		

Estadísticos de prueba

	GENOTIPO
Chi-cuadrado	.805[a]
gl	3
Sig. asintótica	.848

a. 1 casillas (25.0%) han esperado frecuencias menores que 5. La frecuencia mínima de casilla esperada es .4.

S1.11.3 Table 3.

OCT1 rs72552763

Tabla cruzada OCT1 rs72552763*Control HbA1c

			Control HbA1c		Total
			Si control	No control	
OCT1 rs72552763	GAT/GAT	Recuento	23	21	44
		% dentro de Control HbA1c	47.9%	38.2%	42.7%
	del/GAT	Recuento	18	28	46
		% dentro de Control HbA1c	37.5%	50.9%	44.7%
	del/del	Recuento	7	6	13
		% dentro de Control HbA1c	14.6%	10.9%	12.6%
Total		Recuento	48	55	103
		% dentro de Control HbA1c	100.0%	100.0%	100.0%

Pruebas de chi-cuadrado

	Valor	gl	Significación asintótica (bilateral)
Chi-cuadrado de Pearson	1.875[a]	2	.392
Razón de verosimilitud	1.883	2	.390
Asociación lineal por lineal	.201	1	.654
N de casos válidos	103		

a. 0 casillas (0.0%) han esperado un recuento menor que 5. El recuento mínimo esperado es 6.06.

OCT1 rs622342

Tabla cruzada OCT1 rs622342*Control HbA1c

			Control HbA1c		Total
			Si control	No control	
OCT1 rs622342	AA	Recuento	22	19	41
		% dentro de Control HbA1c	45.8%	34.5%	39.8%
	AC	Recuento	15	25	40
		% dentro de Control HbA1c	31.3%	45.5%	38.8%
	CC	Recuento	11	11	22
		% dentro de Control HbA1c	22.9%	20.0%	21.4%
Total		Recuento	48	55	103
		% dentro de Control HbA1c	100.0%	100.0%	100.0%

Pruebas de chi-cuadrado

	Valor	gl	Significación asintótica (bilateral)
Chi-cuadrado de Pearson	2.254[a]	2	.324
Razón de verosimilitud	2.270	2	.321
Asociación lineal por lineal	.308	1	.579
N de casos válidos	103		

a. 0 casillas (0.0%) han esperado un recuento menor que 5. El recuento mínimo esperado es 10.25.

OCT2 rs316019

Tabla cruzada OCT2 rs316019*Control HbA1c

			Control HbA1c		Total
			Si control	No control	
OCT2 rs316019	CC	Recuento	43	51	94
		% dentro de Control HbA1c	89.6%	92.7%	91.3%
	AC	Recuento	5	3	8
		% dentro de Control HbA1c	10.4%	5.5%	7.8%
	AA	Recuento	0	1	1
		% dentro de Control HbA1c	0.0%	1.8%	1.0%
Total		Recuento	48	55	103
		% dentro de Control HbA1c	100.0%	100.0%	100.0%

Pruebas de chi-cuadrado

	Valor	gl	Significación asintótica (bilateral)
Chi-cuadrado de Pearson	1.713[a]	2	.425
Razón de verosimilitud	2.097	2	.350
Asociación lineal por lineal	.042	1	.838
N de casos válidos	103		

a. 4 casillas (66.7%) han esperado un recuento menor que 5. El recuento mínimo esperado es .47.

OCT3 rs2076828

Tabla cruzada OCT3 rs2076828*Control HbA1c

			Control HbA1c		Total
			Si control	No control	
OCT3 rs2076828	CC	Recuento	40	38	78
		% dentro de Control HbA1c	83.3%	69.1%	75.7%
	CG	Recuento	7	14	21
		% dentro de Control HbA1c	14.6%	25.5%	20.4%
	GG	Recuento	1	3	4
		% dentro de Control HbA1c	2.1%	5.5%	3.9%
Total		Recuento	48	55	103
		% dentro de Control HbA1c	100.0%	100.0%	100.0%

Pruebas de chi-cuadrado

	Valor	gl	Significación asintótica (bilateral)
Chi-cuadrado de Pearson	2.922[a]	2	.232
Razón de verosimilitud	3.000	2	.223
Asociación lineal por lineal	2.813	1	.094
N de casos válidos	103		

a. 2 casillas (33.3%) han esperado un recuento menor que 5. El recuento mínimo esperado es 1.86.

ABCB1 rs1128503.

Tabla cruzada ABCB1 rs1128503*Control HbA1c

			Control HbA1c		Total
			Si control	No control	
ABCB1 rs1128503	CC	Recuento	13	14	27
		% dentro de Control HbA1c	27.1%	25.5%	26.2%
	CT	Recuento	19	26	45
		% dentro de Control HbA1c	39.6%	47.3%	43.7%
	TT	Recuento	16	15	31
		% dentro de Control HbA1c	33.3%	27.3%	30.1%
Total		Recuento	48	55	103
		% dentro de Control HbA1c	100.0%	100.0%	100.0%

Pruebas de chi-cuadrado

	Valor	gl	Significación asintótica (bilateral)
Chi-cuadrado de Pearson	.686[a]	2	.710
Razón de verosimilitud	.687	2	.709
Asociación lineal por lineal	.089	1	.766
N de casos válidos	103		

a. 0 casillas (0.0%) han esperado un recuento menor que 5. El recuento mínimo esperado es 12.58.

ABCB1 rs2032582

Tabla cruzada ABCB1 rs2032582*Control HbA1c

			Control HbA1c		Total
			Si control	No control	
ABCB1 rs2032582	GG	Recuento	17	17	34
		% dentro de Control HbA1c	35.4%	31.5%	33.3%
	GT	Recuento	21	22	43
		% dentro de Control HbA1c	43.8%	40.7%	42.2%
	TT	Recuento	8	9	17
		% dentro de Control HbA1c	16.7%	16.7%	16.7%
	TA	Recuento	0	2	2
		% dentro de Control HbA1c	0.0%	3.7%	2.0%
	GA	Recuento	2	4	6
		% dentro de Control HbA1c	4.2%	7.4%	5.9%
Total		Recuento	48	54	102
		% dentro de Control HbA1c	100.0%	100.0%	100.0%

Pruebas de chi-cuadrado

	Valor	gl	Significación asintótica (bilateral)
Chi-cuadrado de Pearson	2.404[a]	4	.662
Razón de verosimilitud	3.181	4	.528
Asociación lineal por lineal	1.010	1	.315
N de casos válidos	102		

a. 4 casillas (40.0%) han esperado un recuento menor que 5. El recuento mínimo esperado es .94.

ABCB1 rs1045642.

Tabla cruzada ABCB1 rs1045642*Control HbA1c

			Control HbA1c		
			Si control	No control	Total
ABCB1 rs1045642	CC	Recuento	15	21	36
		% dentro de Control HbA1c	31.3%	38.2%	35.0%
	CT	Recuento	21	25	46
		% dentro de Control HbA1c	43.8%	45.5%	44.7%
	TT	Recuento	12	9	21
		% dentro de Control HbA1c	25.0%	16.4%	20.4%
Total		Recuento	48	55	103
		% dentro de Control HbA1c	100.0%	100.0%	100.0%

Pruebas de chi-cuadrado

	Valor	gl	Significación asintótica (bilateral)
Chi-cuadrado de Pearson	1.307[a]	2	.520
Razón de verosimilitud	1.307	2	.520
Asociación lineal por lineal	1.156	1	.282
N de casos válidos	103		

a. 0 casillas (0.0%) han esperado un recuento menor que 5. El recuento mínimo esperado es 9.79.

CYP2C9

Tabla cruzada GENCYP2C9*Control HbA1c

			Control HbA1c		
			Si control	No control	Total
GENCYP2C9	*1/*1	Recuento	39	46	85
		% dentro de Control HbA1c	81.3%	83.6%	82.5%
	*1/*2	Recuento	7	3	10
		% dentro de Control HbA1c	14.6%	5.5%	9.7%
	*1/*3	Recuento	2	5	7
		% dentro de Control HbA1c	4.2%	9.1%	6.8%
	*2/*3	Recuento	0	1	1
		% dentro de Control HbA1c	0.0%	1.8%	1.0%
Total		Recuento	48	55	103
		% dentro de Control HbA1c	100.0%	100.0%	100.0%

Pruebas de chi-cuadrado

	Valor	gl	Significación asintótica (bilateral)
Chi-cuadrado de Pearson	4.005[a]	3	.261
Razón de verosimilitud	4.461	3	.216
Asociación lineal por lineal	.250	1	.617
N de casos válidos	103		

a. 5 casillas (62.5%) han esperado un recuento menor que 5. El recuento mínimo esperado es .47.

IVS8

Tabla cruzada IVS8*Control HbA1c

			Control HbA1c		Total
			Si control	No control	
IVS8	AA	Recuento	30	39	69
		% dentro de Control HbA1c	62.5%	70.9%	67.0%
	AT	Recuento	14	13	27
		% dentro de Control HbA1c	29.2%	23.6%	26.2%
	TT	Recuento	4	3	7
		% dentro de Control HbA1c	8.3%	5.5%	6.8%
Total		Recuento	48	55	103
		% dentro de Control HbA1c	100.0%	100.0%	100.0%

Pruebas de chi-cuadrado

	Valor	gl	Significación asintótica (bilateral)
Chi-cuadrado de Pearson	.882[a]	2	.643
Razón de verosimilitud	.882	2	.644
Asociación lineal por lineal	.861	1	.353
N de casos válidos	103		

a. 2 casillas (33.3%) han esperado un recuento menor que 5. El recuento mínimo esperado es 3.26.

S1.11.4 Metformin plasmatic concentrations among DMT2 patients according to SNP and genotype.

OCT1 rs72552763

Resumen de procesamiento de casos

OCT1 rs72552763	Control HbA1c		Casos: Válido N	Válido Porcentaje	Perdidos N	Perdidos Porcentaje	Total N	Total Porcentaje
GAT/GAT	Si control	CONCMET	17	73.9%	6	26.1%	23	100.0%
	No control	CONCMET	17	81.0%	4	19.0%	21	100.0%
del/GAT	Si control	CONCMET	14	77.8%	4	22.2%	18	100.0%
	No control	CONCMET	27	96.4%	1	3.6%	28	100.0%
del/del	Si control	CONCMET	6	85.7%	1	14.3%	7	100.0%
	No control	CONCMET	5	83.3%	1	16.7%	6	100.0%

NORMALITY TEST.

Pruebas de normalidad

OCT1 rs72552763	Control HbA1c		Kolmogorov-Smirnov[a] Estadístico	gl	Sig.	Shapiro-Wilk Estadístico	gl	Sig.
GAT/GAT	Si control	CONCMET	.206	17	.053	.775	17	.001
	No control	CONCMET	.148	17	.200*	.915	17	.121
del/GAT	Si control	CONCMET	.313	14	.001	.599	14	.000
	No control	CONCMET	.137	27	.200*	.844	27	.001
del/del	Si control	CONCMET	.397	6	.004	.607	6	.001
	No control	CONCMET	.226	5	.200*	.875	5	.286

*. Esto es un límite inferior de la significación verdadera.

a. Corrección de significación de Lilliefors

DESCRIPTIVE DATA.

Percentiles

OCT1 rs72552763	Control HbA1c			Percentiles 5	10	25	50	75	90	95
GAT/GAT	Si control	Promedio ponderado (Definición 1)	CONCMET	62.869000	92.609000	129.834950	376.983900	819.813000	2172.083800	.
		Bisagras de Tukey	CONCMET			133.472000	376.983900	763.070000		
	No control	Promedio ponderado (Definición 1)	CONCMET	45.940000	87.517600	169.003000	481.759000	1000.998000	1300.516400	.
		Bisagras de Tukey	CONCMET			185.934000	481.759000	971.008000		
del/GAT	Si control	Promedio ponderado (Definición 1)	CONCMET	34.154000	34.752495	61.853000	137.112000	719.343250	3235.361000	.
		Bisagras de Tukey	CONCMET			66.470000	137.112000	687.450000		
	No control	Promedio ponderado (Definición 1)	CONCMET	14.537600	25.692200	210.800900	790.618000	1055.678000	1450.150000	2676.207000
		Bisagras de Tukey	CONCMET			228.851400	790.618000	1030.222500		
del/del	Si control	Promedio ponderado (Definición 1)	CONCMET	80.594990	80.594990	100.922173	139.152950	528.639925	.	.
		Bisagras de Tukey	CONCMET			107.697900	139.152950	264.307900		
	No control	Promedio ponderado (Definición 1)	CONCMET	22.222000	22.222000	46.232000	785.648000	1764.910000	.	.
		Bisagras de Tukey	CONCMET			70.242000	785.648000	1642.771000		

MANN WHITNEY´S U TEST.

Estadísticos de prueba[a]

OCT1 rs72552763		CONCMET
GAT/GAT	U de Mann-Whitney	137.000
	W de Wilcoxon	290.000
	Z	-.258
	Sig. asintótica (bilateral)	.796
	Significación exacta [2* (sig. unilateral)]	.812[b]
del/GAT	U de Mann-Whitney	131.000
	W de Wilcoxon	236.000
	Z	-1.595
	Sig. asintótica (bilateral)	.111
	Significación exacta [2* (sig. unilateral)]	.115[b]
del/del	U de Mann-Whitney	13.000
	W de Wilcoxon	34.000
	Z	-.365
	Sig. asintótica (bilateral)	.715
	Significación exacta [2* (sig. unilateral)]	.792[b]

a. Variable de agrupación: Control HbA1c

b. No corregido para empates.

INFERENTIAL ANALYISIS BY GROUP: KRUSKAL WALLIS TEST.

Rangos

Control HbA1c		OCT1 rs72552763	N	Rango promedio
Si control	CONCMET	GAT/GAT	17	21.76
		del/GAT	14	16.71
		del/del	6	16.50
		Total	37	
No control	CONCMET	GAT/GAT	17	23.24
		del/GAT	27	25.96
		del/del	5	25.80
		Total	49	

Estadísticos de prueba[a,b]

Control HbA1c		CONCMET
Si control	Chi-cuadrado	2.053
	gl	2
	Sig. asintótica	.358
No control	Chi-cuadrado	.398
	gl	2
	Sig. asintótica	.820

a. Prueba de Kruskal Wallis

b. Variable de agrupación: OCT1 rs72552763

OCT1 rs622342

Resumen de procesamiento de casos

Control HbA1c	OCT1 rs622342		Casos: Válido N	Válido Porcentaje	Perdidos N	Perdidos Porcentaje	Total N	Total Porcentaje
Si control	AA	CONCMET	16	72.7%	6	27.3%	22	100.0%
	AC	CONCMET	12	80.0%	3	20.0%	15	100.0%
	CC	CONCMET	9	81.8%	2	18.2%	11	100.0%
No control	AA	CONCMET	16	84.2%	3	15.8%	19	100.0%
	AC	CONCMET	23	92.0%	2	8.0%	25	100.0%
	CC	CONCMET	10	90.9%	1	9.1%	11	100.0%

NORMALITY TEST.

Pruebas de normalidad

Control HbA1c	OCT1 rs622342		Kolmogorov-Smirnov[a] Estadístico	gl	Sig.	Shapiro-Wilk Estadístico	gl	Sig.
Si control	AA	CONCMET	.202	16	.081	.784	16	.002
	AC	CONCMET	.312	12	.002	.616	12	.000
	CC	CONCMET	.326	9	.006	.724	9	.003
No control	AA	CONCMET	.172	16	.200*	.909	16	.113
	AC	CONCMET	.110	23	.200*	.953	23	.335
	CC	CONCMET	.176	10	.200*	.881	10	.135

*. Esto es un límite inferior de la significación verdadera.

a. Corrección de significación de Lilliefors

DESCRIPTIVE DATA.

Percentiles

Control HbA1c	OCT1 rs622342			Percentiles 5	10	25	50	75	90	95
Si control	AA	Promedio ponderado (Definición 1)	CONCMET	62.869000	96.931700	128.016425	339.770900	732.029725	1639.041200	.
		Bisagras de Tukey	CONCMET			129.834950	339.770900	700.989450		
	AC	Promedio ponderado (Definición 1)	CONCMET	34.154000	34.513097	52.619000	102.664500	776.392250	3756.570700	.
		Bisagras de Tukey	CONCMET			57.236000	102.664500	737.761500		
	CC	Promedio ponderado (Definición 1)	CONCMET	80.594990	80.594990	119.930900	157.857000	1004.543000	.	.
		Bisagras de Tukey	CONCMET			132.163900	157.857000	687.450000		
No control	AA	Promedio ponderado (Definición 1)	CONCMET	45.940000	82.320400	160.537500	526.347950	1015.993000	1351.124600	.
		Bisagras de Tukey	CONCMET			169.003000	526.347950	1000.998000		
	AC	Promedio ponderado (Definición 1)	CONCMET	23.059800	46.094996	246.901900	725.067000	1004.767000	1305.531400	1753.366200
		Bisagras de Tukey	CONCMET			254.538900	725.067000	969.059000		
	CC	Promedio ponderado (Definición 1)	CONCMET	9.018000	10.397900	58.385750	795.477000	1703.840500	3089.618200	.
		Bisagras de Tukey	CONCMET			70.242000	795.477000	1642.771000		

MANN WHITNEY´S U TEST.

Estadísticos de prueba[a]

OCT1 rs622342		CONCMET
AA	U de Mann-Whitney	114.000
	W de Wilcoxon	250.000
	Z	-.528
	Sig. asintótica (bilateral)	.598
	Significación exacta [2*(sig. unilateral)]	.616[b]
AC	U de Mann-Whitney	95.000
	W de Wilcoxon	173.000
	Z	-1.494
	Sig. asintótica (bilateral)	.135
	Significación exacta [2*(sig. unilateral)]	.141[b]
CC	U de Mann-Whitney	38.000
	W de Wilcoxon	83.000
	Z	-.572
	Sig. asintótica (bilateral)	.568
	Significación exacta [2*(sig. unilateral)]	.604[b]

a. Variable de agrupación: Control HbA1c

b. No corregido para empates.

INFERENTIAL ANALYISIS BY GROUP: KRUSKAL WALLIS TEST.

Estadísticos de prueba[a,b]

Control HbA1c		CONCMET
Si control	Chi-cuadrado	1.932
	gl	2
	Sig. asintótica	.381
No control	Chi-cuadrado	.394
	gl	2
	Sig. asintótica	.821

a. Prueba de Kruskal Wallis

b. Variable de agrupación: OCT1 rs622342

OCT2 rs316019

Resumen de procesamiento de casos

Control HbA1c	OCT2 rs316019		Casos					
			Válido		Perdidos		Total	
			N	Porcentaje	N	Porcentaje	N	Porcentaje
Si control	CC	CONCMET	34	79.1%	9	20.9%	43	100.0%
	AC	CONCMET	3	60.0%	2	40.0%	5	100.0%
No control	CC	CONCMET	46	90.2%	5	9.8%	51	100.0%
	AC	CONCMET	3	100.0%	0	0.0%	3	100.0%

NORMALITY TEST.

Pruebas de normalidad

Control HbA1c	OCT2 rs316019		Kolmogorov-Smirnov[a]			Shapiro-Wilk		
			Estadístico	gl	Sig.	Estadístico	gl	Sig.
Si control	CC	CONCMET	.263	34	.000	.652	34	.000
	AC	CONCMET	.365	3	.	.797	3	.107
No control	CC	CONCMET	.139	46	.027	.871	46	.000
	AC	CONCMET	.205	3	.	.993	3	.841

a. Corrección de significación de Lilliefors

DESCRIPTIVE DATA.

Percentiles

Control HbA1c	OCT2 rs316019			Percentiles						
				5	10	25	50	75	90	95
Si control	CC	Promedio ponderado (Definición 1)	CONCMET	35.051743	55.435500	103.974750	151.999500	706.355000	2117.643500	2839.195750
		Bisagras de Tukey	CONCMET			105.285000	151.999500	687.450000		
	AC	Promedio ponderado (Definición 1)	CONCMET	264.307900	264.307900	264.307900	302.557900	.	.	.
		Bisagras de Tukey	CONCMET			283.432900	302.557900	589.556950		
No control	CC	Promedio ponderado (Definición 1)	CONCMET	22.430250	40.081300	175.508750	648.426500	1037.160500	1661.554300	1876.063550
		Bisagras de Tukey	CONCMET			183.321000	648.426500	1030.988000		
	AC	Promedio ponderado (Definición 1)	CONCMET	210.800900	210.800900	210.800900	804.080000	.	.	.
		Bisagras de Tukey	CONCMET			507.440450	804.080000	1025.817000		

MANN WHITNEY´S U TEST.

Estadísticos de prueba[a]

OCT2 rs316019		CONCMET
CC	U de Mann-Whitney	612.000
	W de Wilcoxon	1207.000
	Z	-1.655
	Sig. asintótica (bilateral)	.098
AC	U de Mann-Whitney	4.000
	W de Wilcoxon	10.000
	Z	-.218
	Sig. asintótica (bilateral)	.827
	Significación exacta [2* (sig. unilateral)]	1.000[b]

a. Variable de agrupación: Control HbA1c

b. No corregido para empates.

MANN WHITNEY´S U TEST.

Estadísticos de prueba[a]

Control HbA1c		CONCMET
Si control	U de Mann-Whitney	35.000
	W de Wilcoxon	630.000
	Z	-.890
	Sig. asintótica (bilateral)	.373
	Significación exacta [2* (sig. unilateral)]	.407[b]
No control	U de Mann-Whitney	58.000
	W de Wilcoxon	1139.000
	Z	-.459
	Sig. asintótica (bilateral)	.646
	Significación exacta [2* (sig. unilateral)]	.679[b]

a. Variable de agrupación: OCT2 rs316019

b. No corregido para empates.

OCT3 rs2076828

NORMALITY TEST.

Pruebas de normalidad[c]

Control HbA1c	OCT3 rs2076828		Kolmogorov-Smirnov[a] Estadístico	gl	Sig.	Shapiro-Wilk Estadístico	gl	Sig.
Si control	CC	CONCMET	.267	30	.000	.645	30	.000
	CG	CONCMET	.182	6	.200*	.934	6	.610
No control	CC	CONCMET	.164	35	.018	.848	35	.000
	CG	CONCMET	.131	11	.200*	.949	11	.628
	GG	CONCMET	.175	3	.	1.000	3	.998

*. Esto es un límite inferior de la significación verdadera.

a. Corrección de significación de Lilliefors

c. CONCMET es constante en uno o más archivos segmentados. Se ha omitido.

DESCRIPTIVE DATA.

Percentiles[a]

Control HbA1c	OCT3 rs2076828			Percentiles 5	10	25	50	75	90	95
Si control	CC	Promedio ponderado (Definición 1)	CONCMET	34.812345	49.488700	103.974750	144.338500	776.058250	2129.703900	3246.917950
		Bisagras de Tukey	CONCMET			105.285000	144.338500	763.070000		
	CG	Promedio ponderado (Definición 1)	CONCMET	66.470000	66.470000	135.010250	320.645900	636.005925	.	.
		Bisagras de Tukey	CONCMET			157.857000	320.645900	618.857900		
No control	CC	Promedio ponderado (Definición 1)	CONCMET	19.581200	24.973400	107.889000	685.768000	1055.678000	1765.494000	2154.286600
		Bisagras de Tukey	CONCMET			126.981950	685.768000	1030.222500		
	CG	Promedio ponderado (Definición 1)	CONCMET	210.800900	218.021100	290.535000	611.085000	971.008000	1293.141600	.
		Bisagras de Tukey	CONCMET			386.147000	611.085000	880.813000		
	GG	Promedio ponderado (Definición 1)	CONCMET	416.887900	416.887900	416.887900	1030.988000	.	.	.
		Bisagras de Tukey	CONCMET			723.937950	1030.988000	1336.879500		

a. CONCMET es constante en uno o más archivos segmentados. Se ha omitido.

MANN WHITNEY´S U TEST.

Estadísticos de prueba[a]

OCT3 rs2076828		CONCMET
CC	U de Mann-Whitney	444.000
	W de Wilcoxon	909.000
	Z	-1.066
	Sig. asintótica (bilateral)	.286
CG	U de Mann-Whitney	17.000
	W de Wilcoxon	38.000
	Z	-1.608
	Sig. asintótica (bilateral)	.108
	Significación exacta [2* (sig. unilateral)]	.122[b]
GG	U de Mann-Whitney	1.000
	W de Wilcoxon	7.000
	Z	-.447
	Sig. asintótica (bilateral)	.655
	Significación exacta [2* (sig. unilateral)]	1.000[b]

a. Variable de agrupación: Control HbA1c

b. No corregido para empates.

INFERENTIAL ANALYISIS BY GROUP: KRUSKAL WALLIS TEST.

Estadísticos de prueba[a]

Control HbA1c		CONCMET
Si control	U de Mann-Whitney	82.000
	W de Wilcoxon	547.000
	Z	-.340
	Sig. asintótica (bilateral)	.734
	Significación exacta [2* (sig. unilateral)]	.756[b]
No control	U de Mann-Whitney	174.000
	W de Wilcoxon	804.000
	Z	-.476
	Sig. asintótica (bilateral)	.634
	Significación exacta [2* (sig. unilateral)]	.648[b]

a. Variable de agrupación: OCT3 rs2076828

b. No corregido para empates.

ABCB1 rs1128503

Resumen de procesamiento de casos

Control HbA1c	ABCB1 rs1128503		Casos					
			Válido		Perdidos		Total	
			N	Porcentaje	N	Porcentaje	N	Porcentaje
Si control	CC	CONCMET	9	69.2%	4	30.8%	13	100.0%
	CT	CONCMET	16	84.2%	3	15.8%	19	100.0%
	TT	CONCMET	12	75.0%	4	25.0%	16	100.0%
No control	CC	CONCMET	14	100.0%	0	0.0%	14	100.0%
	CT	CONCMET	22	84.6%	4	15.4%	26	100.0%
	TT	CONCMET	13	86.7%	2	13.3%	15	100.0%

NORMALITY TEST.

Pruebas de normalidad

Control HbA1c	ABCB1 rs1128503		Kolmogorov-Smirnov[a]			Shapiro-Wilk		
			Estadístico	gl	Sig.	Estadístico	gl	Sig.
Si control	CC	CONCMET	.222	9	.200*	.890	9	.198
	CT	CONCMET	.304	16	.000	.571	16	.000
	TT	CONCMET	.296	12	.005	.688	12	.001
No control	CC	CONCMET	.187	14	.199	.915	14	.184
	CT	CONCMET	.167	22	.113	.846	22	.003
	TT	CONCMET	.260	13	.016	.742	13	.001

*. Esto es un límite inferior de la significación verdadera.

a. Corrección de significación de Lilliefors

DESCRIPTIVE DATA.

Percentiles

Control HbA1c	ABCB1 rs1128503			Percentiles						
				5	10	25	50	75	90	95
Si control	CC	Promedio ponderado (Definición 1)	CONCMET	35.350990	35.350990	186.002900	763.070000	1727.177500	.	.
		Bisagras de Tukey	CONCMET			264.307900	763.070000	1321.636000		
	CT	Promedio ponderado (Definición 1)	CONCMET	48.002000	58.408900	85.457242	139.807000	655.102225	2782.243800	.
		Bisagras de Tukey	CONCMET			90.319495	139.807000	649.704450		
	TT	Promedio ponderado (Definición 1)	CONCMET	34.154000	49.643900	112.739250	150.196000	464.488925	1205.460500	.
		Bisagras de Tukey	CONCMET			113.948500	150.196000	402.643950		
No control	CC	Promedio ponderado (Definición 1)	CONCMET	22.817000	49.218995	136.528425	526.347950	1011.322250	1421.035500	.
		Bisagras de Tukey	CONCMET			146.074900	526.347950	1004.767000		
	CT	Promedio ponderado (Definición 1)	CONCMET	37.136150	123.534700	433.953000	844.766950	1133.265250	1598.399000	2995.558750
		Bisagras de Tukey	CONCMET			481.759000	844.766950	1095.169000		
	TT	Promedio ponderado (Definición 1)	CONCMET	9.018000	14.299600	58.091000	219.813900	667.410950	1874.494200	.
		Bisagras de Tukey	CONCMET			70.242000	219.813900	530.741900		

MANN WHITNEY´S U TEST.

Estadísticos de prueba[a]

ABCB1 rs1128503		CONCMET
CC	U de Mann-Whitney	51.000
	W de Wilcoxon	156.000
	Z	-.756
	Sig. asintótica (bilateral)	.450
	Significación exacta [2* (sig. unilateral)]	.477[b]
CT	U de Mann-Whitney	90.000
	W de Wilcoxon	226.000
	Z	-2.543
	Sig. asintótica (bilateral)	.011
	Significación exacta [2* (sig. unilateral)]	.010[b]
TT	U de Mann-Whitney	69.000
	W de Wilcoxon	147.000
	Z	-.490
	Sig. asintótica (bilateral)	.624
	Significación exacta [2* (sig. unilateral)]	.650[b]

a. Variable de agrupación: Control HbA1c

b. No corregido para empates.

INFERENTIAL ANALYISIS BY GROUP: KRUSKAL WALLIS TEST.

Estadísticos de prueba[a,b]

Control HbA1c		CONCMET
Si control	Chi-cuadrado	2.912
	gl	2
	Sig. asintótica	.233
No control	Chi-cuadrado	5.434
	gl	2
	Sig. asintótica	.066

a. Prueba de Kruskal Wallis

b. Variable de agrupación: ABCB1 rs1128503

ABCB1 rs2032582

Resumen de procesamiento de casos

Control HbA1c	ABCB1 rs2032582		Válido N	Válido Porcentaje	Perdidos N	Perdidos Porcentaje	Total N	Total Porcentaje
Si control	GG	CONCMET	13	76.5%	4	23.5%	17	100.0%
	GT	CONCMET	17	81.0%	4	19.0%	21	100.0%
	TT	CONCMET	6	75.0%	2	25.0%	8	100.0%
	GA	CONCMET	1	50.0%	1	50.0%	2	100.0%
No control	0	CONCMET	1	100.0%	0	0.0%	1	100.0%
	GG	CONCMET	16	94.1%	1	5.9%	17	100.0%
	GT	CONCMET	18	81.8%	4	18.2%	22	100.0%
	TT	CONCMET	9	100.0%	0	0.0%	9	100.0%
	TA	CONCMET	2	100.0%	0	0.0%	2	100.0%
	GA	CONCMET	3	75.0%	1	25.0%	4	100.0%

NORMALITY TEST.

Pruebas de normalidad[c]

Control HbA1c	ABCB1 rs2032582		Kolmogorov-Smirnov[a] Estadístico	gl	Sig.	Shapiro-Wilk Estadístico	gl	Sig.
Si control	GG	CONCMET	.217	13	.096	.819	13	.012
	GT	CONCMET	.340	17	.000	.531	17	.000
	TT	CONCMET	.207	6	.200*	.855	6	.174
No control	GG	CONCMET	.151	16	.200*	.937	16	.309
	GT	CONCMET	.113	18	.200*	.967	18	.730
	TT	CONCMET	.286	9	.033	.803	9	.022
	TA	CONCMET	.260	2	.			
	GA	CONCMET	.292	3	.	.923	3	.463

*. Esto es un límite inferior de la significación verdadera.

a. Corrección de significación de Lilliefors

c. CONCMET es constante en uno o más archivos segmentados. Se ha omitido.

DESCRIPTIVE DATA.

Percentiles[a]

Control HbA1c	ABCB1 rs2032582			5	10	25	50	75	90	95
Si control	GG	Promedio ponderado (Definición 1)	CONCMET	34.154000	34.632796	87.083950	618.857900	1068.329500	2250.813400	.
		Bisagras de Tukey	CONCMET			107.697900	618.857900	815.023000		
	GT	Promedio ponderado (Definición 1)	CONCMET	48.002000	59.895600	92.915500	146.142000	518.741950	2555.685200	.
		Bisagras de Tukey	CONCMET			100.044000	146.142000	376.983900		
	TT	Promedio ponderado (Definición 1)	CONCMET	111.530000	111.530000	134.783750	402.643950	872.453750	.	.
		Bisagras de Tukey	CONCMET			142.535000	402.643950	687.450000		
No control	GG	Promedio ponderado (Definición 1)	CONCMET	22.817000	25.332800	164.509650	788.133000	1049.505500	1433.759400	.
		Bisagras de Tukey	CONCMET			182.944400	788.133000	1043.333000		
	GT	Promedio ponderado (Definición 1)	CONCMET	9.018000	89.022600	243.115425	511.076900	888.457925	1110.407500	.
		Bisagras de Tukey	CONCMET			262.175900	511.076900	884.227900		
	TT	Promedio ponderado (Definición 1)	CONCMET	22.222000	22.222000	58.091000	210.800900	1472.319000	.	.
		Bisagras de Tukey	CONCMET			70.242000	210.800900	1088.976000		
	TA	Promedio ponderado (Definición 1)	CONCMET	1348.772000	1348.772000	1348.772000	2286.004500	.	.	.
		Bisagras de Tukey	CONCMET			1348.772000	2286.004500	3223.237000		
	GA	Promedio ponderado (Definición 1)	CONCMET	246.901900	246.901900	246.901900	611.085000	.	.	.
		Bisagras de Tukey	CONCMET			428.993450	611.085000	1158.233500		

a. CONCMET es constante en uno o más archivos segmentados. Se ha omitido.

MANN WHITNEY´S U TEST.

Estadísticos de prueba[a]

ABCB1 rs2032582		CONCMET
GG	U de Mann-Whitney	90.000
	W de Wilcoxon	181.000
	Z	-.614
	Sig. asintótica (bilateral)	.539
	Significación exacta [2*(sig. unilateral)]	.559[b]
GT	U de Mann-Whitney	91.000
	W de Wilcoxon	244.000
	Z	-2.046
	Sig. asintótica (bilateral)	.041
	Significación exacta [2*(sig. unilateral)]	.041[b]
TT	U de Mann-Whitney	26.000
	W de Wilcoxon	71.000
	Z	-.118
	Sig. asintótica (bilateral)	.906
	Significación exacta [2*(sig. unilateral)]	.955[b]
GA	U de Mann-Whitney	.000
	W de Wilcoxon	1.000
	Z	-1.342
	Sig. asintótica (bilateral)	.180
	Significación exacta [2*(sig. unilateral)]	.500[b]

a. Variable de agrupación: Control HbA1c

b. No corregido para empates.

INFERENTIAL ANALYISIS BY GROUP: KRUSKAL WALLIS TEST.

Estadísticos de prueba[a,b]

Control HbA1c		CONCMET
Si control	Chi-cuadrado	1.077
	gl	2
	Sig. asintótica	.584
No control	Chi-cuadrado	.621
	gl	2
	Sig. asintótica	.733

a. Prueba de Kruskal Wallis

b. Variable de agrupación: ABCB1 rs2032582

ABCB1 rs1045642

Resumen de procesamiento de casos

Control HbA1c	ABCB1 rs1045642		Casos: Válido N	Válido Porcentaje	Perdidos N	Perdidos Porcentaje	Total N	Total Porcentaje
Si control	CC	CONCMET	10	66.7%	5	33.3%	15	100.0%
	CT	CONCMET	17	81.0%	4	19.0%	21	100.0%
	TT	CONCMET	10	83.3%	2	16.7%	12	100.0%
No control	CC	CONCMET	19	90.5%	2	9.5%	21	100.0%
	CT	CONCMET	21	84.0%	4	16.0%	25	100.0%
	TT	CONCMET	9	100.0%	0	0.0%	9	100.0%

NORMALITY TEST.

Pruebas de normalidad

Control HbA1c	ABCB1 rs1045642		Kolmogorov-Smirnov[a] Estadístico	gl	Sig.	Shapiro-Wilk Estadístico	gl	Sig.
Si control	CC	CONCMET	.233	10	.131	.878	10	.125
	CT	CONCMET	.382	17	.000	.462	17	.000
	TT	CONCMET	.307	10	.008	.629	10	.000
No control	CC	CONCMET	.143	19	.200*	.952	19	.433
	CT	CONCMET	.156	21	.197	.834	21	.002
	TT	CONCMET	.234	9	.170	.852	9	.079

*. Esto es un límite inferior de la significación verdadera.

a. Corrección de significación de Lilliefors

DESCRIPTIVE DATA.

Percentiles

Control HbA1c	ABCB1 rs1045642			Percentiles 5	10	25	50	75	90	95
Si control	CC	Promedio ponderado (Definición 1)	CONCMET	34.154000	37.385600	116.721500	700.989450	1516.869000	2306.845500	.
		Bisagras de Tukey	CONCMET			133.472000	700.989450	1321.636000		
	CT	Promedio ponderado (Definición 1)	CONCMET	35.350990	45.471798	83.190995	116.367000	179.269000	954.943800	.
		Bisagras de Tukey	CONCMET			85.787000	116.367000	157.857000		
	TT	Promedio ponderado (Definición 1)	CONCMET	111.530000	114.630500	233.864675	414.445900	1014.283250	4074.085100	.
		Bisagras de Tukey	CONCMET			264.307900	414.445900	876.556000		
No control	CC	Promedio ponderado (Definición 1)	CONCMET	22.817000	26.411000	219.813900	785.648000	1030.988000	1344.183000	.
		Bisagras de Tukey	CONCMET			233.357900	785.648000	1017.877500		
	CT	Promedio ponderado (Definición 1)	CONCMET	17.907400	99.907400	184.627500	530.741900	1147.234500	1813.083800	3086.479500
		Bisagras de Tukey	CONCMET			185.934000	530.741900	1095.169000		
	TT	Promedio ponderado (Definición 1)	CONCMET	22.222000	22.222000	58.091000	290.535000	995.062000	.	.
		Bisagras de Tukey	CONCMET			70.242000	290.535000	901.148000		

MANN WHITNEY´S U TEST.

Estadísticos de prueba[a]

ABCB1 rs1045642		CONCMET
CC	U de Mann-Whitney	92.000
	W de Wilcoxon	282.000
	Z	-.138
	Sig. asintótica (bilateral)	.891
	Significación exacta [2*(sig. unilateral)]	.910[b]
CT	U de Mann-Whitney	72.000
	W de Wilcoxon	225.000
	Z	-3.127
	Sig. asintótica (bilateral)	.002
	Significación exacta [2*(sig. unilateral)]	.001[b]
TT	U de Mann-Whitney	38.000
	W de Wilcoxon	83.000
	Z	-.572
	Sig. asintótica (bilateral)	.568
	Significación exacta [2*(sig. unilateral)]	.604[b]

a. Variable de agrupación: Control HbA1c

b. No corregido para empates.

INFERENTIAL ANALYISIS BY GROUP: KRUSKAL WALLIS TEST.

Estadísticos de prueba[a,b]

Control HbA1c		CONCMET
Si control	Chi-cuadrado	8.602
	gl	2
	Sig. asintótica	.014
No control	Chi-cuadrado	.735
	gl	2
	Sig. asintótica	.692

a. Prueba de Kruskal Wallis

b. Variable de agrupación: ABCB1 rs1045642

POST HOC TEST: MANN WHITNEY´S U TEST.

CC vs CT*

Estadísticos de prueba[a]

Control HbA1c		CONCMET
Si control	U de Mann-Whitney	45.000
	W de Wilcoxon	198.000
	Z	-2.008
	Sig. asintótica (bilateral)	.045
	Significación exacta [2*(sig. unilateral)]	.046[b]
No control	U de Mann-Whitney	194.000
	W de Wilcoxon	425.000
	Z	-.149
	Sig. asintótica (bilateral)	.882
	Significación exacta [2*(sig. unilateral)]	.893[b]

a. Variable de agrupación: ABCB1 rs1045642

b. No corregido para empates.

CC vs TT

Estadísticos de prueba[a]

Control HbA1c		CONCMET
Si control	U de Mann-Whitney	47.000
	W de Wilcoxon	102.000
	Z	-.227
	Sig. asintótica (bilateral)	.821
	Significación exacta [2*(sig. unilateral)]	.853[b]
No control	U de Mann-Whitney	71.000
	W de Wilcoxon	116.000
	Z	-.713
	Sig. asintótica (bilateral)	.476
	Significación exacta [2*(sig. unilateral)]	.498[b]

a. Variable de agrupación: ABCB1 rs1045642

b. No corregido para empates.

CT* vs TT

Estadísticos de prueba[a]

Control HbA1c		CONCMET
Si control	U de Mann-Whitney	29.000
	W de Wilcoxon	182.000
	Z	-2.812
	Sig. asintótica (bilateral)	.005
	Significación exacta [2*(sig. unilateral)]	.004[b]
No control	U de Mann-Whitney	76.000
	W de Wilcoxon	121.000
	Z	-.837
	Sig. asintótica (bilateral)	.402
	Significación exacta [2*(sig. unilateral)]	.422[b]

a. Variable de agrupación: ABCB1 rs1045642

b. No corregido para empates.

CYP2C9.

Resumen de procesamiento de casos

Control HbA1c	GENCYP2C9		Casos: Válido N	Válido Porcentaje	Perdidos N	Perdidos Porcentaje	Total N	Total Porcentaje
Si control	*1/*1	CONCMET	29	74.4%	10	25.6%	39	100.0%
	*1/*2	CONCMET	7	100.0%	0	0.0%	7	100.0%
	*1/*3	CONCMET	1	50.0%	1	50.0%	2	100.0%
No control	*1/*1	CONCMET	41	89.1%	5	10.9%	46	100.0%
	*1/*2	CONCMET	3	100.0%	0	0.0%	3	100.0%
	*1/*3	CONCMET	4	80.0%	1	20.0%	5	100.0%
	*2/*3	CONCMET	1	100.0%	0	0.0%	1	100.0%

NORMALITY TEST.

Pruebas de normalidad[c]

Control HbA1c	GENCYP2C9		Kolmogorov-Smirnov[a] Estadístico	gl	Sig.	Shapiro-Wilk Estadístico	gl	Sig.
Si control	*1/*1	CONCMET	.249	29	.000	.688	29	.000
	*1/*2	CONCMET	.221	7	.200*	.851	7	.127
No control	*1/*1	CONCMET	.119	41	.154	.939	41	.030
	*1/*2	CONCMET	.175	3	.	1.000	3	.992
	*1/*3	CONCMET	.336	4	.	.802	4	.106

*. Esto es un límite inferior de la significación verdadera.

a. Corrección de significación de Lilliefors

c. CONCMET es constante en uno o más archivos segmentados. Se ha omitido.

DESCRIPTIVE DATA.

Percentiles[a]

Control HbA1c	GENCYP2C9			Percentiles 5	10	25	50	75	90	95
Si control	*1/*1	Promedio ponderado (Definición 1)	CONCMET	41.676495	62.869000	109.613950	200.681000	845.789500	2132.719000	3348.848500
		Bisagras de Tukey	CONCMET			111.530000	200.681000	815.023000		
	*1/*2	Promedio ponderado (Definición 1)	CONCMET	34.154000	34.154000	85.787000	278.954000	660.500000	.	.
		Bisagras de Tukey	CONCMET			92.915500	278.954000	481.528950		
No control	*1/*1	Promedio ponderado (Definición 1)	CONCMET	22.281500	30.316800	198.367450	725.067000	1043.333000	1347.854200	1834.372900
		Bisagras de Tukey	CONCMET			210.800900	725.067000	1030.988000		
	*1/*2	Promedio ponderado (Definición 1)	CONCMET	70.242000	70.242000	70.242000	107.889000	.	.	.
		Bisagras de Tukey	CONCMET			89.065500	107.889000	126.981950		
	*1/*3	Promedio ponderado (Definición 1)	CONCMET	262.175900	262.175900	319.484900	712.381450	2650.765500	.	.
		Bisagras de Tukey	CONCMET			376.793900	712.381450	2078.294000		

a. CONCMET es constante en uno o más archivos segmentados. Se ha omitido.

MANN WHITNEY´S U TEST.

Estadísticos de prueba[a]

GENCYP2C9		CONCMET
*1/*1	U de Mann-Whitney	492.000
	W de Wilcoxon	927.000
	Z	-1.222
	Sig. asintótica (bilateral)	.222
*1/*2	U de Mann-Whitney	7.000
	W de Wilcoxon	13.000
	Z	-.798
	Sig. asintótica (bilateral)	.425
	Significación exacta [2*(sig. unilateral)]	.517[b]
*1/*3	U de Mann-Whitney	.000
	W de Wilcoxon	1.000
	Z	-1.414
	Sig. asintótica (bilateral)	.157
	Significación exacta [2*(sig. unilateral)]	.400[b]

a. Variable de agrupación: Control HbA1c

b. No corregido para empates.

INFERENTIAL ANALYISIS BY GROUP: KRUSKAL WALLIS TEST.

Estadísticos de prueba[a,b]

Control HbA1c		CONCMET
Si control	Chi-cuadrado	.566
	gl	2
	Sig. asintótica	.753
No control	Chi-cuadrado	6.840
	gl	3
	Sig. asintótica	.077

a. Prueba de Kruskal Wallis

b. Variable de agrupación: GENCYP2C9

IVS 8

Resumen de procesamiento de casos

Control HbA1c	IVS8		Casos					
			Válido		Perdidos		Total	
			N	Porcentaje	N	Porcentaje	N	Porcentaje
Si control	AA	CONCMET	24	80.0%	6	20.0%	30	100.0%
	AT	CONCMET	10	71.4%	4	28.6%	14	100.0%
	TT	CONCMET	3	75.0%	1	25.0%	4	100.0%
No control	AA	CONCMET	33	84.6%	6	15.4%	39	100.0%
	AT	CONCMET	13	100.0%	0	0.0%	13	100.0%
	TT	CONCMET	3	100.0%	0	0.0%	3	100.0%

NORMALITY TEST.

Pruebas de normalidad

Control HbA1c	IVS8		Kolmogorov-Smirnov[a]			Shapiro-Wilk		
			Estadístico	gl	Sig.	Estadístico	gl	Sig.
Si control	AA	CONCMET	.295	24	.000	.579	24	.000
	AT	CONCMET	.203	10	.200*	.861	10	.077
	TT	CONCMET	.375	3	.	.773	3	.052
No control	AA	CONCMET	.148	33	.064	.846	33	.000
	AT	CONCMET	.165	13	.200*	.918	13	.238
	TT	CONCMET	.381	3	.	.760	3	.022

*. Esto es un límite inferior de la significación verdadera.

a. Corrección de significación de Lilliefors

DESCRIPTIVE DATA.

Percentiles

Control HbA1c	IVS8			Percentiles						
				5	10	25	50	75	90	95
Si control	AA	Promedio ponderado (Definición 1)	CONCMET	37.616000	57.236000	105.888225	151.999500	655.102225	2117.643500	3809.295250
		Bisagras de Tukey	CONCMET			106.491450	151.999500	649.704450		
	AT	Promedio ponderado (Definición 1)	CONCMET	35.350990	38.102791	76.163492	451.658900	1348.093250	2239.335200	.
		Bisagras de Tukey	CONCMET			80.594990	451.658900	1321.636000		
	TT	Promedio ponderado (Definición 1)	CONCMET	111.530000	111.530000	111.530000	133.472000	.	.	.
		Bisagras de Tukey	CONCMET			122.501000	133.472000	474.247500		
No control	AA	Promedio ponderado (Definición 1)	CONCMET	38.824600	84.537394	197.060950	530.741900	1092.072500	1562.738000	2287.905400
		Bisagras de Tukey	CONCMET			210.800900	530.741900	1088.976000		
	AT	Promedio ponderado (Definición 1)	CONCMET	26.411000	43.943400	238.234500	785.648000	1000.998000	1770.505600	.
		Bisagras de Tukey	CONCMET			290.535000	785.648000	971.008000		
	TT	Promedio ponderado (Definición 1)	CONCMET	9.018000	9.018000	9.018000	22.817000	.	.	.
		Bisagras de Tukey	CONCMET			15.917500	22.817000	539.247500		

MANN WHITNEY´S U TEST.

Estadísticos de prueba[a]

IVS8		CONCMET
AA	U de Mann-Whitney	275.000
	W de Wilcoxon	575.000
	Z	-1.956
	Sig. asintótica (bilateral)	.051
AT	U de Mann-Whitney	57.000
	W de Wilcoxon	112.000
	Z	-.496
	Sig. asintótica (bilateral)	.620
	Significación exacta [2*(sig. unilateral)]	.648[b]
TT	U de Mann-Whitney	3.000
	W de Wilcoxon	9.000
	Z	-.655
	Sig. asintótica (bilateral)	.513
	Significación exacta [2*(sig. unilateral)]	.700[b]

a. Variable de agrupación: Control HbA1c

b. No corregido para empates.

INFERENTIAL ANALYISIS BY GROUP: KRUSKAL WALLIS TEST.

Estadísticos de prueba[a,b]

Control HbA1c		CONCMET
Si control	Chi-cuadrado	.521
	gl	2
	Sig. asintótica	.770
No control	Chi-cuadrado	1.965
	gl	2
	Sig. asintótica	.374

a. Prueba de Kruskal Wallis

b. Variable de agrupación: IVS8

S1.11.5 Metformin concentration according to treatment and genotype.

OCT1 rs72552763

METFORMIN

Resumen de procesamiento de casos[a]

	OCT1 rs72552763	Casos					
		Válido		Perdidos		Total	
		N	Porcentaje	N	Porcentaje	N	Porcentaje
CONCMET	GAT/GAT	20	71.4%	8	28.6%	28	100.0%
	del/GAT	19	86.4%	3	13.6%	22	100.0%
	del/del	7	77.8%	2	22.2%	9	100.0%

a. Tratamiento2 = Metformina

METFORMIN + GLIBENCLAMIDE

Resumen de procesamiento de casos[a]

	OCT1 rs72552763	Casos					
		Válido		Perdidos		Total	
		N	Porcentaje	N	Porcentaje	N	Porcentaje
CONCMET	GAT/GAT	14	87.5%	2	12.5%	16	100.0%
	del/GAT	22	91.7%	2	8.3%	24	100.0%
	del/del	4	100.0%	0	0.0%	4	100.0%

a. Tratamiento2 = Met + Glb

NORMALITY TEST

Pruebas de normalidad[a] (Metformin)

	OCT1 rs72552763	Kolmogorov-Smirnov[b]			Shapiro-Wilk		
		Estadístico	gl	Sig.	Estadístico	gl	Sig.
CONCMET	GAT/GAT	.180	20	.087	.825	20	.002
	del/GAT	.278	19	.000	.579	19	.000
	del/del	.369	7	.004	.705	7	.004

a. Tratamiento2 = Metformina

b. Corrección de significación de Lilliefors

Pruebas de normalidad[a] (Metformin + Glibenclamide)

	OCT1 rs72552763	Kolmogorov-Smirnov[b]			Shapiro-Wilk		
		Estadístico	gl	Sig.	Estadístico	gl	Sig.
CONCMET	GAT/GAT	.198	14	.142	.870	14	.042
	del/GAT	.152	22	.200*	.864	22	.006
	del/del	.262	4	.	.863	4	.273

*. Esto es un límite inferior de la significación verdadera.

a. Tratamiento2 = Met + Glb

b. Corrección de significación de Lilliefors

DECRIPTIVE DATA

Percentiles[a] (Metformin)

		OCT1 rs72552763	Percentiles 5	10	25	50	75	90	95
Promedio ponderado (Definición 1)	CONCMET	GAT/GAT	64.621150	98.125200	128.016425	504.046450	947.395000	2062.193600	2319.701800
		del/GAT	9.018000	26.411000	48.002000	262.175900	805.306000	1348.772000	.
		del/del	80.594990	80.594990	107.697900	146.142000	1321.636000	.	.
Bisagras de Tukey	CONCMET	GAT/GAT			129.834950	504.046450	923.782000		
		del/GAT			57.236000	262.175900	765.186500		
		del/del			119.930900	146.142000	792.971950		

a. Tratamiento2 = Metformina

Percentiles[a] (Metformin + Glibenclamide)

		OCT1 rs72552763	Percentiles 5	10	25	50	75	90	95
Promedio ponderado (Definición 1)	CONCMET	GAT/GAT	45.940000	76.914500	177.468500	366.072950	806.013500	1452.341000	.
		del/GAT	30.737599	78.670793	176.955000	797.349000	1128.620500	2028.496200	3055.136650
		del/del	22.222000	22.222000	34.227000	427.945000	1611.698750	.	.
Bisagras de Tukey	CONCMET	GAT/GAT			185.934000	366.072950	731.022000		
		del/GAT			183.321000	797.349000	1088.976000		
		del/del			46.232000	427.945000	1336.348500		

a. Tratamiento2 = Met + Glb

INFERENTIAL ANALYSIS

BETWEEN TREATMENT: MANN WHITNEY´S U TEST

Estadísticos de prueba[a,b]

	CONCMET
U de Mann-Whitney	135.000
W de Wilcoxon	240.000
Z	-.175
Sig. asintótica (bilateral)	.861
Significación exacta [2*(sig. unilateral)]	.877[c]

a. OCT1 rs72552763 = GAT/GAT

b. Variable de agrupación: Tratamiento2

c. No corregido para empates.

GAT/GAT

Estadísticos de prueba[a,b]

	CONCMET
U de Mann-Whitney	139.000
W de Wilcoxon	329.000
Z	-1.830
Sig. asintótica (bilateral)	.067

a. OCT1 rs72552763 = del/GAT

b. Variable de agrupación: Tratamiento2

Del/GAT

Estadísticos de prueba[a,b]

	CONCMET
U de Mann-Whitney	12.000
W de Wilcoxon	22.000
Z	-.378
Sig. asintótica (bilateral)	.705
Significación exacta [2* (sig. unilateral)]	.788[c]

a. OCT1 rs72552763 = del/del

b. Variable de agrupación: Tratamiento2

c. No corregido para empates.

Del/del

BETWEEN GENOTYPE: KRUSKAL WALLIS TEST

Estadísticos de prueba[a,b,c]

	CONCMET
Chi-cuadrado	1.541
gl	2
Sig. asintótica	.463

a. Tratamiento2 = Metformina

b. Prueba de Kruskal Wallis

c. Variable de agrupación: OCT1 rs72552763

Estadísticos de prueba[a,b,c]

	CONCMET
Chi-cuadrado	1.555
gl	2
Sig. asintótica	.459

a. Tratamiento2 = Met + Glb

b. Prueba de Kruskal Wallis

c. Variable de agrupación: OCT1 rs72552763

OCT1 rs622342

METFORMIN

Resumen de procesamiento de casos[a]

	OCT1 rs622342	Casos					
		Válido		Perdidos		Total	
		N	Porcentaje	N	Porcentaje	N	Porcentaje
CONCMET	AA	18	72.0%	7	28.0%	25	100.0%
	AC	16	84.2%	3	15.8%	19	100.0%
	CC	12	80.0%	3	20.0%	15	100.0%

a. Tratamiento2 = Metformina

METFORMIN + GLIBENCLAMIDE

Resumen de procesamiento de casos[a]

	OCT1 rs622342	Casos					
		Válido		Perdidos		Total	
		N	Porcentaje	N	Porcentaje	N	Porcentaje
CONCMET	AA	14	87.5%	2	12.5%	16	100.0%
	AC	19	90.5%	2	9.5%	21	100.0%
	CC	7	100.0%	0	0.0%	7	100.0%

a. Tratamiento2 = Met + Glb

NORMALITY TEST

Pruebas de normalidad[a]

	OCT1 rs622342	Kolmogorov-Smirnov[b]			Shapiro-Wilk		
		Estadístico	gl	Sig.	Estadístico	gl	Sig.
CONCMET	AA	.176	18	.147	.846	18	.007
	AC	.306	16	.000	.627	16	.000
	CC	.279	12	.011	.825	12	.018

a. Tratamiento2 = Metformina

b. Corrección de significación de Lilliefors

Pruebas de normalidad[a]

	OCT1 rs622342	Kolmogorov-Smirnov[b]			Shapiro-Wilk		
		Estadístico	gl	Sig.	Estadístico	gl	Sig.
CONCMET	AA	.198	14	.142	.870	14	.042
	AC	.126	19	.200*	.944	19	.308
	CC	.222	7	.200*	.876	7	.210

*. Esto es un límite inferior de la significación verdadera.

a. Tratamiento2 = Met + Glb

b. Corrección de significación de Lilliefors

DECRIPTIVE DATA

Percentiles[a]

		OCT1 rs622342	Percentiles 5	10	25	50	75	90	95
Promedio ponderado (Definición 1)	CONCMET	AA	62.869000	94.407700	123.740175	451.658900	900.169000	1497.990400	.
		AC	26.411000	31.831100	52.619000	270.564950	792.534000	2941.126300	.
		CC	9.018000	30.491097	113.814400	237.554400	1192.553500	1554.571300	.
Bisagras de Tukey	CONCMET	AA			126.197900	451.658900	876.556000		
		AC			57.236000	270.564950	770.045000		
		CC			119.930900	237.554400	1063.471000		

a. Tratamiento2 = Metformina

Percentiles[a]

		OCT1 rs622342	Percentiles 5	10	25	50	75	90	95
Promedio ponderado (Definición 1)	CONCMET	AA	45.940000	76.914500	177.468500	366.072950	806.013500	1452.341000	.
		AC	22.222000	75.620990	183.321000	790.618000	1055.678000	1344.183000	.
		CC	22.817000	22.817000	70.242000	785.648000	2102.568000	.	.
Bisagras de Tukey	CONCMET	AA			185.934000	366.072950	731.022000		
		AC			215.111450	790.618000	1030.222500		
		CC			114.049500	785.648000	1994.808500		

a. Tratamiento2 = Met + Glb

INFERENTIAL ANALYSIS

BETWEEN TREATMENT: MANN WHITNEY´S U TEST

Estadísticos de prueba[a,b]

	CONCMET
U de Mann-Whitney	124.000
W de Wilcoxon	295.000
Z	-.076
Sig. asintótica (bilateral)	.939
Significación exacta [2*(sig. unilateral)]	.955[c]

a. OCT1 rs622342 = AA

b. Variable de agrupación: Tratamiento2

c. No corregido para empates.

A/A

Estadísticos de prueba[a,b]

	CONCMET
U de Mann-Whitney	109.000
W de Wilcoxon	245.000
Z	-1.424
Sig. asintótica (bilateral)	.154
Significación exacta [2*(sig. unilateral)]	.161[c]

a. OCT1 rs622342 = AC

b. Variable de agrupación: Tratamiento2

c. No corregido para empates.

A/C

Estadísticos de prueba[a,b]

	CONCMET
U de Mann-Whitney	33.000
W de Wilcoxon	111.000
Z	-.761
Sig. asintótica (bilateral)	.447
Significación exacta [2*(sig. unilateral)]	.482[c]

a. OCT1 rs622342 = CC

b. Variable de agrupación: Tratamiento2

c. No corregido para empates.

C/C

BETWEEN GENOTYPE: KRUSKAL WALLIS TEST

Estadísticos de prueba[a,b,c]

	CONCMET
Chi-cuadrado	.896
gl	2
Sig. asintótica	.639

a. Tratamiento2 = Metformina

b. Prueba de Kruskal Wallis

c. Variable de agrupación: OCT1 rs622342

Estadísticos de prueba[a,b,c]

	CONCMET
Chi-cuadrado	.766
gl	2
Sig. asintótica	.682

a. Tratamiento2 = Met + Glb

b. Prueba de Kruskal Wallis

c. Variable de agrupación: OCT1 rs622342

OCT2 rs316019

METFORMIN

Resumen de procesamiento de casos[a]

		OCT2 rs316019	Casos					
			Válido		Perdidos		Total	
			N	Porcentaje	N	Porcentaje	N	Porcentaje
CONCMET	CC		42	80.8%	10	19.2%	52	100.0%
	AC		4	66.7%	2	33.3%	6	100.0%

a. Tratamiento2 = Metformina

METFORMIN + GLIBENCLAMIDE

Resumen de procesamiento de casos[a]

		OCT2 rs316019	Casos					
			Válido		Perdidos		Total	
			N	Porcentaje	N	Porcentaje	N	Porcentaje
CONCMET	CC		38	90.5%	4	9.5%	42	100.0%
	AC		2	100.0%	0	0.0%	2	100.0%

a. Tratamiento2 = Met + Glb

NORMALITY TEST

Pruebas de normalidad[a,c]

		OCT2 rs316019	Kolmogorov-Smirnov[b]			Shapiro-Wilk		
			Estadístico	gl	Sig.	Estadístico	gl	Sig.
CONCMET	CC		.224	42	.000	.712	42	.000
	AC		.389	4	.	.742	4	.032

a. Tratamiento2 = Metformina

b. Corrección de significación de Lilliefors

c. No hay casos válidos para CONCMET cuando OCT2 rs316019 = 3.000. Los estadísticos no se pueden calcular para este nivel.

Pruebas de normalidad[a]

		OCT2 rs316019	Kolmogorov-Smirnov[b]			Shapiro-Wilk		
			Estadístico	gl	Sig.	Estadístico	gl	Sig.
CONCMET	CC		.166	38	.010	.841	38	.000
	AC		.260	2	.			

a. Tratamiento2 = Met + Glb

b. Corrección de significación de Lilliefors

DECRIPTIVE DATA

Percentiles[a,b]

		OCT2 rs316019	Percentiles						
			5	10	25	50	75	90	95
Promedio ponderado (Definición 1)	CONCMET	CC	27.572450	39.146293	103.974750	327.968950	836.554250	1578.179200	2300.019400
		AC	210.800900	210.800900	224.177650	283.432900	733.056475	.	.
Bisagras de Tukey	CONCMET	CC			105.285000	327.968950	815.023000		
		AC			237.554400	283.432900	589.556950		

a. Tratamiento2 = Metformina

b. No hay casos válidos para CONCMET cuando OCT2 rs316019 = 3.000. Los estadísticos no se pueden calcular para este nivel.

Percentiles[a]

		OCT2 rs316019	Percentiles						
			5	10	25	50	75	90	95
Promedio ponderado (Definición 1)	CONCMET	CC	22.787250	67.811800	156.410750	511.076900	1037.160500	1858.800700	2158.601450
		AC	804.080000	804.080000	804.080000	1025.817000	.	.	.
Bisagras de Tukey	CONCMET	CC			157.857000	511.076900	1030.988000		
		AC			804.080000	1025.817000	1247.554000		

a. Tratamiento2 = Met + Glb

INFERENTIAL ANALYSIS

BETWEEN TREATMENT: MANN WHITNEY´S U TEST

Estadísticos de prueba[a,b]

	CONCMET
U de Mann-Whitney	684.000
W de Wilcoxon	1587.000
Z	-1.098
Sig. asintótica (bilateral)	.272

a. OCT2 rs316019 = CC

b. Variable de agrupación: Tratamiento2

C/C

Estadísticos de prueba[a,b]

	CONCMET
U de Mann-Whitney	1.000
W de Wilcoxon	11.000
Z	-1.389
Sig. asintótica (bilateral)	.165
Significación exacta [2*(sig. unilateral)]	.267[c]

a. OCT2 rs316019 = AC

b. Variable de agrupación: Tratamiento2

c. No corregido para empates.

A/C

Advertencias

No hay suficientes casos válidos para procesar en el archivo segmentado OCT2 rs316019=AA. No se han calculado estadísticas.

A/A

BETWEEN GENOTYPE: MANN WHITNEY´S U TEST

Estadísticos de prueba[a,b]

	CONCMET
U de Mann-Whitney	76.000
W de Wilcoxon	979.000
Z	-.312
Sig. asintótica (bilateral)	.755
Significación exacta [2*(sig. unilateral)]	.778[c]

a. Tratamiento2 = Metformina

b. Variable de agrupación: OCT2 rs316019

c. No corregido para empates.

Estadísticos de prueba[a,b]

	CONCMET
U de Mann-Whitney	19.000
W de Wilcoxon	760.000
Z	-1.179
Sig. asintótica (bilateral)	.238
Significación exacta [2*(sig. unilateral)]	.282[c]

a. Tratamiento2 = Met + Glb

b. Variable de agrupación: OCT2 rs316019

c. No corregido para empates.

OCT3 rs2076828

METFORMIN

Resumen de procesamiento de casos[a]

	OCT3 rs2076828	Casos					
		Válido		Perdidos		Total	
		N	Porcentaje	N	Porcentaje	N	Porcentaje
CONCMET	CC	35	76.1%	11	23.9%	46	100.0%
	CG	9	81.8%	2	18.2%	11	100.0%
	GG	2	100.0%	0	0.0%	2	100.0%

a. Tratamiento2 = Metformina

METFORMIN + GLIBENCLAMIDE

Resumen de procesamiento de casos[a]

	OCT3 rs2076828	Casos					
		Válido		Perdidos		Total	
		N	Porcentaje	N	Porcentaje	N	Porcentaje
CONCMET	CC	30	93.8%	2	6.3%	32	100.0%
	CG	8	80.0%	2	20.0%	10	100.0%
	GG	2	100.0%	0	0.0%	2	100.0%

a. Tratamiento2 = Met + Glb

NORMALITY TEST

Pruebas de normalidad[a]

	OCT3 rs2076828	Kolmogorov-Smirnov[b]			Shapiro-Wilk		
		Estadístico	gl	Sig.	Estadístico	gl	Sig.
CONCMET	CC	.249	35	.000	.663	35	.000
	CG	.118	9	.200*	.980	9	.962
	GG	.260	2	.			

*. Esto es un límite inferior de la significación verdadera.

a. Tratamiento2 = Metformina

b. Corrección de significación de Lilliefors

Pruebas de normalidad[a]

	OCT3 rs2076828	Kolmogorov-Smirnov[b]			Shapiro-Wilk		
		Estadístico	gl	Sig.	Estadístico	gl	Sig.
CONCMET	CC	.187	30	.009	.844	30	.000
	CG	.162	8	.200*	.930	8	.514
	GG	.260	2	.			

*. Esto es un límite inferior de la significación verdadera.

a. Tratamiento2 = Met + Glb

b. Corrección de significación de Lilliefors

DECRIPTIVE DATA

Percentiles[a]

		OCT3 rs2076828	Percentiles 5	10	25	50	75	90	95
Promedio ponderado (Definición 1)	CONCMET	CC	22.932400	34.872194	100.044000	146.142000	815.023000	1709.566600	2737.265200
		CG	66.470000	66.470000	237.554400	481.759000	706.258500	.	.
		GG	1321.636000	1321.636000	1321.636000	1482.203500	.	.	.
Bisagras de Tukey	CONCMET	CC			102.664500	146.142000	810.164500		
		CG			264.307900	481.759000	687.450000		
		GG			1321.636000	1482.203500	1642.771000		

a. Tratamiento2 = Metformina

Percentiles[a]

		OCT3 rs2076828	Percentiles 5	10	25	50	75	90	95
Promedio ponderado (Definición 1)	CONCMET	CC	22.549250	48.370200	136.528425	608.254950	1091.583500	1883.910300	2606.869050
		CG	157.857000	157.857000	257.810175	551.248450	1014.386500	.	.
		GG	416.887900	416.887900	416.887900	723.937950	.	.	.
Bisagras de Tukey	CONCMET	CC			146.074900	608.254950	1055.678000		
		CG			268.718450	551.248450	939.797000		
		GG			416.887900	723.937950	1030.988000		

a. Tratamiento2 = Met + Glb

INFERENTIAL ANALYSIS

BETWEEN TREATMENT: MANN WHITNEY´S U TEST

Estadísticos de prueba[a,b]

	CONCMET
U de Mann-Whitney	429.000
W de Wilcoxon	1059.000
Z	-1.263
Sig. asintótica (bilateral)	.206

a. OCT3 rs2076828 = CC

b. Variable de agrupación: Tratamiento2

C/C

Estadísticos de prueba[a,b]

	CONCMET
U de Mann-Whitney	30.000
W de Wilcoxon	75.000
Z	-.577
Sig. asintótica (bilateral)	.564
Significación exacta [2*(sig. unilateral)]	.606[c]

a. OCT3 rs2076828 = CG

b. Variable de agrupación: Tratamiento2

c. No corregido para empates.

C/G

Estadísticos de prueba[a,b]

	CONCMET
U de Mann-Whitney	.000
W de Wilcoxon	3.000
Z	-1.549
Sig. asintótica (bilateral)	.121
Significación exacta [2*(sig. unilateral)]	.333[c]

a. OCT3 rs2076828 = GG

b. Variable de agrupación: Tratamiento2

c. No corregido para empates.

G/G

BETWEEN GENOTYPE: KRUSKAL WALLIS TEST

Estadísticos de prueba[a,b,c]

	CONCMET
Chi-cuadrado	4.239
gl	2
Sig. asintótica	.120

a. Tratamiento2 = Metformina

b. Prueba de Kruskal Wallis

c. Variable de agrupación: OCT3 rs2076828

Estadísticos de prueba[a,b,c]

	CONCMET
Chi-cuadrado	.261
gl	2
Sig. asintótica	.878

a. Tratamiento2 = Met + Glb

b. Prueba de Kruskal Wallis

c. Variable de agrupación: OCT3 rs2076828

ABCB1 rs1128503

METFORMIN

Resumen de procesamiento de casos[a]

	ABCB1 rs1128503	Casos					
		Válido		Perdidos		Total	
		N	Porcentaje	N	Porcentaje	N	Porcentaje
CONCMET	CC	11	73.3%	4	26.7%	15	100.0%
	CT	24	85.7%	4	14.3%	28	100.0%
	TT	11	68.8%	5	31.3%	16	100.0%

a. Tratamiento2 = Metformina

METFORMIN + GLIBENCLAMIDE

Resumen de procesamiento de casos[a]

	ABCB1 rs1128503	Casos					
		Válido		Perdidos		Total	
		N	Porcentaje	N	Porcentaje	N	Porcentaje
CONCMET	CC	12	100.0%	0	0.0%	12	100.0%
	CT	14	82.4%	3	17.6%	17	100.0%
	TT	14	93.3%	1	6.7%	15	100.0%

a. Tratamiento2 = Met + Glb

NORMALITY TEST

Pruebas de normalidad[a]

	ABCB1 rs1128503	Kolmogorov-Smirnov[b]			Shapiro-Wilk		
		Estadístico	gl	Sig.	Estadístico	gl	Sig.
CONCMET	CC	.184	11	.200*	.930	11	.411
	CT	.268	24	.000	.576	24	.000
	TT	.279	11	.016	.736	11	.001

*. Esto es un límite inferior de la significación verdadera.

a. Tratamiento2 = Metformina

b. Corrección de significación de Lilliefors

Pruebas de normalidad[a]

	ABCB1 rs1128503	Kolmogorov-Smirnov[b]			Shapiro-Wilk		
		Estadístico	gl	Sig.	Estadístico	gl	Sig.
CONCMET	CC	.216	12	.128	.882	12	.094
	CT	.193	14	.167	.880	14	.057
	TT	.263	14	.009	.715	14	.001

a. Tratamiento2 = Met + Glb

b. Corrección de significación de Lilliefors

DECRIPTIVE DATA

Percentiles[a]

		ABCB1 rs1128503	Percentiles						
			5	10	25	50	75	90	95
Promedio ponderado (Definición 1)	CONCMET	CC	35.350990	49.820372	264.307900	815.023000	1642.771000	2290.178200	.
		CT	31.808750	55.435500	98.445000	282.366900	785.246250	1221.970500	3613.308500
		TT	9.018000	14.045200	111.530000	142.535000	526.333900	1279.462000	.
Bisagras de Tukey	CONCMET	CC			441.582900	815.023000	1482.203500		
		CT			98.978000	282.366900	765.186500		
		TT			113.948500	142.535000	402.643950		

a. Tratamiento2 = Metformina

Percentiles[a]

		ABCB1 rs1128503	Percentiles						
			5	10	25	50	75	90	95
Promedio ponderado (Definición 1)	CONCMET	CC	22.817000	38.658197	117.435475	344.256400	951.229750	1148.806400	.
		CT	183.321000	236.928000	719.708500	994.514500	1434.482750	2662.902500	.
		TT	22.222000	34.081000	81.900750	210.247450	599.076425	1871.355500	.
Bisagras de Tukey	CONCMET	CC			126.981950	344.256400	897.692500		
		CT			731.022000	994.514500	1344.183000		
		TT			85.787000	210.247450	530.741900		

a. Tratamiento2 = Met + Glb

INFERENTIAL ANALYSIS

BETWEEN TREATMENT: MANN WHITNEY´S U TEST

Estadísticos de prueba[a,b]

	CONCMET
U de Mann-Whitney	41.000
W de Wilcoxon	119.000
Z	-1.539
Sig. asintótica (bilateral)	.124
Significación exacta [2*(sig. unilateral)]	.134[c]

a. ABCB1 rs1128503 = CC

b. Variable de agrupación: Tratamiento2

c. No corregido para empates.

C/C

Estadísticos de prueba[a,b]

	CONCMET
U de Mann-Whitney	65.000
W de Wilcoxon	365.000
Z	-3.117
Sig. asintótica (bilateral)	.002
Significación exacta [2* (sig. unilateral)]	.001[c]

a. ABCB1 rs1128503 = CT

b. Variable de agrupación: Tratamiento2

c. No corregido para empates.

C/T

Estadísticos de prueba[a,b]

	CONCMET
U de Mann-Whitney	65.000
W de Wilcoxon	131.000
Z	-.657
Sig. asintótica (bilateral)	.511
Significación exacta [2* (sig. unilateral)]	.536[c]

a. ABCB1 rs1128503 = TT

b. Variable de agrupación: Tratamiento2

c. No corregido para empates.

T/T

BETWEEN GENOTYPE: KRUSKAL WALLIS TEST

Estadísticos de prueba[a,b,c]

	CONCMET
Chi-cuadrado	5.193
gl	2
Sig. asintótica	.075

a. Tratamiento2 = Metformina

b. Prueba de Kruskal Wallis

c. Variable de agrupación: ABCB1 rs1128503

Estadísticos de prueba[a,b,c]

	CONCMET
Chi-cuadrado	10.026
gl	2
Sig. asintótica	.007

a. Tratamiento2 = Met + Glb

b. Prueba de Kruskal Wallis

c. Variable de agrupación: ABCB1 rs1128503

POST HOC ANALYSIS.

CC vs CT*

Estadísticos de prueba[a,b]

	CONCMET
U de Mann-Whitney	34.000
W de Wilcoxon	112.000
Z	-2.572
Sig. asintótica (bilateral)	.010
Significación exacta [2* (sig. unilateral)]	.009[c]

a. Tratamiento2 = Met + Glb

b. Variable de agrupación: ABCB1 rs1128503

c. No corregido para empates.

CC vs TT

Estadísticos de prueba[a,b]

	CONCMET
U de Mann-Whitney	75.000
W de Wilcoxon	180.000
Z	-.463
Sig. asintótica (bilateral)	.643
Significación exacta [2* (sig. unilateral)]	.667[c]

a. Tratamiento2 = Met + Glb

b. Variable de agrupación: ABCB1 rs1128503

c. No corregido para empates.

CT* vs TT

Estadísticos de prueba[a,b]

	CONCMET
U de Mann-Whitney	37.000
W de Wilcoxon	142.000
Z	-2.803
Sig. asintótica (bilateral)	.005
Significación exacta [2* (sig. unilateral)]	.004[c]

a. Tratamiento2 = Met + Glb

b. Variable de agrupación: ABCB1 rs1128503

c. No corregido para empates.

ABCB1 rs2032582

METFORMIN

Resumen de procesamiento de casos[a]

	ABCB1 rs2032582	Casos					
		Válido		Perdidos		Total	
		N	Porcentaje	N	Porcentaje	N	Porcentaje
CONCMET	GG	15	78.9%	4	21.1%	19	100.0%
	GT	22	81.5%	5	18.5%	27	100.0%
	TT	7	77.8%	2	22.2%	9	100.0%
	TA	1	100.0%	0	0.0%	1	100.0%
	GA	1	33.3%	2	66.7%	3	100.0%

a. Tratamiento2 = Metformina

METFORMIN + GLIBENCLAMIDE

Resumen de procesamiento de casos[a]

	ABCB1 rs2032582	Casos					
		Válido		Perdidos		Total	
		N	Porcentaje	N	Porcentaje	N	Porcentaje
CONCMET	GG	14	93.3%	1	6.7%	15	100.0%
	GT	13	81.3%	3	18.8%	16	100.0%
	TT	8	100.0%	0	0.0%	8	100.0%
	TA	1	100.0%	0	0.0%	1	100.0%
	GA	3	100.0%	0	0.0%	3	100.0%

a. Tratamiento2 = Met + Glb

NORMALITY TEST

Pruebas de normalidad[a,d,e]

	ABCB1 rs2032582	Kolmogorov-Smirnov[b]			Shapiro-Wilk		
		Estadístico	gl	Sig.	Estadístico	gl	Sig.
CONCMET	GG	.190	15	.151	.838	15	.012
	GT	.267	22	.000	.554	22	.000
	TT	.241	7	.200*	.812	7	.054

*. Esto es un límite inferior de la significación verdadera.

a. Tratamiento2 = Metformina

b. Corrección de significación de Lilliefors

d. CONCMET es constante cuando ABCB1 rs2032582 = TA. Se ha omitido.

e. CONCMET es constante cuando ABCB1 rs2032582 = GA. Se ha omitido.

Pruebas de normalidad[a,d]

	ABCB1 rs2032582	Kolmogorov-Smirnov[b]			Shapiro-Wilk		
		Estadístico	gl	Sig.	Estadístico	gl	Sig.
CONCMET	GG	.170	14	.200*	.921	14	.227
	GT	.223	13	.077	.787	13	.005
	TT	.269	8	.091	.820	8	.047
	GA	.292	3	.	.923	3	.463

*. Esto es un límite inferior de la significación verdadera.

a. Tratamiento2 = Met + Glb

b. Corrección de significación de Lilliefors

d. CONCMET es constante cuando ABCB1 rs2032582 = TA. Se ha omitido.

DECRIPTIVE DATA

Percentiles[a,b,c]

		ABCB1 rs2032582	Percentiles 5	10	25	50	75	90	95
Promedio ponderado (Definición 1)	CONCMET	GG	26.411000	31.056800	66.470000	618.857900	1321.636000	2211.448600	.
		GT	14.865600	52.462100	99.511000	282.366900	823.118500	1057.920700	3877.206250
		TT	111.530000	111.530000	142.535000	278.954000	687.450000	.	.
Bisagras de Tukey	CONCMET	GG			87.083950	618.857900	1068.329500		
		GT			100.044000	282.366900	805.306000		
		TT			176.667950	278.954000	606.891950		

a. Tratamiento2 = Metformina

b. CONCMET es constante cuando ABCB1 rs2032582 = TA. Se ha omitido.

c. CONCMET es constante cuando ABCB1 rs2032582 = GA. Se ha omitido.

Percentiles[a,b]

		ABCB1 rs2032582	Percentiles 5	10	25	50	75	90	95
Promedio ponderado (Definición 1)	CONCMET	GG	22.817000	65.353000	201.379150	788.133000	1037.160500	1271.741500	.
		GT	85.787000	114.615000	184.627500	416.887900	784.997950	1760.562400	.
		TT	22.222000	22.222000	52.015500	478.076000	1663.990500	.	.
		GA	246.901900	246.901900	246.901900	611.085000	.	.	.
Bisagras de Tukey	CONCMET	GG			219.813900	788.133000	1030.988000		
		GT			185.934000	416.887900	685.768000		
		TT			58.091000	478.076000	1472.319000		
		GA			428.993450	611.085000	1158.233500		

a. Tratamiento2 = Met + Glb

b. CONCMET es constante cuando ABCB1 rs2032582 = TA. Se ha omitido.

INFERENTIAL ANALYSIS

BETWEEN TREATMENT: MANN WHITNEY´S U TEST

Estadísticos de prueba[a,b]

	CONCMET
U de Mann-Whitney	90.000
W de Wilcoxon	210.000
Z	-.655
Sig. asintótica (bilateral)	.513
Significación exacta [2*(sig. unilateral)]	.533[c]

a. ABCB1 rs2032582 = GG

b. Variable de agrupación: Tratamiento2

c. No corregido para empates.

G/G

Estadísticos de prueba[a,b]

	CONCMET
U de Mann-Whitney	115.000
W de Wilcoxon	368.000
Z	-.956
Sig. asintótica (bilateral)	.339
Significación exacta [2*(sig. unilateral)]	.353[c]

a. ABCB1 rs2032582 = GT

b. Variable de agrupación: Tratamiento2

c. No corregido para empates.

G/T

Estadísticos de prueba[a,b]

	CONCMET
U de Mann-Whitney	28.000
W de Wilcoxon	64.000
Z	.000
Sig. asintótica (bilateral)	1.000
Significación exacta [2*(sig. unilateral)]	1.000[c]

a. ABCB1 rs2032582 = TT

b. Variable de agrupación: Tratamiento2

c. No corregido para empates.

T/T

BETWEEN GENOTYPE: KRUSKAL WALLIS TEST

Estadísticos de prueba[a,b,c]

	CONCMET
Chi-cuadrado	.247
gl	2
Sig. asintótica	.884

a. Tratamiento2 = Metformina

b. Prueba de Kruskal Wallis

c. Variable de agrupación: ABCB1 rs2032582

Estadísticos de prueba[a,b,c]

	CONCMET
Chi-cuadrado	3.740
gl	4
Sig. asintótica	.442

a. Tratamiento2 = Met + Glb

b. Prueba de Kruskal Wallis

c. Variable de agrupación: ABCB1 rs2032582

ABCB1 rs1045642

METFORMIN

Resumen de procesamiento de casos[a]

		ABCB1 rs1045642	Válido N	Válido Porcentaje	Perdidos N	Perdidos Porcentaje	Total N	Total Porcentaje
CONCMET		CC	11	64.7%	6	35.3%	17	100.0%
		CT	24	82.8%	5	17.2%	29	100.0%
		TT	11	84.6%	2	15.4%	13	100.0%

a. Tratamiento2 = Metformina

METFORMIN + GLIBENCLAMIDE

Resumen de procesamiento de casos[a]

		ABCB1 rs1045642	Válido N	Válido Porcentaje	Perdidos N	Perdidos Porcentaje	Total N	Total Porcentaje
CONCMET		CC	18	94.7%	1	5.3%	19	100.0%
		CT	14	82.4%	3	17.6%	17	100.0%
		TT	8	100.0%	0	0.0%	8	100.0%

a. Tratamiento2 = Met + Glb

NORMALITY TEST

Pruebas de normalidad[a]

	ABCB1 rs1045642	Kolmogorov-Smirnov[b] Estadístico	gl	Sig.	Shapiro-Wilk Estadístico	gl	Sig.
CONCMET	CC	.215	11	.164	.825	11	.020
	CT	.263	24	.000	.781	24	.000
	TT	.317	11	.003	.634	11	.000

a. Tratamiento2 = Metformina

b. Corrección de significación de Lilliefors

Pruebas de normalidad[a]

	ABCB1 rs1045642	Kolmogorov-Smirnov[b] Estadístico	gl	Sig.	Shapiro-Wilk Estadístico	gl	Sig.
CONCMET	CC	.129	18	.200*	.962	18	.650
	CT	.282	14	.004	.689	14	.000
	TT	.273	8	.080	.812	8	.038

*. Esto es un límite inferior de la significación verdadera.

a. Tratamiento2 = Met + Glb

b. Corrección de significación de Lilliefors

DECRIPTIVE DATA

Percentiles[a]

		ABCB1 rs1045642	Percentiles 5	10	25	50	75	90	95
Promedio ponderado (Definición 1)	CONCMET	CC	26.411000	27.959600	66.470000	618.857900	815.023000	2127.961600	.
		CT	15.601248	41.676495	98.445000	139.152950	785.246250	1495.771500	2010.232000
		TT	111.530000	117.731000	264.307900	526.333900	901.148000	3780.016200	.
Bisagras de Tukey	CONCMET	CC			99.971000	618.857900	789.046500		
		CT			98.978000	139.152950	765.186500		
		TT			271.630950	526.333900	888.852000		

a. Tratamiento2 = Metformina

Percentiles[a]

		ABCB1 rs1045642	Percentiles 5	10	25	50	75	90	95
Promedio ponderado (Definición 1)	CONCMET	CC	22.817000	70.340591	392.933650	837.422950	1103.647000	1745.100600	.
		CT	85.787000	96.838000	154.911475	308.784450	814.151000	2539.449500	.
		TT	22.222000	22.222000	52.015500	221.303500	1017.752000	.	.
Bisagras de Tukey	CONCMET	CC			441.610900	837.422950	1055.678000		
		CT			157.857000	308.784450	685.768000		
		TT			58.091000	221.303500	946.528000		

a. Tratamiento2 = Met + Glb

INFERENTIAL ANALYSIS

BETWEEN TREATMENT: MANN WHITNEY´S U TEST

Estadísticos de prueba[a,b]

	CONCMET
U de Mann-Whitney	68.000
W de Wilcoxon	134.000
Z	-1.393
Sig. asintótica (bilateral)	.164
Significación exacta [2*(sig. unilateral)]	.173[c]

a. ABCB1 rs1045642 = CC

b. Variable de agrupación: Tratamiento2

c. No corregido para empates.

C/C

Estadísticos de prueba[a,b]

	CONCMET
U de Mann-Whitney	126.000
W de Wilcoxon	426.000
Z	-1.271
Sig. asintótica (bilateral)	.204
Significación exacta [2* (sig. unilateral)]	.212[c]

a. ABCB1 rs1045642 = CT

b. Variable de agrupación: Tratamiento2

c. No corregido para empates.

C/T

Estadísticos de prueba[a,b]

	CONCMET
U de Mann-Whitney	32.000
W de Wilcoxon	68.000
Z	-.991
Sig. asintótica (bilateral)	.322
Significación exacta [2* (sig. unilateral)]	.351[c]

a. ABCB1 rs1045642 = TT

b. Variable de agrupación: Tratamiento2

c. No corregido para empates.

T/T

BETWEEN GENOTYPE: KRUSKAL WALLIS TEST

Estadísticos de prueba[a,b,c]

	CONCMET
Chi-cuadrado	2.708
gl	2
Sig. asintótica	.258

a. Tratamiento2 = Metformina

b. Prueba de Kruskal Wallis

c. Variable de agrupación: ABCB1 rs1045642

Estadísticos de prueba[a,b,c]

	CONCMET
Chi-cuadrado	3.480
gl	2
Sig. asintótica	.175

a. Tratamiento2 = Met + Glb

b. Prueba de Kruskal Wallis

c. Variable de agrupación: ABCB1 rs1045642

CYP2C9

METFORMIN

Resumen de procesamiento de casos[a]

	GENCYP2C9	Casos					
		Válido		Perdidos		Total	
		N	Porcentaje	N	Porcentaje	N	Porcentaje
CONCMET	*1/*1	39	78.0%	11	22.0%	50	100.0%
	*1/*2	6	100.0%	0	0.0%	6	100.0%
	*1/*3	1	33.3%	2	66.7%	3	100.0%

a. Tratamiento2 = Metformina

METFORMIN + GLIBENCLAMIDE

Resumen de procesamiento de casos[a]

	GENCYP2C9	Casos					
		Válido		Perdidos		Total	
		N	Porcentaje	N	Porcentaje	N	Porcentaje
CONCMET	*1/*1	31	88.6%	4	11.4%	35	100.0%
	*1/*2	4	100.0%	0	0.0%	4	100.0%
	*1/*3	4	100.0%	0	0.0%	4	100.0%
	*2/*3	1	100.0%	0	0.0%	1	100.0%

a. Tratamiento2 = Met + Glb

NORMALITY TEST

Pruebas de normalidad[a,d]

	GENCYP2C9	Kolmogorov-Smirnov[b]			Shapiro-Wilk		
		Estadístico	gl	Sig.	Estadístico	gl	Sig.
CONCMET	*1/*1	.219	39	.000	.724	39	.000
	*1/*2	.226	6	.200*	.885	6	.291

*. Esto es un límite inferior de la significación verdadera.

a. Tratamiento2 = Metformina

b. Corrección de significación de Lilliefors

d. CONCMET es constante cuando GENCYP2C9 = *1/*3. Se ha omitido.

Pruebas de normalidad[a,d]

	GENCYP2C9	Kolmogorov-Smirnov[b]			Shapiro-Wilk		
		Estadístico	gl	Sig.	Estadístico	gl	Sig.
CONCMET	*1/*1	.125	31	.200*	.918	31	.021
	*1/*2	.194	4	.	.958	4	.769
	*1/*3	.327	4	.	.826	4	.158

*. Esto es un límite inferior de la significación verdadera.

a. Tratamiento2 = Met + Glb

b. Corrección de significación de Lilliefors

d. CONCMET es constante cuando GENCYP2C9 = *2/*3. Se ha omitido.

DECRIPTIVE DATA

Percentiles[a,b]

		GENCYP2C9	Percentiles 5	10	25	50	75	90	95
Promedio ponderado (Definición 1)	CONCMET	*1/*1	26.411000	48.002000	107.697900	376.983900	901.148000	1642.771000	2329.543000
		*1/*2	34.154000	34.154000	83.571500	290.755950	667.237500	.	.
Bisagras de Tukey	CONCMET	*1/*1			109.613950	376.983900	888.852000		
		*1/*2			100.044000	290.755950	660.500000		

a. Tratamiento2 = Metformina

b. CONCMET es constante cuando GENCYP2C9 = *1/*3. Se ha omitido.

Percentiles[a,b]

		GENCYP2C9	Percentiles 5	10	25	50	75	90	95
Promedio ponderado (Definición 1)	CONCMET	*1/*1	22.579000	51.876198	200.681000	685.768000	1055.678000	1753.366200	1973.256600
		*1/*2	70.242000	70.242000	74.128250	96.838000	136.528425	.	.
		*1/*3	157.857000	157.857000	241.245725	712.381450	2650.765500	.	.
Bisagras de Tukey	CONCMET	*1/*1			210.247450	685.768000	1043.333000		
		*1/*2			78.014500	96.838000	126.981950		
		*1/*3			324.634450	712.381450	2078.294000		

a. Tratamiento2 = Met + Glb

b. CONCMET es constante cuando GENCYP2C9 = *2/*3. Se ha omitido.

INFERENTIAL ANALYSIS

BETWEEN TREATMENT: MANN WHITNEY´S U TEST

Estadísticos de prueba[a,b]

	CONCMET
U de Mann-Whitney	506.000
W de Wilcoxon	1286.000
Z	-1.165
Sig. asintótica (bilateral)	.244

a. GENCYP2C9 = *1/*1

b. Variable de agrupación: Tratamiento2

***1/*1**

Estadísticos de prueba[a,b]

	CONCMET
U de Mann-Whitney	6.000
W de Wilcoxon	16.000
Z	-1.279
Sig. asintótica (bilateral)	.201
Significación exacta [2* (sig. unilateral)]	.257[c]

a. GENCYP2C9 = *1/*2

b. Variable de agrupación: Tratamiento2

c. No corregido para empates.

***1/*2**

BETWEEN GENOTYPE: KRUSKAL WALLIS TEST

Estadísticos de prueba[a,b,c]

	CONCMET
Chi-cuadrado	.492
gl	1
Sig. asintótica	.483

a. Tratamiento2 = Metformina

b. Prueba de Kruskal Wallis

c. Variable de agrupación: GENCYP2C9

Estadísticos de prueba[a,b,c]

	CONCMET
Chi-cuadrado	6.687
gl	2
Sig. asintótica	.035

a. Tratamiento2 = Met + Glb

b. Prueba de Kruskal Wallis

c. Variable de agrupación: GENCYP2C9

*1/*1 vs *1/*2	*1/*1 vs *1/*3	*1/*2 vs *1/*3

Estadísticos de prueba[a,b] (*1/*1 vs *1/*2)

	CONCMET
U de Mann-Whitney	15.000
W de Wilcoxon	25.000
Z	-2.437
Sig. asintótica (bilateral)	.015
Significación exacta [2* (sig. unilateral)]	.011[c]

a. Tratamiento2 = Met + Glb

b. Variable de agrupación: GENCYP2C9

c. No corregido para empates.

Estadísticos de prueba[a,b] (*1/*1 vs *1/*3)

	CONCMET
U de Mann-Whitney	54.000
W de Wilcoxon	550.000
Z	-.415
Sig. asintótica (bilateral)	.678
Significación exacta [2* (sig. unilateral)]	.708[c]

a. Tratamiento2 = Met + Glb

b. Variable de agrupación: GENCYP2C9

c. No corregido para empates.

Estadísticos de prueba[a,b] (*1/*2 vs *1/*3)

	CONCMET
U de Mann-Whitney	.000
W de Wilcoxon	10.000
Z	-2.309
Sig. asintótica (bilateral)	.021
Significación exacta [2* (sig. unilateral)]	.029[c]

a. Tratamiento2 = Met + Glb

b. Variable de agrupación: GENCYP2C9

c. No corregido para empates.

IVS8

METFORMIN

Resumen de procesamiento de casos[a]

	IVS8	Casos					
		Válido		Perdidos		Total	
		N	Porcentaje	N	Porcentaje	N	Porcentaje
CONCMET	AA	27	77.1%	8	22.9%	35	100.0%
	AT	15	78.9%	4	21.1%	19	100.0%
	TT	4	80.0%	1	20.0%	5	100.0%

a. Tratamiento2 = Metformina

METFORMIN + GLIBENCLAMIDE

Resumen de procesamiento de casos[a]

	IVS8	Casos					
		Válido		Perdidos		Total	
		N	Porcentaje	N	Porcentaje	N	Porcentaje
CONCMET	AA	30	88.2%	4	11.8%	34	100.0%
	AT	8	100.0%	0	0.0%	8	100.0%
	TT	2	100.0%	0	0.0%	2	100.0%

a. Tratamiento2 = Met + Glb

NORMALITY TEST

Pruebas de normalidad[a]

	IVS8	Kolmogorov-Smirnov[b]			Shapiro-Wilk		
		Estadístico	gl	Sig.	Estadístico	gl	Sig.
CONCMET	AA	.267	27	.000	.605	27	.000
	AT	.140	15	.200*	.912	15	.145
	TT	.391	4	.	.760	4	.048

*. Esto es un límite inferior de la significación verdadera.

a. Tratamiento2 = Metformina

b. Corrección de significación de Lilliefors

Pruebas de normalidad[a]

	IVS8	Kolmogorov-Smirnov[b]			Shapiro-Wilk		
		Estadístico	gl	Sig.	Estadístico	gl	Sig.
CONCMET	AA	.167	30	.032	.839	30	.000
	AT	.199	8	.200*	.904	8	.315
	TT	.260	2	.			

*. Esto es un límite inferior de la significación verdadera.

a. Tratamiento2 = Met + Glb

b. Corrección de significación de Lilliefors

DECRIPTIVE DATA

Percentiles[a]

		IVS8	Percentiles 5	10	25	50	75	90	95
Promedio ponderado (Definición 1)	CONCMET	AA	39.693200	62.776400	107.697900	262.175900	687.450000	1505.561400	3473.980000
		AT	26.411000	31.774994	80.594990	725.067000	1321.636000	1917.479800	.
		TT	9.018000	9.018000	34.646000	122.501000	644.635250	.	.
Bisagras de Tukey	CONCMET	AA			112.032450	262.175900	673.975000		
		AT			191.576445	725.067000	1146.322000		
		TT			60.274000	122.501000	474.247500		

a. Tratamiento2 = Metformina

Percentiles[a]

		IVS8	Percentiles 5	10	25	50	75	90	95
Promedio ponderado (Definición 1)	CONCMET	AA	35.266900	76.637591	156.410750	511.076900	1116.557000	1868.882300	2606.869050
		AT	70.242000	70.242000	212.084250	758.335000	970.895500	.	.
		TT	22.817000	22.817000	22.817000	539.247500	.	.	.
Bisagras de Tukey	CONCMET	AA			157.857000	511.076900	1088.976000		
		AT			238.234500	758.335000	910.803000		
		TT			22.817000	539.247500	1055.678000		

a. Tratamiento2 = Met + Glb

INFERENTIAL ANALYSIS

BETWEEN TREATMENT: MANN WHITNEY´S U TEST

Estadísticos de prueba[a,b]

	CONCMET
U de Mann-Whitney	323.000
W de Wilcoxon	701.000
Z	-1.311
Sig. asintótica (bilateral)	.190

a. IVS8 = AA

b. Variable de agrupación: Tratamiento2

A/A

Estadísticos de prueba[a,b]

	CONCMET
U de Mann-Whitney	58.000
W de Wilcoxon	178.000
Z	-.129
Sig. asintótica (bilateral)	.897
Significación exacta [2* (sig. unilateral)]	.925[c]

a. IVS8 = AT

b. Variable de agrupación: Tratamiento2

c. No corregido para empates.

A/T

Estadísticos de prueba[a,b]

	CONCMET
U de Mann-Whitney	3.000
W de Wilcoxon	13.000
Z	-.463
Sig. asintótica (bilateral)	.643
Significación exacta [2* (sig. unilateral)]	.800[c]

a. IVS8 = TT

b. Variable de agrupación: Tratamiento2

c. No corregido para empates.

T/T

BETWEEN GENOTYPE: KRUSKAL WALLIS TEST

Estadísticos de prueba[a,b,c]

	CONCMET
Chi-cuadrado	2.136
gl	2
Sig. asintótica	.344

a. Tratamiento2 = Metformina

b. Prueba de Kruskal Wallis

c. Variable de agrupación: IVS8

Estadísticos de prueba[a,b,c]

	CONCMET
Chi-cuadrado	.279
gl	2
Sig. asintótica	.870

a. Tratamiento2 = Met + Glb

b. Prueba de Kruskal Wallis

c. Variable de agrupación: IVS8

S1.11.6 Glycated Hemoglobin (HbA1c) according to treatment and genotype (n=103).

OCT1 rs72552763

METFORMIN

Resumen de procesamiento de casos[a]

	OCT1 rs72552763	Casos Válido N	Válido Porcentaje	Perdidos N	Perdidos Porcentaje	Total N	Total Porcentaje
HbA1c	GAT/GAT	28	100.0%	0	0.0%	28	100.0%
	del/GAT	22	100.0%	0	0.0%	22	100.0%
	del/del	9	100.0%	0	0.0%	9	100.0%

a. Tratamiento2 = Metformina

METFORMIN + GLIBENCLAMIDE

Resumen de procesamiento de casos[a]

	OCT1 rs72552763	Casos Válido N	Válido Porcentaje	Perdidos N	Perdidos Porcentaje	Total N	Total Porcentaje
HbA1c	GAT/GAT	16	100.0%	0	0.0%	16	100.0%
	del/GAT	24	100.0%	0	0.0%	24	100.0%
	del/del	4	100.0%	0	0.0%	4	100.0%

a. Tratamiento2 = Met + Glb

NORMALITY TEST

Pruebas de normalidad[a]

	OCT1 rs72552763	Kolmogorov-Smirnov[b] Estadístico	gl	Sig.	Shapiro-Wilk Estadístico	gl	Sig.
HbA1c	GAT/GAT	.274	28	.000	.737	28	.000
	del/GAT	.262	22	.000	.787	22	.000
	del/del	.398	9	.000	.666	9	.001

a. Tratamiento2 = Metformina

b. Corrección de significación de Lilliefors

Pruebas de normalidad[a]

	OCT1 rs72552763	Kolmogorov-Smirnov[b] Estadístico	gl	Sig.	Shapiro-Wilk Estadístico	gl	Sig.
HbA1c	GAT/GAT	.151	16	.200*	.929	16	.234
	del/GAT	.141	24	.200*	.951	24	.283
	del/del	.210	4	.	.960	4	.777

*. Esto es un límite inferior de la significación verdadera.

a. Tratamiento2 = Met + Glb

b. Corrección de significación de Lilliefors

DECRIPTIVE DATA

Percentiles[a]

		OCT1 rs72552763	5	10	25	50	75	90	95
Promedio ponderado (Definición 1)	HbA1c	GAT/GAT	5.345	5.490	5.650	6.050	6.950	8.500	10.960
		del/GAT	5.360	5.820	6.175	6.550	9.200	10.830	14.330
		del/del	5.900	5.900	6.300	6.500	8.750	.	.
Bisagras de Tukey	HbA1c	GAT/GAT			5.700	6.050	6.800		
		del/GAT			6.200	6.550	9.200		
		del/del			6.400	6.500	6.800		

a. Tratamiento2 = Metformina

Percentiles[a]

		OCT1 rs72552763	5	10	25	50	75	90	95
Promedio ponderado (Definición 1)	HbA1c	GAT/GAT	6.400	7.240	8.500	9.400	11.300	13.040	.
		del/GAT	5.425	6.000	7.300	8.350	10.450	12.150	12.725
		del/del	7.500	7.500	7.975	10.150	11.500	.	.
Bisagras de Tukey	HbA1c	GAT/GAT			8.500	9.400	11.200		
		del/GAT			7.300	8.350	10.400		
		del/del			8.450	10.150	11.300		

a. Tratamiento2 = Met + Glb

INFERENTIAL ANALYSIS

BETWEEN TREATMENT: MANN WHITNEY´S U TEST

Estadísticos de prueba[a,b]

	HbA1c
U de Mann-Whitney	33.500
W de Wilcoxon	439.500
Z	-4.651
Sig. asintótica (bilateral)	.000

a. OCT1 rs72552763 = GAT/GAT

b. Variable de agrupación: Tratamiento2

GAT/GAT

Estadísticos de prueba[a,b]

	HbA1c
U de Mann-Whitney	161.000
W de Wilcoxon	414.000
Z	-2.267
Sig. asintótica (bilateral)	.023

a. OCT1 rs72552763 = del/GAT

b. Variable de agrupación: Tratamiento2

Del/GAT

Estadísticos de prueba[a,b]

	HbA1c
U de Mann-Whitney	5.000
W de Wilcoxon	50.000
Z	-2.009
Sig. asintótica (bilateral)	.045
Significación exacta [2* (sig. unilateral)]	.050[c]

a. OCT1 rs72552763 = del/del

b. Variable de agrupación: Tratamiento2

c. No corregido para empates.

Del/del

BETWEEN GENOTYPE: KRUSKAL WALLIS TEST

Estadísticos de prueba[a,b,c]

	HbA1c
Chi-cuadrado	7.625
gl	2
Sig. asintótica	.022

a. Tratamiento2 = Metformina

b. Prueba de Kruskal Wallis

c. Variable de agrupación: OCT1 rs72552763

Estadísticos de prueba[a,b,c]

	HbA1c
Chi-cuadrado	3.020
gl	2
Sig. asintótica	.221

a. Tratamiento2 = Met + Glb

b. Prueba de Kruskal Wallis

c. Variable de agrupación: OCT1 rs72552763

OCT1 rs622342

METFORMIN

Resumen de procesamiento de casos[a]

	OCT1 rs622342	Casos					
		Válido		Perdidos		Total	
		N	Porcentaje	N	Porcentaje	N	Porcentaje
HbA1c	AA	25	100.0%	0	0.0%	25	100.0%
	AC	19	100.0%	0	0.0%	19	100.0%
	CC	15	100.0%	0	0.0%	15	100.0%

a. Tratamiento2 = Metformina

METFORMIN + GLIBENCLAMIDE

Resumen de procesamiento de casos[a]

	OCT1 rs622342	Casos					
		Válido		Perdidos		Total	
		N	Porcentaje	N	Porcentaje	N	Porcentaje
HbA1c	AA	16	100.0%	0	0.0%	16	100.0%
	AC	21	100.0%	0	0.0%	21	100.0%
	CC	7	100.0%	0	0.0%	7	100.0%

a. Tratamiento2 = Met + Glb

NORMALITY TEST

Pruebas de normalidad[a]

	OCT1 rs622342	Kolmogorov-Smirnov[b]			Shapiro-Wilk		
		Estadístico	gl	Sig.	Estadístico	gl	Sig.
HbA1c	AA	.279	25	.000	.709	25	.000
	AC	.290	19	.000	.814	19	.002
	CC	.284	15	.002	.772	15	.002

a. Tratamiento2 = Metformina

b. Corrección de significación de Lilliefors

Pruebas de normalidad[a]

	OCT1 rs622342	Kolmogorov-Smirnov[b]			Shapiro-Wilk		
		Estadístico	gl	Sig.	Estadístico	gl	Sig.
HbA1c	AA	.151	16	.200*	.929	16	.234
	AC	.140	21	.200*	.949	21	.324
	CC	.122	7	.200*	.982	7	.971

*. Esto es un límite inferior de la significación verdadera.

a. Tratamiento2 = Met + Glb

b. Corrección de significación de Lilliefors

DECRIPTIVE DATA

Percentiles[a]

		OCT1 rs622342	Percentiles 5	10	25	50	75	90	95
Promedio ponderado (Definición 1)	HbA1c	AA	5.330	5.460	5.700	6.000	6.650	8.980	11.140
		AC	5.300	5.500	6.100	6.400	8.300	10.200	.
		CC	5.900	6.080	6.400	6.800	9.500	12.620	.
Bisagras de Tukey	HbA1c	AA			5.800	6.000	6.500		
		AC			6.100	6.400	7.700		
		CC			6.450	6.800	9.350		

a. Tratamiento2 = Metformina

Percentiles[a]

		OCT1 rs622342	Percentiles 5	10	25	50	75	90	95
Promedio ponderado (Definición 1)	HbA1c	AA	6.400	7.240	8.500	9.400	11.300	13.040	.
		AC	5.390	6.420	7.400	8.700	10.900	12.180	12.830
		CC	5.800	5.800	6.800	8.300	10.300	.	.
Bisagras de Tukey	HbA1c	AA			8.500	9.400	11.200		
		AC			7.500	8.700	10.900		
		CC			7.150	8.300	9.850		

a. Tratamiento2 = Met + Glb

INFERENTIAL ANALYSIS

BETWEEN TREATMENT: MANN WHITNEY´S U TEST

Estadísticos de prueba[a,b]

	HbA1c
U de Mann-Whitney	30.500
W de Wilcoxon	355.500
Z	-4.534
Sig. asintótica (bilateral)	.000
Significación exacta [2*(sig. unilateral)]	.000[c]

a. OCT1 rs622342 = AA

b. Variable de agrupación: Tratamiento2

c. No corregido para empates.

A/A

Estadísticos de prueba[a,b]

	HbA1c
U de Mann-Whitney	81.000
W de Wilcoxon	271.000
Z	-3.213
Sig. asintótica (bilateral)	.001
Significación exacta [2* (sig. unilateral)]	.001[c]

a. OCT1 rs622342 = AC

b. Variable de agrupación: Tratamiento2

c. No corregido para empates.

A/C

Estadísticos de prueba[a,b]

	HbA1c
U de Mann-Whitney	40.500
W de Wilcoxon	160.500
Z	-.847
Sig. asintótica (bilateral)	.397
Significación exacta [2* (sig. unilateral)]	.407[c]

a. OCT1 rs622342 = CC

b. Variable de agrupación: Tratamiento2

c. No corregido para empates.

C/C

BETWEEN GENOTYPE: KRUSKAL WALLIS TEST

Estadísticos de prueba[a,b,c]

	HbA1c
Chi-cuadrado	9.342
gl	2
Sig. asintótica	.009

a. Tratamiento2 = Metformina

b. Prueba de Kruskal Wallis

c. Variable de agrupación: OCT1 rs622342

Estadísticos de prueba[a,b,c]

	HbA1c
Chi-cuadrado	2.203
gl	2
Sig. asintótica	.332

a. Tratamiento2 = Met + Glb

b. Prueba de Kruskal Wallis

c. Variable de agrupación: OCT1 rs622342

OCT2 rs316019

METFORMIN

Resumen de procesamiento de casos[a]

OCT2 rs316019		Casos: Válido N	Válido Porcentaje	Perdidos N	Perdidos Porcentaje	Total N	Total Porcentaje
HbA1c	CC	52	100.0%	0	0.0%	52	100.0%
	AC	6	100.0%	0	0.0%	6	100.0%
	AA	1	100.0%	0	0.0%	1	100.0%

a. Tratamiento2 = Metformina

METFORMIN + GLIBENCLAMIDE

Resumen de procesamiento de casos[a]

OCT2 rs316019		Casos: Válido N	Válido Porcentaje	Perdidos N	Perdidos Porcentaje	Total N	Total Porcentaje
HbA1c	CC	42	100.0%	0	0.0%	42	100.0%
	AC	2	100.0%	0	0.0%	2	100.0%

a. Tratamiento2 = Met + Glb

NORMALITY TEST

Pruebas de normalidad[a,c]

OCT2 rs316019		Kolmogorov-Smirnov[b] Estadístico	gl	Sig.	Shapiro-Wilk Estadístico	gl	Sig.
HbA1c	CC	.273	52	.000	.742	52	.000
	AC	.331	6	.039	.807	6	.068

a. Tratamiento2 = Metformina

b. Corrección de significación de Lilliefors

c. HbA1c es constante cuando OCT2 rs316019 = AA. Se ha omitido.

Pruebas de normalidad[a]

OCT2 rs316019		Kolmogorov-Smirnov[b] Estadístico	gl	Sig.	Shapiro-Wilk Estadístico	gl	Sig.
HbA1c	CC	.106	42	.200*	.973	42	.404
	AC	.260	2	.			

*. Esto es un límite inferior de la significación verdadera.

a. Tratamiento2 = Met + Glb

b. Corrección de significación de Lilliefors

DECRIPTIVE DATA

Percentiles[a,b]

		OCT2 rs316019	Percentiles 5	10	25	50	75	90	95
Promedio ponderado (Definición 1)	HbA1c	CC	5.365	5.530	5.925	6.400	7.250	10.580	11.240
		AC	5.800	5.800	5.800	6.250	8.375	.	.
Bisagras de Tukey	HbA1c	CC			5.950	6.400	7.200		
		AC			5.800	6.250	8.100		

a. Tratamiento2 = Metformina

b. HbA1c es constante cuando OCT2 rs316019 = AA. Se ha omitido.

Percentiles[a]

		OCT2 rs316019	Percentiles 5	10	25	50	75	90	95
Promedio ponderado (Definición 1)	HbA1c	CC	5.860	6.520	7.575	9.050	10.925	12.070	12.795
		AC	7.300	7.300	7.300	8.400	.	.	.
Bisagras de Tukey	HbA1c	CC			7.600	9.050	10.900		
		AC			7.300	8.400	9.500		

a. Tratamiento2 = Met + Glb

INFERENTIAL ANALYSIS

BETWEEN TREATMENT: MANN WHITNEY´S U TEST

Estadísticos de prueba[a,b]

	HbA1c
U de Mann-Whitney	419.000
W de Wilcoxon	1797.000
Z	-5.120
Sig. asintótica (bilateral)	.000

a. OCT2 rs316019 = CC

b. Variable de agrupación: Tratamiento2

C/C

Estadísticos de prueba[a,b]

	HbA1c
U de Mann-Whitney	2.000
W de Wilcoxon	23.000
Z	-1.341
Sig. asintótica (bilateral)	.180
Significación exacta [2*(sig. unilateral)]	.286[c]

a. OCT2 rs316019 = AC

b. Variable de agrupación: Tratamiento2

c. No corregido para empates.

A/C

Advertencias

No hay suficientes casos válidos para procesar en el archivo segmentado OCT2 rs316019=AA. No se han calculado estadísticas.

A/A

BETWEEN GENOTYPE: MANN WHITNEY´S TEST

Estadísticos de prueba[a,b]

	HbA1c
U de Mann-Whitney	147.000
W de Wilcoxon	168.000
Z	-.230
Sig. asintótica (bilateral)	.818
Significación exacta [2*(sig. unilateral)]	.832[c]

a. Tratamiento2 = Metformina

b. Variable de agrupación: OCT2 rs316019

c. No corregido para empates.

Estadísticos de prueba[a,b,c]

	HbA1c
Chi-cuadrado	.318
gl	1
Sig. asintótica	.573

a. Tratamiento2 = Met + Glb

b. Prueba de Kruskal Wallis

c. Variable de agrupación: OCT2 rs316019

OCT3 rs2076828

METFORMIN

Resumen de procesamiento de casos[a]

		Casos					
		Válido		Perdidos		Total	
	OCT3 rs2076828	N	Porcentaje	N	Porcentaje	N	Porcentaje
HbA1c	CC	46	100.0%	0	0.0%	46	100.0%
	CG	11	100.0%	0	0.0%	11	100.0%
	GG	2	100.0%	0	0.0%	2	100.0%

a. Tratamiento2 = Metformina

METFORMIN + GLIBENCLAMIDE

Resumen de procesamiento de casos[a]

		Casos					
		Válido		Perdidos		Total	
	OCT3 rs2076828	N	Porcentaje	N	Porcentaje	N	Porcentaje
HbA1c	CC	32	100.0%	0	0.0%	32	100.0%
	CG	10	100.0%	0	0.0%	10	100.0%
	GG	2	100.0%	0	0.0%	2	100.0%

a. Tratamiento2 = Met + Glb

NORMALITY TEST

Pruebas de normalidad[a]

		Kolmogorov-Smirnov[b]			Shapiro-Wilk		
	OCT3 rs2076828	Estadístico	gl	Sig.	Estadístico	gl	Sig.
HbA1c	CC	.274	46	.000	.719	46	.000
	CG	.246	11	.061	.803	11	.010
	GG	.260	2	.			

a. Tratamiento2 = Metformina

b. Corrección de significación de Lilliefors

Pruebas de normalidad[a]

		Kolmogorov-Smirnov[b]			Shapiro-Wilk		
	OCT3 rs2076828	Estadístico	gl	Sig.	Estadístico	gl	Sig.
HbA1c	CC	.086	32	.200*	.973	32	.577
	CG	.167	10	.200*	.964	10	.829
	GG	.260	2	.			

*. Esto es un límite inferior de la significación verdadera.

a. Tratamiento2 = Met + Glb

b. Corrección de significación de Lilliefors

DECRIPTIVE DATA

Percentiles[a]

			Percentiles						
		OCT3 rs2076828	5	10	25	50	75	90	95
Promedio ponderado (Definición 1)	HbA1c	CC	5.335	5.570	5.900	6.350	7.000	9.710	11.080
		CG	5.500	5.520	5.800	6.400	9.200	11.100	.
		GG	6.600	6.600	6.600	8.650	.	.	.
Bisagras de Tukey	HbA1c	CC			5.900	6.350	6.900		
		CG			5.950	6.400	8.250		
		GG			6.600	8.650	10.700		

a. Tratamiento2 = Metformina

Percentiles[a]

			Percentiles						
		OCT3 rs2076828	5	10	25	50	75	90	95
Promedio ponderado (Definición 1)	HbA1c	CC	5.885	6.520	7.525	8.950	10.575	11.670	11.970
		CG	5.800	5.970	7.650	9.900	12.375	15.420	.
		GG	7.300	7.300	7.300	8.600	.	.	.
Bisagras de Tukey	HbA1c	CC			7.550	8.950	10.550		
		CG			7.700	9.900	12.200		
		GG			7.300	8.600	9.900		

a. Tratamiento2 = Met + Glb

INFERENTIAL ANALYSIS

BETWEEN TREATMENT: MANN WHITNEY´S U TEST

Estadísticos de prueba[a,b]

	HbA1c
U de Mann-Whitney	253.500
W de Wilcoxon	1334.500
Z	-4.904
Sig. asintótica (bilateral)	.000

a. OCT3 rs2076828 = CC

b. Variable de agrupación: Tratamiento2

C/C

Estadísticos de prueba[a,b]

	HbA1c
U de Mann-Whitney	22.500
W de Wilcoxon	88.500
Z	-2.290
Sig. asintótica (bilateral)	.022
Significación exacta [2* (sig. unilateral)]	.020[c]

a. OCT3 rs2076828 = CG

b. Variable de agrupación: Tratamiento2

c. No corregido para empates.

C/G

Estadísticos de prueba[a,b]

	HbA1c
U de Mann-Whitney	2.000
W de Wilcoxon	5.000
Z	.000
Sig. asintótica (bilateral)	1.000
Significación exacta [2* (sig. unilateral)]	1.000[c]

a. OCT3 rs2076828 = GG

b. Variable de agrupación: Tratamiento2

c. No corregido para empates.

G/G

BETWEEN GENOTYPE: KRUSKAL WALLIS TEST

Estadísticos de prueba[a,b,c]

	HbA1c
Chi-cuadrado	2.184
gl	2
Sig. asintótica	.336

a. Tratamiento2 = Metformina

b. Prueba de Kruskal Wallis

c. Variable de agrupación: OCT3 rs2076828

Estadísticos de prueba[a,b,c]

	HbA1c
Chi-cuadrado	1.587
gl	2
Sig. asintótica	.452

a. Tratamiento2 = Met + Glb

b. Prueba de Kruskal Wallis

c. Variable de agrupación: OCT3 rs2076828

ABCB1 rs1128503

METFORMIN

Resumen de procesamiento de casos[a]

		Casos					
		Válido		Perdidos		Total	
	ABCB1 rs1128503	N	Porcentaje	N	Porcentaje	N	Porcentaje
HbA1c	CC	15	100.0%	0	0.0%	15	100.0%
	CT	28	100.0%	0	0.0%	28	100.0%
	TT	16	100.0%	0	0.0%	16	100.0%

a. Tratamiento2 = Metformina

METFORMIN + GLIBENCLAMIDE

Resumen de procesamiento de casos[a]

		Casos					
		Válido		Perdidos		Total	
	ABCB1 rs1128503	N	Porcentaje	N	Porcentaje	N	Porcentaje
HbA1c	CC	12	100.0%	0	0.0%	12	100.0%
	CT	17	100.0%	0	0.0%	17	100.0%
	TT	15	100.0%	0	0.0%	15	100.0%

a. Tratamiento2 = Met + Glb

NORMALITY TEST

Pruebas de normalidad[a]

		Kolmogorov-Smirnov[b]			Shapiro-Wilk		
	ABCB1 rs1128503	Estadístico	gl	Sig.	Estadístico	gl	Sig.
HbA1c	CC	.357	15	.000	.633	15	.000
	CT	.243	28	.000	.800	28	.000
	TT	.257	16	.006	.803	16	.003

a. Tratamiento2 = Metformina

b. Corrección de significación de Lilliefors

Pruebas de normalidad[a]

		Kolmogorov-Smirnov[b]			Shapiro-Wilk		
	ABCB1 rs1128503	Estadístico	gl	Sig.	Estadístico	gl	Sig.
HbA1c	CC	.137	12	.200*	.897	12	.144
	CT	.148	17	.200*	.940	17	.318
	TT	.150	15	.200*	.957	15	.649

*. Esto es un límite inferior de la significación verdadera.

a. Tratamiento2 = Met + Glb

b. Corrección de significación de Lilliefors

DECRIPTIVE DATA

Percentiles[a]

			Percentiles						
		ABCB1 rs1128503	5	10	25	50	75	90	95
Promedio ponderado (Definición 1)	HbA1c	CC	5.300	5.420	5.700	6.100	6.400	10.400	.
		CT	5.545	5.780	6.150	6.650	8.525	11.140	13.370
		TT	5.300	5.370	5.850	6.250	6.750	9.290	.
Bisagras de Tukey	HbA1c	CC			5.750	6.100	6.350		
		CT			6.200	6.650	8.450		
		TT			5.900	6.250	6.700		

a. Tratamiento2 = Metformina

Percentiles[a]

			Percentiles						
		ABCB1 rs1128503	5	10	25	50	75	90	95
Promedio ponderado (Definición 1)	HbA1c	CC	8.200	8.260	8.875	10.100	11.975	14.650	.
		CT	6.200	6.680	7.500	8.500	10.550	11.920	.
		TT	5.300	5.600	7.300	8.500	10.900	12.180	.
Bisagras de Tukey	HbA1c	CC			9.050	10.100	11.850		
		CT			7.500	8.500	10.500		
		TT			7.300	8.500	10.150		

a. Tratamiento2 = Met + Glb

INFERENTIAL ANALYSIS

BETWEEN TREATMENT: MANN WHITNEY´S U TEST

Estadísticos de prueba[a,b]

	HbA1c
U de Mann-Whitney	13.000
W de Wilcoxon	133.000
Z	-3.759
Sig. asintótica (bilateral)	.000
Significación exacta [2*(sig. unilateral)]	.000[c]

a. ABCB1 rs1128503 = CC

b. Variable de agrupación: Tratamiento2

c. No corregido para empates.

C/C

Estadísticos de prueba[a,b]

	HbA1c
U de Mann-Whitney	123.500
W de Wilcoxon	529.500
Z	-2.683
Sig. asintótica (bilateral)	.007

a. ABCB1 rs1128503 = CT

b. Variable de agrupación: Tratamiento2

C/T

Estadísticos de prueba[a,b]

	HbA1c
U de Mann-Whitney	51.500
W de Wilcoxon	187.500
Z	-2.711
Sig. asintótica (bilateral)	.007
Significación exacta [2* (sig. unilateral)]	.006[c]

a. ABCB1 rs1128503 = TT

b. Variable de agrupación: Tratamiento2

c. No corregido para empates.

T/T

BETWEEN GENOTYPE: KRUSKAL WALLIS TEST

Estadísticos de prueba[a,b,c]

	HbA1c
Chi-cuadrado	7.708
gl	2
Sig. asintótica	.021

a. Tratamiento2 = Metformina

b. Prueba de Kruskal Wallis

c. Variable de agrupación: ABCB1 rs1128503

Estadísticos de prueba[a,b,c]

	HbA1c
Chi-cuadrado	5.766
gl	2
Sig. asintótica	.056

a. Tratamiento2 = Met + Glb

b. Prueba de Kruskal Wallis

c. Variable de agrupación: ABCB1 rs1128503

ABCB1 rs2032582

METFORMIN

Resumen de procesamiento de casos[a]

	ABCB1 rs2032582	Casos					
		Válido		Perdidos		Total	
		N	Porcentaje	N	Porcentaje	N	Porcentaje
HbA1c	GG	19	100.0%	0	0.0%	19	100.0%
	GT	27	100.0%	0	0.0%	27	100.0%
	TT	9	100.0%	0	0.0%	9	100.0%
	TA	1	100.0%	0	0.0%	1	100.0%
	GA	3	100.0%	0	0.0%	3	100.0%

a. Tratamiento2 = Metformina

METFORMIN + GLIBENCLAMIDE

Resumen de procesamiento de casos[a]

	ABCB1 rs2032582	Casos					
		Válido		Perdidos		Total	
		N	Porcentaje	N	Porcentaje	N	Porcentaje
HbA1c	GG	15	100.0%	0	0.0%	15	100.0%
	GT	16	100.0%	0	0.0%	16	100.0%
	TT	8	100.0%	0	0.0%	8	100.0%
	TA	1	100.0%	0	0.0%	1	100.0%
	GA	3	100.0%	0	0.0%	3	100.0%

a. Tratamiento2 = Met + Glb

NORMALITY TEST

Pruebas de normalidad[a,c]

	ABCB1 rs2032582	Kolmogorov-Smirnov[b]			Shapiro-Wilk		
		Estadístico	gl	Sig.	Estadístico	gl	Sig.
HbA1c	GG	.347	19	.000	.659	19	.000
	GT	.227	27	.001	.850	27	.001
	TT	.397	9	.000	.619	9	.000
	GA	.292	3	.	.923	3	.463

a. Tratamiento2 = Metformina

b. Corrección de significación de Lilliefors

c. HbA1c es constante cuando ABCB1 rs2032582 = TA. Se ha omitido.

Pruebas de normalidad[a,d]

	ABCB1 rs2032582	Kolmogorov-Smirnov[b]			Shapiro-Wilk		
		Estadístico	gl	Sig.	Estadístico	gl	Sig.
HbA1c	GG	.146	15	.200*	.943	15	.424
	GT	.148	16	.200*	.962	16	.698
	TT	.187	8	.200*	.899	8	.282
	GA	.379	3	.	.766	3	.035

*. Esto es un límite inferior de la significación verdadera.

a. Tratamiento2 = Met + Glb

b. Corrección de significación de Lilliefors

d. HbA1c es constante cuando ABCB1 rs2032582 = TA. Se ha omitido.

DECRIPTIVE DATA

Percentiles[a,b]

		ABCB1 rs2032582	Percentiles 5	10	25	50	75	90	95
Promedio ponderado (Definición 1)	HbA1c	GG	5.300	5.300	5.700	6.100	6.400	10.700	.
		GT	5.440	5.580	6.400	6.800	8.100	10.460	11.340
		TT	5.600	5.600	5.900	6.200	6.350	.	.
		GA	5.900	5.900	5.900	6.500	.	.	.
Bisagras de Tukey	HbA1c	GG			5.750	6.100	6.350		
		GT			6.400	6.800	8.100		
		TT			6.000	6.200	6.300		
		GA			6.200	6.500	7.400		

a. Tratamiento2 = Metformina

b. HbA1c es constante cuando ABCB1 rs2032582 = TA. Se ha omitido.

Percentiles[a,b]

		ABCB1 rs2032582	Percentiles 5	10	25	50	75	90	95
Promedio ponderado (Definición 1)	HbA1c	GG	6.200	6.980	8.200	9.900	11.600	12.140	.
		GT	5.300	5.650	6.925	8.600	9.475	12.200	.
		TT	7.300	7.300	7.350	8.850	10.525	.	.
		GA	10.900	10.900	10.900	11.000	.	.	.
Bisagras de Tukey	HbA1c	GG			8.250	9.900	11.100		
		GT			7.050	8.600	9.450		
		TT			7.400	8.850	10.150		
		GA			10.950	11.000	13.350		

a. Tratamiento2 = Met + Glb

b. HbA1c es constante cuando ABCB1 rs2032582 = TA. Se ha omitido.

INFERENTIAL ANALYSIS
BETWEEN TREATMENT: MANN WHITNEY´S U TEST

Estadísticos de prueba[a,b]

	HbA1c
U de Mann-Whitney	32.000
W de Wilcoxon	222.000
Z	-3.835
Sig. asintótica (bilateral)	.000
Significación exacta [2* (sig. unilateral)]	.000[c]

a. ABCB1 rs2032582 = GG

b. Variable de agrupación: Tratamiento2

c. No corregido para empates.

G/G

Estadísticos de prueba[a,b]

	HbA1c
U de Mann-Whitney	140.500
W de Wilcoxon	518.500
Z	-1.899
Sig. asintótica (bilateral)	.058

a. ABCB1 rs2032582 = GT

b. Variable de agrupación: Tratamiento2

G/T

Estadísticos de prueba[a,b]

	HbA1c
U de Mann-Whitney	4.500
W de Wilcoxon	49.500
Z	-3.037
Sig. asintótica (bilateral)	.002
Significación exacta [2* (sig. unilateral)]	.001[c]

a. ABCB1 rs2032582 = TT

b. Variable de agrupación: Tratamiento2

c. No corregido para empates.

T/T

Estadísticos de prueba[a,b]

	HbA1c
U de Mann-Whitney	.000
W de Wilcoxon	6.000
Z	-1.964
Sig. asintótica (bilateral)	.050
Significación exacta [2*(sig. unilateral)]	.100[c]

a. ABCB1 rs2032582 = GA

b. Variable de agrupación: Tratamiento2

c. No corregido para empates.

G/A

BETWEEN GENOTYPE: KRUSKAL WALLIS TEST

Estadísticos de prueba[a,b]

Tratamiento2		HbA1c
Metformina	Chi-cuadrado	9.490
	gl	2
	Sig. asintótica	.009

a. Prueba de Kruskal Wallis

b. Variable de agrupación: ABCB1 rs2032582

Estadísticos de prueba[a,b]

Tratamiento2		HbA1c
MET + GLB	Chi-cuadrado	6.650
	gl	4
	Sig. asintótica	.156

a. Prueba de Kruskal Wallis

b. Variable de agrupación: ABCB1 rs2032582

GG vs GT*

Estadísticos de prueba[a]

Tratamiento2		HbA1c
Metformina	U de Mann-Whitney	137.000
	W de Wilcoxon	327.000
	Z	-2.668
	Sig. asintótica (bilateral)	.008

a. Variable de agrupación: ABCB1 rs2032582

GG vs TT

Estadísticos de prueba[a]

Tratamiento2		HbA1c
Metformina	U de Mann-Whitney	74.500
	W de Wilcoxon	264.500
	Z	-.543
	Sig. asintótica (bilateral)	.587
	Significación exacta [2*(sig. unilateral)]	.595[b]

a. Variable de agrupación: ABCB1 rs2032582

b. No corregido para empates.

GT* vs TT

Estadísticos de prueba[a]

Tratamiento2		HbA1c
Metformina	U de Mann-Whitney	58.500
	W de Wilcoxon	103.500
	Z	-2.305
	Sig. asintótica (bilateral)	.021
	Significación exacta [2*(sig. unilateral)]	.019[b]

a. Variable de agrupación: ABCB1 rs2032582

b. No corregido para empates.

ABCB1 rs1045642

METFORMIN

Resumen de procesamiento de casos

ABCB1 rs1045642		Casos					
		Válido		Perdidos		Total	
		N	Porcentaje	N	Porcentaje	N	Porcentaje
CC	HbA1c	17	100.0%	0	0.0%	17	100.0%
CT	HbA1c	29	100.0%	0	0.0%	29	100.0%
TT	HbA1c	13	100.0%	0	0.0%	13	100.0%

METFORMIN + GLIBENCLAMIDE

Resumen de procesamiento de casos

	ABCB1 rs1045642	Casos					
		Válido		Perdidos		Total	
		N	Porcentaje	N	Porcentaje	N	Porcentaje
HbA1c	CC	19	100.0%	0	0.0%	19	100.0%
	CT	17	100.0%	0	0.0%	17	100.0%
	TT	8	100.0%	0	0.0%	8	100.0%

NORMALITY TEST

Pruebas de normalidad

ABCB1 rs1045642		Kolmogorov-Smirnov[a]			Shapiro-Wilk		
		Estadístico	gl	Sig.	Estadístico	gl	Sig.
CC	HbA1c	.310	17	.000	.776	17	.001
CT	HbA1c	.261	29	.000	.792	29	.000
TT	HbA1c	.411	13	.000	.633	13	.000

a. Corrección de significación de Lilliefors

Pruebas de normalidad

	ABCB1 rs1045642	Kolmogorov-Smirnov[a]			Shapiro-Wilk		
		Estadístico	gl	Sig.	Estadístico	gl	Sig.
HbA1c	CC	.096	19	.200*	.952	19	.429
	CT	.165	17	.200*	.957	17	.571
	TT	.179	8	.200*	.932	8	.533

*. Esto es un límite inferior de la significación verdadera.

a. Corrección de significación de Lilliefors

DECRIPTIVE DATA

Percentiles

ABCB1 rs1045642			Percentiles						
			5	10	25	50	75	90	95
CC	Promedio ponderado (Definición 1)	HbA1c	5.300	5.300	5.850	6.100	7.450	9.580	.
	Bisagras de Tukey	HbA1c			5.900	6.100	6.600		
CT	Promedio ponderado (Definición 1)	HbA1c	5.450	5.600	6.000	6.500	8.350	11.100	13.200
	Bisagras de Tukey	HbA1c			6.100	6.500	8.100		
TT	Promedio ponderado (Definición 1)	HbA1c	5.600	5.680	6.050	6.200	6.400	9.360	.
	Bisagras de Tukey	HbA1c			6.100	6.200	6.400		

Percentiles

		ABCB1 rs1045642	Percentiles						
			5	10	25	50	75	90	95
Promedio ponderado (Definición 1)	HbA1c	CC	6.200	6.800	7.600	9.900	11.000	12.200	.
		CT	5.300	5.700	7.300	8.700	10.400	12.260	.
		TT	7.300	7.300	7.750	8.850	10.525	.	.
Bisagras de Tukey	HbA1c	CC			7.950	9.900	10.950		
		CT			7.300	8.700	9.400		
		TT			8.000	8.850	10.150		

INFERENTIAL ANALYSIS

BETWEEN TREATMENT: MANN WHITNEY´S U TEST

Estadísticos de prueba[a,b]

	HbA1c
U de Mann-Whitney	40.000
W de Wilcoxon	193.000
Z	-3.852
Sig. asintótica (bilateral)	.000
Significación exacta [2*(sig. unilateral)]	.000[c]

a. ABCB1 rs1045642 = CC

b. Variable de agrupación: Tratamiento2

c. No corregido para empates.

C/C

Estadísticos de prueba[a,b]

	HbA1c
U de Mann-Whitney	147.500
W de Wilcoxon	582.500
Z	-2.256
Sig. asintótica (bilateral)	.024

a. ABCB1 rs1045642 = CT

b. Variable de agrupación: Tratamiento2

C/T

Estadísticos de prueba[a,b]

	HbA1c
U de Mann-Whitney	8.000
W de Wilcoxon	99.000
Z	-3.194
Sig. asintótica (bilateral)	.001
Significación exacta [2* (sig. unilateral)]	.001[c]

a. ABCB1 rs1045642 = TT

b. Variable de agrupación: Tratamiento2

c. No corregido para empates.

T/T

BETWEEN GENOTYPE: KRUSKAL WALLIS TEST

Estadísticos de prueba[a,b,c]

	HbA1c
Chi-cuadrado	3.664
gl	2
Sig. asintótica	.160

a. Tratamiento2 = Metformina

b. Prueba de Kruskal Wallis

c. Variable de agrupación: ABCB1 rs1045642

Estadísticos de prueba[a,b]

Tratamiento2		HbA1c
MET + GLB	Chi-cuadrado	1.343
	gl	2
	Sig. asintótica	.511

a. Prueba de Kruskal Wallis

b. Variable de agrupación: ABCB1 rs1045642

CYP2C9

METFORMIN

Resumen de procesamiento de casos

GENCYP2C9		Casos					
		Válido		Perdidos		Total	
		N	Porcentaje	N	Porcentaje	N	Porcentaje
*1/*1	HbA1c	50	100.0%	0	0.0%	50	100.0%
*1/*2	HbA1c	6	100.0%	0	0.0%	6	100.0%
*1/*3	HbA1c	3	100.0%	0	0.0%	3	100.0%

METFORMIN + GLIBENCLAMIDE

Resumen de procesamiento de casos

GENCYP2C9		Casos					
		Válido		Perdidos		Total	
		N	Porcentaje	N	Porcentaje	N	Porcentaje
*1/*1	HbA1c	35	100.0%	0	0.0%	35	100.0%
*1/*2	HbA1c	4	100.0%	0	0.0%	4	100.0%
*1/*3	HbA1c	4	100.0%	0	0.0%	4	100.0%
*2/*3	HbA1c	1	100.0%	0	0.0%	1	100.0%

NORMALITY TEST

Pruebas de normalidad

GENCYP2C9		Kolmogorov-Smirnov[a]			Shapiro-Wilk		
		Estadístico	gl	Sig.	Estadístico	gl	Sig.
*1/*1	HbA1c	.271	50	.000	.742	50	.000
*1/*2	HbA1c	.260	6	.200*	.894	6	.340
*1/*3	HbA1c	.343	3	.	.842	3	.220

*. Esto es un límite inferior de la significación verdadera.

a. Corrección de significación de Lilliefors

Pruebas de normalidad[c]

GENCYP2C9		Kolmogorov-Smirnov[a]			Shapiro-Wilk		
		Estadístico	gl	Sig.	Estadístico	gl	Sig.
*1/*1	HbA1c	.104	35	.200*	.943	35	.070
*1/*2	HbA1c	.173	4	.	.996	4	.984
*1/*3	HbA1c	.320	4	.	.905	4	.457

*. Esto es un límite inferior de la significación verdadera.

a. Corrección de significación de Lilliefors

c. HbA1c es constante en uno o más archivos segmentados. Se ha omitido.

DECRIPTIVE DATA

Percentiles

GENCYP2C9			Percentiles						
			5	10	25	50	75	90	95
*1/*1	Promedio ponderado (Definición 1)	HbA1c	5.455	5.600	5.900	6.400	7.300	10.660	11.280
	Bisagras de Tukey	HbA1c			5.900	6.400	7.300		
*1/*2	Promedio ponderado (Definición 1)	HbA1c	5.300	5.300	5.450	6.250	6.900	.	.
	Bisagras de Tukey	HbA1c			5.500	6.250	6.500		
*1/*3	Promedio ponderado (Definición 1)	HbA1c	6.200	6.200	6.200	8.300	.	.	.
	Bisagras de Tukey	HbA1c			7.250	8.300	8.450		

Percentiles[a]

GENCYP2C9			Percentiles						
			5	10	25	50	75	90	95
*1/*1	Promedio ponderado (Definición 1)	HbA1c	6.360	7.100	7.500	9.200	10.900	11.940	12.900
	Bisagras de Tukey	HbA1c			7.550	9.200	10.750		
*1/*2	Promedio ponderado (Definición 1)	HbA1c	5.300	5.300	6.025	8.800	11.425	.	.
	Bisagras de Tukey	HbA1c			6.750	8.800	10.750		
*1/*3	Promedio ponderado (Definición 1)	HbA1c	5.800	5.800	6.425	8.300	11.750	.	.
	Bisagras de Tukey	HbA1c			7.050	8.300	10.600		

a. HbA1c es constante en uno o más archivos segmentados. Se ha omitido.

INFERENTIAL ANALYSIS

BETWEEN TREATMENT: MANN WHITNEY´S U TEST

Estadísticos de prueba[a,b]

	HbA1c
U de Mann-Whitney	300.000
W de Wilcoxon	1575.000
Z	-5.137
Sig. asintótica (bilateral)	.000

a. GENCYP2C9 = *1/*1

b. Variable de agrupación: Tratamiento2

***1/*1**

Estadísticos de prueba[a,b]

	HbA1c
U de Mann-Whitney	5.500
W de Wilcoxon	26.500
Z	-1.390
Sig. asintótica (bilateral)	.165
Significación exacta [2* (sig. unilateral)]	.171[c]

a. GENCYP2C9 = *1/*2

b. Variable de agrupación: Tratamiento2

c. No corregido para empates.

***1/*2**

Estadísticos de prueba[a,b]

	HbA1c
U de Mann-Whitney	6.000
W de Wilcoxon	16.000
Z	.000
Sig. asintótica (bilateral)	1.000
Significación exacta [2* (sig. unilateral)]	1.000[c]

a. GENCYP2C9 = *1/*3

b. Variable de agrupación: Tratamiento2

c. No corregido para empates.

***1/*3**

BETWEEN GENOTYPE: KRUSKAL WALLIS TEST

Estadísticos de prueba[a,b]

Tratamiento2		HbA1c
Metformina	Chi-cuadrado	2.086
	gl	2
	Sig. asintótica	.352

a. Prueba de Kruskal Wallis

b. Variable de agrupación: GENCYP2C9

Estadísticos de prueba[a,b]

Tratamiento2		HbA1c
MET + GLB	Chi-cuadrado	.419
	gl	2
	Sig. asintótica	.811

a. Prueba de Kruskal Wallis

b. Variable de agrupación: GENCYP2C9

IVS8

METFORMIN

Resumen de procesamiento de casos

IVS8		Casos					
		Válido		Perdidos		Total	
		N	Porcentaje	N	Porcentaje	N	Porcentaje
AA	HbA1c	35	100.0%	0	0.0%	35	100.0%
AT	HbA1c	19	100.0%	0	0.0%	19	100.0%
TT	HbA1c	5	100.0%	0	0.0%	5	100.0%

METFORMIN + GLIBENCLAMIDE

Resumen de procesamiento de casos

	IVS8	Casos					
		Válido		Perdidos		Total	
		N	Porcentaje	N	Porcentaje	N	Porcentaje
HbA1c	AA	34	100.0%	0	0.0%	34	100.0%
	AT	8	100.0%	0	0.0%	8	100.0%
	TT	2	100.0%	0	0.0%	2	100.0%

NORMALITY TEST

Pruebas de normalidad

IVS8		Kolmogorov-Smirnov[a]			Shapiro-Wilk		
		Estadístico	gl	Sig.	Estadístico	gl	Sig.
AA	HbA1c	.299	35	.000	.745	35	.000
AT	HbA1c	.218	19	.018	.805	19	.001
TT	HbA1c	.436	5	.002	.653	5	.003

a. Corrección de significación de Lilliefors

Pruebas de normalidad

	IVS8	Kolmogorov-Smirnov[a]			Shapiro-Wilk		
		Estadístico	gl	Sig.	Estadístico	gl	Sig.
HbA1c	AA	.125	34	.193	.957	34	.196
	AT	.137	8	.200*	.957	8	.786
	TT	.260	2	.			

*. Esto es un límite inferior de la significación verdadera.

a. Corrección de significación de Lilliefors

DECRIPTIVE DATA

Percentiles

IVS8			Percentiles						
			5	10	25	50	75	90	95
AA	Promedio ponderado (Definición 1)	HbA1c	5.300	5.500	6.100	6.400	8.100	10.620	12.180
	Bisagras de Tukey	HbA1c			6.100	6.400	7.600		
AT	Promedio ponderado (Definición 1)	HbA1c	5.400	5.600	5.900	6.500	7.300	10.700	.
	Bisagras de Tukey	HbA1c			5.900	6.500	7.300		
TT	Promedio ponderado (Definición 1)	HbA1c	5.800	5.800	5.900	6.200	7.850	.	.
	Bisagras de Tukey	HbA1c			6.000	6.200	6.200		

Percentiles

		IVS8	Percentiles						
			5	10	25	50	75	90	95
Promedio ponderado (Definición 1)	HbA1c	AA	5.675	6.300	7.500	8.700	11.100	12.050	13.600
		AT	7.300	7.300	7.750	9.400	10.425	.	.
		TT	10.300	10.300	10.300	10.400	.	.	.
Bisagras de Tukey	HbA1c	AA			7.500	8.700	11.000		
		AT			8.000	9.400	10.250		
		TT			10.300	10.400	10.500		

INFERENTIAL ANALYSIS

BETWEEN TREATMENT: MANN WHITNEY´S U TEST

Estadísticos de prueba[a,b]

	HbA1c
U de Mann-Whitney	261.000
W de Wilcoxon	891.000
Z	-4.011
Sig. asintótica (bilateral)	.000

a. IVS8 = AA

b. Variable de agrupación: Tratamiento2

A/A

Estadísticos de prueba[a,b]

	HbA1c
U de Mann-Whitney	20.000
W de Wilcoxon	210.000
Z	-2.977
Sig. asintótica (bilateral)	.003
Significación exacta [2*(sig. unilateral)]	.002[c]

a. IVS8 = AT

b. Variable de agrupación: Tratamiento2

c. No corregido para empates.

A/T

Estadísticos de prueba[a,b]

	HbA1c
U de Mann-Whitney	.000
W de Wilcoxon	15.000
Z	-1.954
Sig. asintótica (bilateral)	.051
Significación exacta [2*(sig. unilateral)]	.095[c]

a. IVS8 = TT

b. Variable de agrupación: Tratamiento2

c. No corregido para empates.

T/T

BETWEEN GENOTYPE: KRUSKAL WALLIS TEST

Estadísticos de prueba[a,b]

Tratamiento2		HbA1c
Metformina	Chi-cuadrado	.427
	gl	2
	Sig. asintótica	.808

a. Prueba de Kruskal Wallis

b. Variable de agrupación: IVS8

Estadísticos de prueba[a,b]

Tratamiento2		HbA1c
MET + GLB	Chi-cuadrado	.986
	gl	2
	Sig. asintótica	.611

a. Prueba de Kruskal Wallis

b. Variable de agrupación: IVS8

S1.11.7 Dominant models: OCT1 and ABCB1

OCT1 rs72552763

1.- SIMPLE MODEL.

Variables en la ecuación

		B	Error estándar	Wald	gl	Sig.	Exp(B)	95% C.I. para EXP(B)	
								Inferior	Superior
Paso 1[a]	DOMOCT1rs72552763(1)	.398	.401	.989	1	.320	1.490	.679	3.266
	Constante	-.091	.302	.091	1	.763	.913		

a. Variables especificadas en el paso 1: DOMOCT1rs72552763.

2.- MULTIPLE MODEL.

Variables en la ecuación

		B	Error estándar	Wald	gl	Sig.	Exp(B)	95% C.I. para EXP(B)	
								Inferior	Superior
Paso 1[a]	DOMOCT1rs72552763(1)	.188	.570	.109	1	.741	1.207	.395	3.691
	Edad	-.066	.030	5.037	1	.025	.936	.883	.992
	Tiempodetratamiento	.230	.071	10.476	1	.001	1.259	1.095	1.448
	Tratamiento2(1)	2.879	.655	19.312	1	.000	17.793	4.928	64.246
	DXIMC(1)	2.116	1.033	4.194	1	.041	8.298	1.095	62.873
	Constante	-.706	1.735	.166	1	.684	.493		

a. Variables especificadas en el paso 1: DOMOCT1rs72552763, Edad, Tiempodetratamiento, Tratamiento2, DXIMC.

3.- CODOMINANT MODEL.

Variables en la ecuación

		B	Error estándar	Wald	gl	Sig.	Exp(B)	95% C.I. para EXP(B)	
								Inferior	Superior
Paso 1[a]	OCT1rs72552763			1.862	2	.394			
	OCT1rs72552763(1)	.533	.427	1.557	1	.212	1.704	.738	3.935
	OCT1rs72552763(2)	-.063	.633	.010	1	.920	.939	.272	3.246
	Constante	-.091	.302	.091	1	.763	.913		

a. Variables especificadas en el paso 1: OCT1rs72552763.

OCT1 rs622342

1.- SIMPLE MODEL.

Variables en la ecuación

		B	Error estándar	Wald	gl	Sig.	Exp(B)	95% C.I. para EXP(B)	
								Inferior	Superior
Paso 1[a]	DOMOCT1rs622342(1)	.472	.405	1.356	1	.244	1.603	.724	3.549
	Constante	-.147	.313	.219	1	.640	.864		

a. Variables especificadas en el paso 1: DOMOCT1rs622342.

2.- MULTIPLE MODEL.

Variables en la ecuación

		B	Error estándar	Wald	gl	Sig.	Exp(B)	95% C.I. para EXP(B)	
								Inferior	Superior
Paso 1[a]	Edad	-.065	.030	4.696	1	.030	.937	.884	.994
	Tiempodetratamiento	.237	.073	10.530	1	.001	1.267	1.098	1.462
	Tratamiento2(1)	2.873	.652	19.410	1	.000	17.698	4.929	63.549
	DXIMC(1)	2.156	1.014	4.515	1	.034	8.632	1.182	63.045
	DOMOCT1rs622342(1)	.649	.590	1.213	1	.271	1.914	.603	6.080
	Constante	-1.174	1.760	.445	1	.505	.309		

a. Variables especificadas en el paso 1: Edad, Tiempodetratamiento, Tratamiento2, DXIMC, DOMOCT1rs622342.

3.- CODOMINANT MODEL.

Variables en la ecuación

		B	Error estándar	Wald	gl	Sig.	Exp(B)	95% C.I. para EXP(B)	
								Inferior	Superior
Paso 1[a]	OCT1rs622342			2.233	2	.327			
	OCT1rs622342(1)	.657	.452	2.111	1	.146	1.930	.795	4.685
	OCT1rs622342(2)	.147	.529	.077	1	.782	1.158	.411	3.266
	Constante	-.147	.313	.219	1	.640	.864		

a. Variables especificadas en el paso 1: OCT1rs622342.

ABCB1 rs1128503.

1.- SIMPLE MODEL.

Variables en la ecuación

		B	Error estándar	Wald	gl	Sig.	Exp(B)	95% C.I. para EXP(B) Inferior	95% C.I. para EXP(B) Superior
Paso 1[a]	DOMABCBrs1128503(1)	.084	.449	.035	1	.851	1.088	.451	2.621
	Constante	.074	.385	.037	1	.847	1.077		

a. Variables especificadas en el paso 1: DOMABCBrs1128503.

2.- MULTIPLE MODEL.

Variables en la ecuación

		B	Error estándar	Wald	gl	Sig.	Exp(B)	95% C.I. para EXP(B) Inferior	95% C.I. para EXP(B) Superior
Paso 1[a]	Edad	-.071	.030	5.609	1	.018	.931	.878	.988
	Tiempodetratamiento	.234	.071	10.890	1	.001	1.263	1.100	1.451
	Tratamiento2(1)	2.921	.654	19.946	1	.000	18.568	5.152	66.920
	DXIMC(1)	2.205	1.073	4.224	1	.040	9.071	1.108	74.296
	DOMABCBrs1128503(1)	.597	.665	.807	1	.369	1.817	.494	6.691
	Constante	-.896	1.724	.270	1	.603	.408		

a. Variables especificadas en el paso 1: Edad, Tiempodetratamiento, Tratamiento2, DXIMC, DOMABCBrs1128503.

3.- CODOMINANT MODEL.

Variables en la ecuación

		B	Error estándar	Wald	gl	Sig.	Exp(B)	95% C.I. para EXP(B) Inferior	95% C.I. para EXP(B) Superior
Paso 1[a]	ABCB1rs1128503			.684	2	.710			
	ABCB1rs1128503(1)	.240	.489	.240	1	.624	1.271	.487	3.316
	ABCB1rs1128503(2)	-.139	.527	.069	1	.792	.871	.310	2.445
	Constante	.074	.385	.037	1	.847	1.077		

a. Variables especificadas en el paso 1: ABCB1rs1128503.

ABCB1 rs2032582.

1.- SIMPLE MODEL.

Variables en la ecuación

		B	Error estándar	Wald	gl	Sig.	Exp(B)	95% C.I. para EXP(B)	
								Inferior	Superior
Paso 1[a]	DOMABCBrs2032582(1)	.177	.421	.177	1	.674	1.194	.523	2.722
	Constante	.000	.343	.000	1	1.000	1.000		

a. Variables especificadas en el paso 1: DOMABCBrs2032582.

2.- MULTIPLE MODEL.

Variables en la ecuación

		B	Error estándar	Wald	gl	Sig.	Exp(B)	95% C.I. para EXP(B)	
								Inferior	Superior
Paso 1[a]	Tratamiento2(1)	2.903	.660	19.328	1	.000	18.234	4.998	66.529
	DXIMC(1)	2.138	1.051	4.135	1	.042	8.484	1.080	66.620
	Tiempodetratamiento	.236	.072	10.799	1	.001	1.266	1.100	1.458
	Edad	-.073	.030	5.813	1	.016	.930	.876	.986
	DOMABCBrs2032582(1)	.659	.613	1.157	1	.282	1.933	.582	6.425
	Constante	-.758	1.680	.204	1	.652	.469		

a. Variables especificadas en el paso 1: Tratamiento2, DXIMC, Tiempodetratamiento, Edad, DOMABCBrs2032582.

3.- CODOMINANT MODEL.

Variables en la ecuación

		B	Error estándar	Wald	gl	Sig.	Exp(B)	95% C.I. para EXP(B)	
								Inferior	Superior
Paso 1[a]	ABCB1rs2032582			.574	4	.966			
	ABCB1rs2032582(1)	.047	.459	.010	1	.919	1.048	.426	2.576
	ABCB1rs2032582(2)	.118	.595	.039	1	.843	1.125	.351	3.609
	ABCB1rs2032582(3)	21.203	28420.721	.000	1	.999	1615474843	.000	.
	ABCB1rs2032582(4)	.693	.931	.554	1	.457	2.000	.322	12.414
	Constante	.000	.343	.000	1	1.000	1.000		

a. Variables especificadas en el paso 1: ABCB1rs2032582.

S1.11.8 Daily metformin dose mg/kg/day among DMT2 according to treatment and genotype).

ABCB1 rs1128503

METFORMIN

Resumen de procesamiento de casos

	ABCB1 rs1128503	Casos					
		Válido		Perdidos		Total	
		N	Porcentaje	N	Porcentaje	N	Porcentaje
mgkgdía	CC	14	93.3%	1	6.7%	15	100.0%
	CT	27	96.4%	1	3.6%	28	100.0%
	TT	15	93.8%	1	6.3%	16	100.0%

METFORMIN + GLIBENCLAMIDE

Resumen de procesamiento de casos

	ABCB1 rs1128503	Casos					
		Válido		Perdidos		Total	
		N	Porcentaje	N	Porcentaje	N	Porcentaje
mgkgdía	CC	12	100.0%	0	0.0%	12	100.0%
	CT	17	100.0%	0	0.0%	17	100.0%
	TT	14	93.3%	1	6.7%	15	100.0%

NORMALITY TEST

Pruebas de normalidad (METFORMIN)

	ABCB1 rs1128503	Kolmogorov-Smirnov[a]			Shapiro-Wilk		
		Estadístico	gl	Sig.	Estadístico	gl	Sig.
mgkgdía	CC	.139	14	.200*	.944	14	.476
	CT	.151	27	.119	.915	27	.030
	TT	.197	15	.120	.915	15	.164

*. Esto es un límite inferior de la significación verdadera.

a. Corrección de significación de Lilliefors

Pruebas de normalidad (METFORMIN + GLIBENCLAMIDE)

	ABCB1 rs1128503	Kolmogorov-Smirnov[a]			Shapiro-Wilk		
		Estadístico	gl	Sig.	Estadístico	gl	Sig.
mgkgdía	CC	.141	12	.200*	.950	12	.636
	CT	.081	17	.200*	.983	17	.978
	TT	.185	14	.200*	.953	14	.615

*. Esto es un límite inferior de la significación verdadera.

a. Corrección de significación de Lilliefors

DECRIPTIVE DATA

Percentiles (METFORMIN)

		ABCB1 rs1128503	Percentiles						
			5	10	25	50	75	90	95
Promedio ponderado (Definición 1)	mgkgdía	CC	8.5000	9.3450	12.0775	18.7850	25.6075	35.4400	.
		CT	9.8320	9.8800	11.9200	19.1200	26.6800	30.3200	37.1760
		TT	7.7200	8.5600	10.5800	20.0000	27.4600	38.9900	.
Bisagras de Tukey	mgkgdía	CC			12.2800	18.7850	25.4100		
		CT			11.9250	19.1200	26.0800		
		TT			11.7850	20.0000	24.4200		

Percentiles (METFORMIN + GLIBENCLAMIDE)

		ABCB1 rs1128503	Percentiles						
			5	10	25	50	75	90	95
Promedio ponderado (Definición 1)	mgkgdía	CC	10.8600	11.7150	16.1050	26.9800	35.2475	42.2090	.
		CT	12.0000	13.0880	22.7050	29.2700	36.4050	41.9160	.
		TT	5.1100	8.6250	16.1950	29.7050	34.1625	53.1550	.
Bisagras de Tukey	mgkgdía	CC			18.1900	26.9800	34.5350		
		CT			23.1600	29.2700	35.3100		
		TT			16.6600	29.7050	31.9500		

INFERENTIAL ANALYSIS

BETWEEN TREATMENT: MANN WHITNEY´S U TEST

Estadísticos de prueba[a]

ABCB1 rs1128503		mgkgdía
CC	U de Mann-Whitney	47.000
	W de Wilcoxon	152.000
	Z	-1.903
	Sig. asintótica (bilateral)	.057
	Significación exacta [2* (sig. unilateral)]	.060[b]
CT	U de Mann-Whitney	97.000
	W de Wilcoxon	475.000
	Z	-3.194
	Sig. asintótica (bilateral)	.001
TT	U de Mann-Whitney	70.000
	W de Wilcoxon	190.000
	Z	-1.528
	Sig. asintótica (bilateral)	.127
	Significación exacta [2* (sig. unilateral)]	.134[b]

a. Variable de agrupación: Tratamiento2

b. No corregido para empates.

BETWEEN GENOTYPE: KRUSKAL WALLIS TEST

Estadísticos de prueba[a,b]

	mgkgdía
Chi-cuadrado	.062
gl	2
Sig. asintótica	.969

a. Prueba de Kruskal Wallis

b. Variable de agrupación: ABCB1 rs1128503

Estadísticos de prueba[a,b]

	mgkgdía
Chi-cuadrado	.058
gl	2
Sig. asintótica	.971

a. Prueba de Kruskal Wallis

b. Variable de agrupación: ABCB1 rs1128503

ABCB1 rs2032582

METFORMIN

Resumen de procesamiento de casos

	ABCB1 rs2032582	Casos					
		Válido		Perdidos		Total	
		N	Porcentaje	N	Porcentaje	N	Porcentaje
mgkgdía	GG	17	89.5%	2	10.5%	19	100.0%
	GT	26	96.3%	1	3.7%	27	100.0%
	TT	9	100.0%	0	0.0%	9	100.0%
	TA	1	100.0%	0	0.0%	1	100.0%
	GA	3	100.0%	0	0.0%	3	100.0%

METFORMIN + GLIBENCLAMIDE

Resumen de procesamiento de casos

	ABCB1 rs2032582	Casos					
		Válido		Perdidos		Total	
		N	Porcentaje	N	Porcentaje	N	Porcentaje
mgkgdía	GG	15	100.0%	0	0.0%	15	100.0%
	GT	16	100.0%	0	0.0%	16	100.0%
	TT	7	87.5%	1	12.5%	8	100.0%
	TA	1	100.0%	0	0.0%	1	100.0%
	GA	3	100.0%	0	0.0%	3	100.0%

NORMALITY TEST

Pruebas de normalidad[c]

	ABCB1 rs2032582	Kolmogorov-Smirnov[a]			Shapiro-Wilk		
		Estadístico	gl	Sig.	Estadístico	gl	Sig.
mgkgdía	GG	.210	17	.044	.895	17	.056
	GT	.099	26	.200*	.940	26	.138
	TT	.252	9	.104	.925	9	.439
	GA	.286	3	.	.931	3	.491

*. Esto es un límite inferior de la significación verdadera.

a. Corrección de significación de Lilliefors

c. mgkgdía es constante cuando ABCB1 rs2032582 = TA. Se ha omitido.

Pruebas de normalidad[c]

	ABCB1 rs2032582	Kolmogorov-Smirnov[a]			Shapiro-Wilk		
		Estadístico	gl	Sig.	Estadístico	gl	Sig.
mgkgdía	GG	.110	15	.200*	.959	15	.678
	GT	.110	16	.200*	.972	16	.874
	TT	.203	7	.200*	.970	7	.900
	GA	.248	3	.	.969	3	.660

*. Esto es un límite inferior de la significación verdadera.

a. Corrección de significación de Lilliefors

c. mgkgdía es constante cuando ABCB1 rs2032582 = TA. Se ha omitido.

DECRIPTIVE DATA

Percentiles[a]

		ABCB1 rs2032582	Percentiles 5	10	25	50	75	90	95
Promedio ponderado (Definición 1)	mgkgdía	GG	8.5000	9.8520	11.9250	16.4800	24.7400	33.3460	.
		GT	9.3860	9.8800	12.9500	19.7800	27.3625	35.0880	40.1290
		TT	7.7200	7.7200	13.5300	20.0000	27.0650	.	.
		GA	9.8000	9.8000	9.8000	10.2400	.	.	.
Bisagras de Tukey	mgkgdía	GG			11.9300	16.4800	23.2800		
		GT			12.9900	19.7800	27.3300		
		TT			16.4800	20.0000	21.1900		
		GA			10.0200	10.2400	10.8550		

a. mgkgdía es constante cuando ABCB1 rs2032582 = TA. Se ha omitido.

Percentiles[a]

		ABCB1 rs2032582	Percentiles 5	10	25	50	75	90	95
Promedio ponderado (Definición 1)	mgkgdía	GG	10.8600	12.3600	20.3500	26.9800	33.1100	43.8440	.
		GT	5.1100	9.9330	14.6800	24.3550	34.0700	45.2440	.
		TT	14.8000	14.8000	29.2000	31.9500	40.8000	.	.
		GA	24.7800	24.7800	24.7800	32.1100	.	.	.
Bisagras de Tukey	mgkgdía	GG			21.3550	26.9800	32.0450		
		GT			15.3400	24.3550	32.8300		
		TT			29.7750	31.9500	39.1500		
		GA			28.4450	32.1100	34.0350		

a. mgkgdía es constante cuando ABCB1 rs2032582 = TA. Se ha omitido.

INFERENTIAL ANALYSIS

BETWEEN TREATMENT: MANN WHITNEY´S U TEST

Estadísticos de prueba[a]

ABCB1 rs2032582		mgkgdía
GG	U de Mann-Whitney	60.000
	W de Wilcoxon	213.000
	Z	-2.549
	Sig. asintótica (bilateral)	.011
	Significación exacta [2* (sig. unilateral)]	.010[b]
GT	U de Mann-Whitney	155.000
	W de Wilcoxon	506.000
	Z	-1.373
	Sig. asintótica (bilateral)	.170
TT	U de Mann-Whitney	14.000
	W de Wilcoxon	59.000
	Z	-1.852
	Sig. asintótica (bilateral)	.064
	Significación exacta [2* (sig. unilateral)]	.071[b]
TA	U de Mann-Whitney	.000
	W de Wilcoxon	1.000
	Z	-1.000
	Sig. asintótica (bilateral)	.317
	Significación exacta [2* (sig. unilateral)]	1.000[b]
GA	U de Mann-Whitney	.000
	W de Wilcoxon	6.000
	Z	-1.964
	Sig. asintótica (bilateral)	.050
	Significación exacta [2* (sig. unilateral)]	.100[b]

a. Variable de agrupación: Tratamiento2

b. No corregido para empates.

BETWEEN GENOTYPE: KRUSKAL WALLIS TEST

Estadísticos de prueba[a,b]

	mgkgdía
Chi-cuadrado	5.863
gl	3
Sig. asintótica	.118

a. Prueba de Kruskal Wallis

b. Variable de agrupación: ABCB1 rs2032582

Estadísticos de prueba[a,b]

	mgkgdía
Chi-cuadrado	3.242
gl	3
Sig. asintótica	.356

a. Prueba de Kruskal Wallis

b. Variable de agrupación: ABCB1 rs2032582

ABCB1 rs1045642

METFORMIN

Resumen de procesamiento de casos

	ABCB1 rs1045642	Casos					
		Válido		Perdidos		Total	
		N	Porcentaje	N	Porcentaje	N	Porcentaje
mgkgdía	CC	16	94.1%	1	5.9%	17	100.0%
	CT	27	93.1%	2	6.9%	29	100.0%
	TT	13	100.0%	0	0.0%	13	100.0%

METFORMIN + GLIBENCLAMIDE

Resumen de procesamiento de casos

	ABCB1 rs1045642	Casos					
		Válido		Perdidos		Total	
		N	Porcentaje	N	Porcentaje	N	Porcentaje
mgkgdía	CC	19	100.0%	0	0.0%	19	100.0%
	CT	17	100.0%	0	0.0%	17	100.0%
	TT	7	87.5%	1	12.5%	8	100.0%

NORMALITY TEST

Pruebas de normalidad

	ABCB1 rs1045642	Kolmogorov-Smirnov[a]			Shapiro-Wilk		
		Estadístico	gl	Sig.	Estadístico	gl	Sig.
mgkgdía	CC	.279	16	.002	.857	16	.017
	CT	.112	27	.200*	.931	27	.072
	TT	.231	13	.056	.889	13	.096

*. Esto es un límite inferior de la significación verdadera.

a. Corrección de significación de Lilliefors

Pruebas de normalidad

	ABCB1 rs1045642	Kolmogorov-Smirnov[a]			Shapiro-Wilk		
		Estadístico	gl	Sig.	Estadístico	gl	Sig.
mgkgdía	CC	.090	19	.200*	.976	19	.887
	CT	.142	17	.200*	.958	17	.601
	TT	.174	7	.200*	.962	7	.836

*. Esto es un límite inferior de la significación verdadera.

a. Corrección de significación de Lilliefors

DECRIPTIVE DATA

Percentiles

		ABCB1 rs1045642	Percentiles						
			5	10	25	50	75	90	95
Promedio ponderado (Definición 1)	mgkgdía	CC	8.5000	8.9340	10.7725	12.2950	22.7725	32.2470	.
		CT	9.8800	10.1280	12.8700	19.5600	27.4600	34.3920	40.1160
		TT	7.7200	8.8640	13.6650	20.0000	23.3000	38.6260	.
Bisagras de Tukey	mgkgdía	CC			11.0050	12.2950	22.2650		
		CT			12.9300	19.5600	27.3950		
		TT			14.5000	20.0000	21.1900		

Percentiles

		ABCB1 rs1045642	Percentiles						
			5	10	25	50	75	90	95
Promedio ponderado (Definición 1)	mgkgdía	CC	10.8600	13.3600	22.3600	27.8600	35.3100	42.5000	.
		CT	5.1100	10.6220	15.3400	25.5500	31.1500	44.2860	.
		TT	14.8000	14.8000	29.2000	37.5000	40.8000	.	.
Bisagras de Tukey	mgkgdía	CC			23.2100	27.8600	33.7100		
		CT			16.6600	25.5500	30.3500		
		TT			29.7750	37.5000	39.7150		

INFERENTIAL ANALYSIS

BETWEEN TREATMENT: MANN WHITNEY´S U TEST

Estadísticos de prueba[a]

ABCB1 rs1045642		mgkgdía
CC	U de Mann-Whitney	55.000
	W de Wilcoxon	191.000
	Z	-3.212
	Sig. asintótica (bilateral)	.001
	Significación exacta [2* (sig. unilateral)]	.001[b]
CT	U de Mann-Whitney	170.000
	W de Wilcoxon	548.000
	Z	-1.434
	Sig. asintótica (bilateral)	.152
TT	U de Mann-Whitney	16.000
	W de Wilcoxon	107.000
	Z	-2.339
	Sig. asintótica (bilateral)	.019
	Significación exacta [2* (sig. unilateral)]	.019[b]

a. Variable de agrupación: Tratamiento2

b. No corregido para empates.

BETWEEN GENOTYPE: KRUSKAL WALLIS TEST

Estadísticos de prueba[a,b]

	mgkgdía
Chi-cuadrado	2.422
gl	2
Sig. asintótica	.298

a. Prueba de Kruskal Wallis

b. Variable de agrupación: ABCB1 rs1045642

Estadísticos de prueba[a,b]

	mgkgdía
Chi-cuadrado	3.717
gl	2
Sig. asintótica	.156

a. Prueba de Kruskal Wallis

b. Variable de agrupación: ABCB1 rs1045642
